# Study of Natural Dyes’ Liposomal Encapsulation in Food Dispersion Model Systems via High-Pressure Homogenization

**DOI:** 10.3390/molecules30081845

**Published:** 2025-04-20

**Authors:** Lubomír Lapčík, Barbora Lapčíková, Tomáš Valenta, Martin Vašina, Pavlína Dudová, Miroslav Fišera

**Affiliations:** Department of Food Technology, Faculty of Technology, Tomas Bata University in Zlin, nam. T. G. Masaryka 5555, 760 01 Zlin, Czech Republic; lapcikova@utb.cz (B.L.); tvalenta@utb.cz (T.V.); vasina@utb.cz (M.V.); pavlina.dudova@gmail.com (P.D.); fisera@utb.cz (M.F.)

**Keywords:** encapsulation, natural dyes, lecithin, carboxymethylcellulose, anthocyanins, chlorophyllins, β-carotene

## Abstract

The aim of this study was to investigate the encapsulation of natural food dyes incorporated into liposomes in terms of particle size, rheological and colour properties, zeta potential, and encapsulation efficiency. The liposomes contained dye substances of anthocyanins from freeze-dried raspberry powder (R), copper complexes of chlorophyllins (C), or commercial-grade β-carotene (B). The phospholipid envelope was composed of sunflower lecithin and carboxymethylcellulose sodium salt as a surface stabilizer treated by high-pressure homogenization. The median particle diameter of R and C systems fluctuated around 200 nm, while B systems showed a broader range of 165–405 nm. The rheological results demonstrated a specific flow behaviour pattern dependent on the rotational shear applied, indicating a flow-induced structural change in the dispersions. Samples were characterized by a translucent profile with relatively high lightness, accompanied by a hue angle (h*) typical of the dye encapsulated. The zeta potential was approx. −30 mV, showing electrokinetically stabilized dispersions. The encapsulation efficiency (*EE*) varied significantly, with the highest *EE* observed for anthocyanins, ranging from 36.17 to 84.61%. The chlorophyll encapsulation was the least effective, determined in the range between 1.82 and 16.03%. Based on the suitability index, optimal liposomal formulations were evaluated by means of the Central Composite Design (CCD).

## 1. Introduction

Nanosized colloidal dispersions offer distinct advantages for the delivery of encapsulated compounds. These benefits arise from their high surface-area-to-volume ratio and unique physicochemical properties. Common nanocarriers include liposomes, micelles, polymersomes, nanoparticles, niosomes, nanogels, and dendrimers. Recent trends focus on liquid-in-liquid dispersions and relevant gels, which effectively transfer active compounds in food, cosmetic, pharmaceutical and wellness matrices [1,2,3]. Other perspective nanocolloidal systems used to encapsulate and deliver the bioactive substances are the cubosomes and hexosomes formed from lipid-based liquid crystalline phases [4,5,6]. The study by Matos and Ferreira demonstrated the enhanced colloidal stability of hexosomes and their potential for sustainable production from renewable sources such as plant-derived lipids [7].

Liposomes are spherical vesicles that are composed of a phospholipid bilayer(s) depending on the liposomal type structure. They can be characterized as self-assembled formations built from unilamellar (single bilayer) or concentric multilamellar (multiple bilayers) compositions [8]. One of the liposomes’ defining characteristics is their ability to encapsulate substances and release them in response to changing conditions. The phospholipid bilayers in liposomes can shift between solid-like and fluid-like states depending on whether the temperature is below or above a critical threshold known as the phase transition temperature. Below this point, the bilayer is solid-like and restricts the passage of polar molecules, while above this temperature, the bilayer becomes more fluid-like, i.e., more permeable [9]. This enables the rapid release of encapsulated substances from the liposomes into the surrounding environment, making these systems promising candidates for controlled compound delivery [10]. The structural and compositional characteristics of liposomes allow a high degree of their engineering in terms of size, composition, lamellarity, and surface charge. This adaptability makes liposomes well-suited for a wide range of applications in delivery systems and diagnostic techniques [11].

Dyes are organic compounds that are designed to impart colour to versatile matrices. They typically consist of an aromatic molecule that contains one or more chromophores responsible for the colour [12]. While some dyes originate from natural sources like fruits, peels, flowers, leaves, seeds, etc. [13,14], the majority of dyes employed in industry and research are synthetic and lack inherent bioactivity [15]. From a technological perspective, the application of dye additives in the food industry is rationalized by the loss of colour in some foods and beverages during their processing and storage [16]. The choice of dyes depends on various factors like consumer demand, cost, and colour stability [17], as well as on public health concerns, including dyes’ toxic/allergenic potential and safety for the consumers [18].

Natural dyes provide some advantages over artificial ones, such as antioxidant and antimicrobial activities, and potential health benefits in the prevention of some chronic diseases [19,20]. Among natural dyes, carotenoids can replace yellow and orange synthetic agents, while anthocyanins and betalains are viable alternatives for red, yellow, and purple dyes [21]. Chlorophylls are commonly used to impart a green hue. Nonetheless, natural dyes are subject to technological limitations during the processing and storage, given by a heightened susceptibility to oxidation and higher temperatures, which can lead to a significant colour loss of food products, negatively affecting their overall sensory profile. Given these challenges, it is essential to explore various encapsulation techniques using suitable coating materials that can improve the stability and shelf-life of the dyes [17]. This is achieved by reducing the interactions that occur between dye molecules and air or other constituents in the food matrix. Moreover, encapsulation provides protection against the effects of higher temperatures; reduces water activity in food systems, which in turn limits microbial growth and storage/transportation costs; and permits the incorporation of the dyes into foods, where solubility or pH factors would be otherwise incompatible [19,22].

The homogenization technique is an essential process that enables dye coating with a wall material (biopolymer). In an emulsion system, the encapsulated compound is surrounded by a surfactant monolayer, which can, in turn, be further coated with polymers on the surface [23]. This processing increases the kinetic stability of emulsion systems, including simple oil-in-water (O/W) emulsions [24] as well as multiple emulsions, which are characterized by enhanced bioaccessibility and the controlled release of loaded pigments [20]. Another encapsulation technique employs self-assembled micellar nanocarriers prepared from cationic and non-ionic surfactant systems [25]. A prospective approach was published by Chatterjee et al. [26], who designed a thin film dewetting technique to generate micro-/nanoparticles, enabling an effective encapsulation of fluorescent dye molecules such as rhodamine B. Ulrich et al. [10] studied a temperature-controlled release of 5(6)-carboxy-fluorescein encapsulated in hydrogel-immobilized liposomes.

The purpose of this study was to explore the encapsulation of food dyes within liposomes using the high-pressure homogenization method. This technique enabled the preparation of liposomal carrier systems loaded with various types of dyes, including anthocyanins, copper chlorophyllin complexes, and β-carotene. The encapsulation efficiency was studied by testing various combinations of water, sunflower lecithin, and carboxymethylcellulose sodium salt (CMC-Na). Using dynamic light scattering, the particle size and polydispersity index (*PDI*) were assessed in relation to the electrokinetic potential, which reflects the colloidal stability of the liposomal system. The rheological behaviour of the samples was evaluated through flow curve measurements, and the colour profile was characterized by trichromatic analysis. The prediction of the optimal combination of lecithin, water, and CMC-Na amounts in liposomal carriers was performed using the suitability index. Based on a scientific approach applicable to multicomponent systems, the Central Composite Design (CCD) was employed to propose experimental systems with varying ratios of the components. This design allowed us to test a limited number of potentially suitable formulations to identify the most effective design for the model dispersions.

## 2. Results and Discussion

### 2.1. Particle Size of Liposomal Dispersions

As can be seen in Table 1, the median diameter *D_Z_*_50_ was comparable for the dyes encapsulated, although B samples showed a wider range of particle size distribution. Specifically, the *D_Z_*_50_ was found in the range of approx. 165–249 nm, except for B systems No. 12–15 with a *D_Z_*_50_ of approx. 313–406 nm. The higher *D_Z_*_50_ values determined for the B systems No. 12–15 could be associated with a higher content of lecithin, forming, in the presence of polymer (CMC-Na), more stable and larger liposomes. The results observed for the B samples also indicate the presence of larger liposomes, with sizes greater than 100 nm, in the dispersions [27].

The degree of consistency in particle size was characterized by the span, a parameter that represents the width of the particle size distribution curve. It was evident that C systems showed more uniform particles owing to a narrow span range between 0.5872 and 0.6735. On the other side, larger span values were determined for R samples in the range of 0.6813–1.2613 and for B samples in the range of 0.8017–1.2302, indicating a wider distribution of particle sizes in R and B dispersions, as shown in Table 1.

For the polydispersity index *PDI*, the differences between the samples were statistically insignificant (*p* ˃ 0.05, Tukey test), fluctuating in a relatively narrow range of 0.221–0.450, with the majority of *PDI* values below 0.300. Only two samples (R systems No. 5 and 6) had *PDI* values exceeding 0.400. The data in this study were comparable with the particle size distribution in liposomal formulations with encapsulated dye compounds presented by other authors [28,29]. The particle sizes indicated that the dispersions showed a similar polydisperse character, most probably due to the aggregation effect. Several R and B samples also contained fractions with particle sizes higher than 1000 nm, indicating the presence of giant liposomes [27]. Interestingly, the largest fraction of the particle size, 4689.8 ± 154.2 nm, was observed in the B system No. 14, probably as a result of the highest water ratio applied, providing a higher volume available at the liposomes’ formation.

### 2.2. Rheological Behaviour of Dispersion Systems

**Figure 1:**
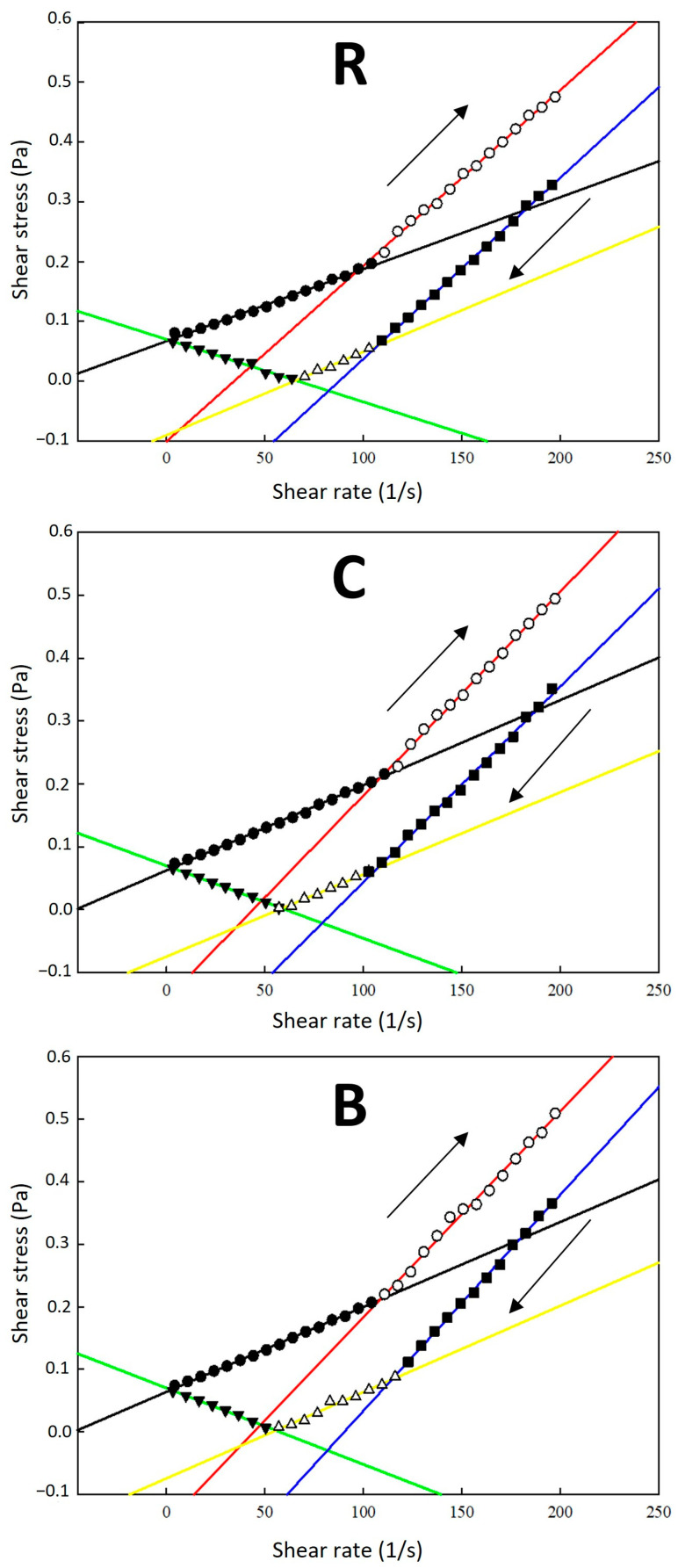
Shear stress vs. shear rate flow curves of liposomal dispersions (carrier system No. 1). The flow curves are divided into several segments delimited by the flow behaviour change and represented by symbols: (●) the 1st flow section; (○) the 2nd flow section; (■) the 3rd flow section; (∆) the 4th flow section; (▼) the 5th flow section. The full-colour lines represent the linear regression of the flow curve sections. The arrows indicate the increasing shear rate (↑ up) and decreasing shear rate (↓ down). Samples labelling: (R)—dispersions with raspberry anthocyanins; (C)—dispersions with chlorophylls; (B)—dispersions with β-carotene.

For all samples under study, two distinct sections were observed on the flow curves in the shear rate range of 0.1–200 s⁻^1^: shear-thinning behaviour at low shear rates and shear-thickening behaviour as the shear rate increased beyond a critical value. With further increases in the shear rate, shear-thinning behaviour related to the characteristics of the CMC-Na polymer was observed, indicating a reduction in flow resistance at higher shear rates [30]. As shown in Figure 1, the flow change was observed at specific average rates (intersection points *x*): for R samples, on upward flow curves (up) at 96.72 s^−1^ and downward flow curves (down) at 107.19 s^−1^ and 65.65 s^−1^; for C samples, at 108.63 s^−1^ (up) and 106.87 s^−1^ and 58.68 s^−1^ (down); and for B samples, at 108.58 s^−1^ (up), and 114.49 s^−1^ and 55.49 s^−1^ (down). These results indicate that the flow-induced structural change was most pronounced in R samples, showing the greatest difference between the change points (*x* values) for upward and downward shear rates, as manifested on the shear stress flow curves (Figure 1). The viscosity increase observed during the shear rate decrease at 50–0.1 s^−1^ could be explained by the percolation effect of lecithin bilayer liposomal structures forming a continuous network within the aqueous dispersions [31]. The viscosity enhancement was particularly evident in the systems with higher CMC-Na ratio: as the shearing magnitude was reduced, the recovery of the percolated structure by the re-entanglement of the long polymer chains enabled a more effective resistance of the flow [32,33], affecting the sample’s rheological profile (the rheological data are presented in Tables S1–S3, Supplementary Materials).

Using the Herschel–Bulkley model, it was evident that the dispersions’ yield stress *τ_0_* fluctuated around a value of 0.1 Pa, similar for all samples, as shown in Table 2. It may be deduced that variable formulations and the presence of different dye substances in liposomal dispersions showed statistically insignificant differences in the flow state induction stress observed for the samples (*p* ˃ 0.05, Tukey test). The consistency coefficient *K* was very low, determined in the range of 2.8–7.0 × 10^−4^ Pa·s^n^ for R samples, 2.4 × 10^−5^ to 9.9 × 10^−4^ Pa·s^n^ for C samples, and 2.5 × 10^−5^ to 1.7 × 10^−3^ Pa·s^n^ for B samples. In all cases, the flow behaviour index *n* of upward flow curves (in the complete shear rate range) was above 1.0 (Table 2), indicating a shear-thickening flow behaviour pattern.

### 2.3. Chromatic Characteristics of Dispersion Systems

**Figure 2:**
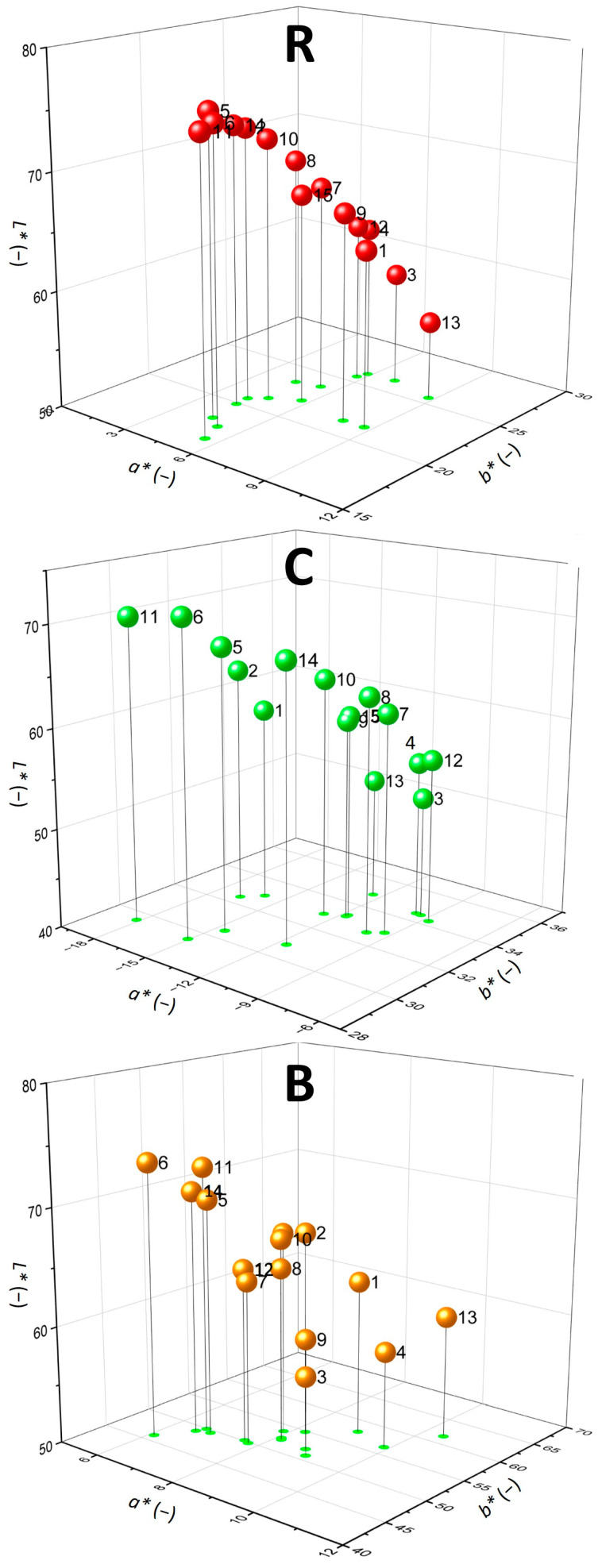
Colorimetric 3D profile of liposomal dispersions using CIE L*a*b* colour space: L*—lightness; a*—chromaticity coordinates from greenness (−) to redness (+); b*—chromaticity coordinates from blueness (−) to yellowness (+). The ball numbers represent the liposomal systems No. 1–15. Samples labelling: (R)—dispersions with raspberry anthocyanins; (C)—dispersions with chlorophylls; (B)—dispersions with β-carotene.

The CIE L*a*b* parameters of dispersion systems were illustrated in 3D graphs, as shown in Figure 2. As evident, the prevalent colour component in all samples was yellow (positive b*), with increasing b* intensity detected in the following sample order: R < C < B. The lightness L* of the samples was determined in the range from 52.13 to 75.48, with the highest L* ≥ 68 observed for systems No. 5, 6, 11 and 14. The relatively high lightness of the listed samples can be related to the lower amount of lecithin solution and/or higher amount of distilled water in the systems, increasing the translucent character of the dispersions. On the other side, the darkest colour profile was observed in System No. 3 across all sample types, with L* values ranging from 52.13 to 59.59. This is likely due to the higher proportion of lecithin solution, which naturally possesses a yellow-grey tint, contributing to a darker overall appearance. Additionally, the reduced amount of distilled water may have further concentrated the lecithin’s colour, enhancing the visual intensity of the samples.

As can be seen in Table 3, the hue angle h* of the studied dispersions was determined in a specific range typical for the dyes encapsulated. The h* observed in R samples might be characterized as a reddish yellow (red hue with yellow tones), found in the range between ca. 67.8° and 79.3°. This hue angle was consistent with the typical colour profile reported in the literature for anthocyanin-rich formulations [28], being particularly affected by the acylation process of anthocyanins in the samples [34]. For C dispersions, the h* was observed in the CIE L*a*b* quadrant section of greenish yellow (green hue with yellow tones) between 104.3° and 121.6°. For B samples, the hue angle was located in the section of deep yellow hue (80.0° to 82.4°), almost reaching full yellowness (90°). The most intense hue angle in all samples correlated with low a* (in absolute values) and high b* parameters and vice versa for the lowest h* (Table 3).

The colour differences ΔE* related to water as a reference can be considered very significant [35], increasing in the following sample order: R < C < B. In the case of R samples, the highest difference in ΔE* values was observed between the liposomal systems No. 11 and 13, whereas for C and B samples, the highest difference was between the systems No. 6 and 13. The highest colour difference determined for system No. 13 across all sample types may be explained by the lowest water content in the formulation compared to others, i.e., by the highest dye concentration (R, C, or B) in the colloidal aqueous phase. When the samples were compared, variations in the ratio of liposomal substances resulted in *noticeable* (ΔE* > 2.0), *great* (ΔE* > 6.0), or *very significant* (ΔE* > 12.0) differences in the colour profile of the dispersions, even though the same dye was encapsulated in all systems (Table 3).

### 2.4. Electrokinetic Stability of Liposomal Dispersions

**Figure 3:**
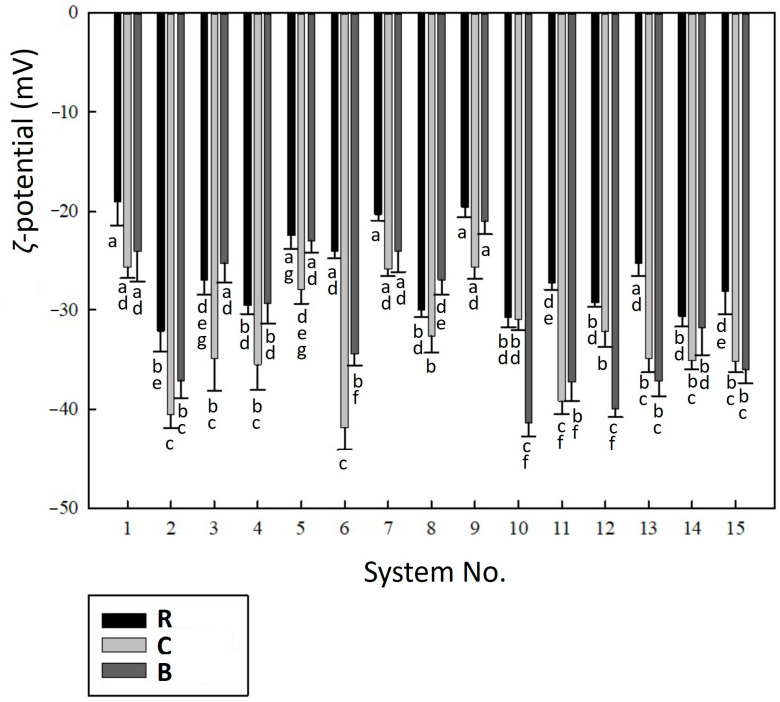
*ζ*-potential of liposomal dispersions (systems No. 1–15). Different superscript letters below the error bars (standard deviations) indicate statistically significant differences between the samples (*p* ≤ 0.05, Tukey HSD test). Samples are marked by grey-scaled colours, as illustrated in the legend. Samples labelling: R—dispersions with raspberry anthocyanins; C—dispersions with chlorophylls; B—dispersions with β-carotene.

The electrokinetic stability of liposomes was assessed by the *ζ*-potential in relation to the pH of the dispersions as the most important factor affecting the charge on the surface of liposomal particles [36]. For R samples, the pH values were determined in the range of 4.25 to 5.01, for C samples from 5.99 to 6.55, and for B samples from 5.72 to 6.39. This shows that the *ζ*-potential was assessed under relatively similar (moderately or slightly acidic) conditions. As can be seen in Figure 3, the *ζ*-potential for the majority of the studied samples fluctuated between −20 and −40 mV, with the majority around −30 mV, which is typical for electrokinetically stabilized encapsulation systems [37]. This means that the colloidal stability of the samples was relatively high, being resistant against the aggregation process [38,39] and fairly divergent from the dispersions’ isoelectric point.

As shown in Figure 3, higher *ζ*-potential values were observed for the C and B samples compared to the R samples. Specifically, systems No. 2, 6, and 10 exhibited significantly higher electrokinetic stability (*p* < 0.05, Tukey test), with *ζ*-potential values approaching approximately −40 mV. This enhanced stability may be attributed to a higher ratio of CMC-Na solution in samples No. 2, 6, and 10, which provided the encapsulation systems with a greater number of charges on the carboxymethylcellulose chains. Maximizing the ζ-potential of liposomes, in conjunction with minimizing the particle size, is known to improve the encapsulation efficiency of substances [28]. This relationship was also observed in systems No. 2, 6, and 10, which demonstrated relatively high encapsulation efficiency (as further discussed in Section 2.5) in relation to their high ζ-potential. However, it is important to note that no clear relationship was found between the ζ-potential and liposomal particle size, likely due to the relatively narrow range of median diameters observed for most dispersions (Section 2.1). The *ζ*-potential results in this study are consistent with the data for nanoliposomal encapsulation systems reported by Javadi et al. [28]. The electrokinetic stability of raspberry anthocyanin liposomes (R samples) also aligned well with the stability of blueberry anthocyanin liposomes, which exhibited a *ζ*-potential of −20.0 ± 1.0 mV, as reported by Wang et al. [29].

### 2.5. Encapsulation Efficiency of Liposomal Dispersions

**Figure 4:**
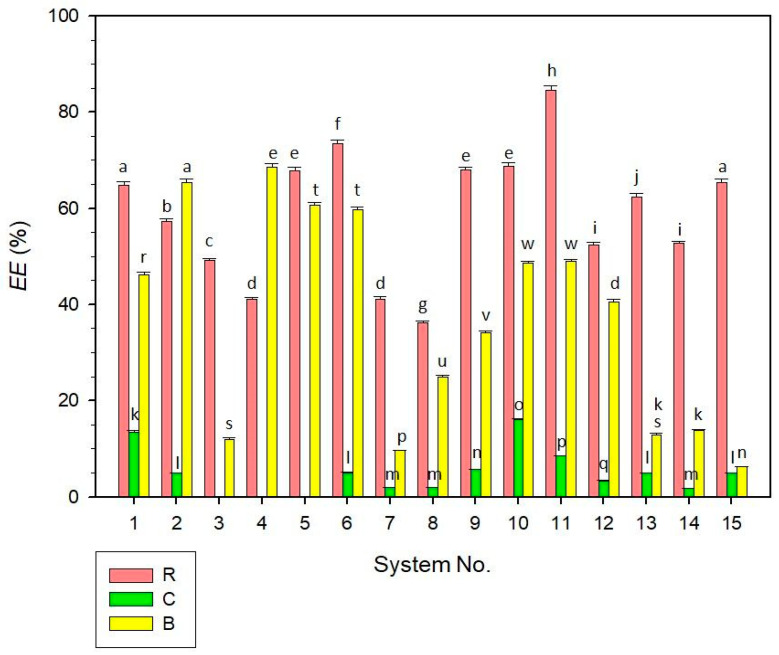
Encapsulation efficiency (*EE*) of liposomal dispersions (systems No. 1–15). Different superscript letters above the error bars (standard deviations) indicate statistically significant differences between the samples (*p* ≤ 0.05, Tukey HSD test). For the C systems No. 3–5, no relevant *EE* value was observed. Samples are marked by different colours, as illustrated in the legend. Samples labelling: R—dispersions with raspberry anthocyanins; C—dispersions with chlorophylls; B—dispersions with β-carotene.

The encapsulation efficiency (*EE*) of the studied samples is presented in Figure 4. The results varied significantly among the dyes encapsulated, with the best encapsulation observed for anthocyanins (R samples), achieving an *EE* of 84.61 ± 0.91% in system No. 11. For β-carotene (B samples), the highest encapsulation was observed in system No. 4 with an *EE* of 68.59 ± 0.71%. In contrast to this, the lowest encapsulation efficiencies of R and B samples were analysed for R system No. 8 (36.17 ± 0.34%) and B system No. 15 (6.26 ± 0.11%), respectively. As also evident from the data, B systems No. 13–15 with higher particle diameters provided substantially lower *EE* values (in the range of approx. 6–14%) compared to other B samples. This indicates that the dye encapsulation in small-size-particle systems can be more effective compared to liposomal systems with higher-sized particles [28], although the results for the studied samples were not possible to generalize due to a relatively narrow range of median diameters, as measured for most systems (Section 2.1).

Regarding the chlorophyll encapsulation (C samples), the results of *EE* were significantly lower in most cases compared to the R and B samples. For the C systems No. 3, 4, and 5, no measurable *EE* values were determined, indicating the release of chlorophyll dye into the aqueous phase. Only C systems No. 1 and 10 showed relatively higher encapsulation efficiencies, 13.51 ± 0.22% and 16.03 ± 0.25%, respectively. Other C systems had *EE* < 10%, fluctuating between 1.82 and 8.47%. The lower encapsulation efficiency of C samples could be explained by the application of different analytical methods (ICP-MS) essential for the quantification of the copper amount in the chlorophyll (as described in Section 3.7). Nonetheless, it can be expected that the application of ICP-MS effectively managed the detection of copper trace elements in complex matrices of liposomes and provided precise data, as discussed in the scientific literature [40]. Another explanation can be the degradation of chlorophyllins’ complexes [41] in the dispersions, indicating that the C systems’ formulations were not able to effectively prevent the dye release from the liposomes.

It can be assumed that the main factor affecting the encapsulation efficiency of the systems with the same encapsulated dye was the ratio of compositional substances (CMC-Na, lecithin, and water), as further discussed in Section 2.6. In addition to this, the *EE* values of the liposomal systems were influenced by the log *p* values, i.e., by the molecular 1-octanol-water partition coefficient of the dyes applied. A higher hydrophilicity of anthocyanins, indicated by lower log *p* values [42], might facilitate their incorporation within the aqueous core of liposomes, resulting in higher encapsulation efficiencies of R systems. In contrast to this, lower *EE* values observed for B and particularly C systems can be associated with higher log *p* values reflecting more lipophilicity of β-carotene [43] and chlorophyllins [44], which were characterized by lowered encapsulation in the liposomal carriers.

### 2.6. Design of Optimal Liposomal Formulation Using CCD (Central Composite Design)

The optimization of liposomal formulations was based on the best combination of lecithin, water, and CMC-Na ratios to achieve the desired properties such as encapsulation efficiency, ζ-potential, and polydispersity (particle size distribution), analysed by the Central Composite Design (CCD).

**Figure 5:**
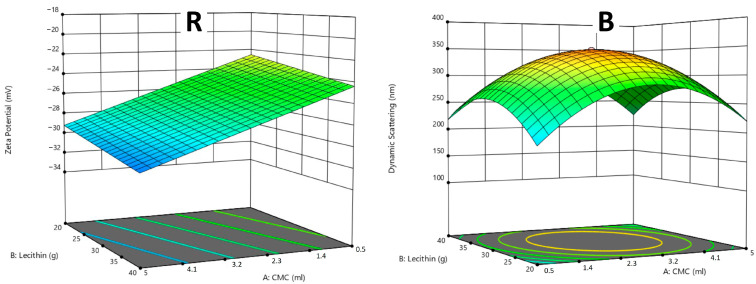
Liposomal formulation optimization by the Central Composite Design (CCD) using the response surface methodology evaluated by the 3D plot of the samples with raspberry anthocyanins (**R**) and samples with β-carotene (**B**). The CMC-Na amounts (parameter A) and lecithin amounts (parameter B) are combined with the results of ζ-potential for R samples and with dynamic light scattering (particle size) for B samples. Owing to low encapsulation efficiency, the system with chlorophyllins (**C**) was not designed.

For R samples, the design identified the best combination with a suitability index of 0.804 from 37 different options. As can be seen in Figure 5R, a higher addition of lecithin and CMC-Na resulted in an increase in ζ-potential (in absolute values), excluding the particle size parameter with a lower relevance in the design. This trend was indicative of an improved liposome profile, as the enhanced ζ-potential correlated with better stabilization of the dispersions. The optimal formulation consisted of 5 mL of CMC-Na, 20 mL of lecithin, and 60 mL of water (carrier system No. 2), showing a well-balanced system with relatively high electrokinetic stability, high anthocyanin encapsulation efficiency, and narrow particle size distribution (low *PDI*).

For B samples, the optimal combination with a suitability index of 0.652 was determined from 75 different options. As shown in Figure 5B, a dome-shaped plot indicated that the highest particle sizes were observed in samples with medium concentrations of lecithin and CMC-Na, beyond which the particle sizes may either increase or decrease. This observation suggests that an optimal range for the substances’ amount can be found on the “edges” of the 3D plot: the optimal formulation was analysed as 5 mL of CMC-Na, 20 mL of lecithin, and 90 mL of water (carrier system No. 6). The suitability index suggests that while this combination was effective for the electrokinetic stabilization and encapsulation of β-carotene (as discussed in Section 2.4. and Section 2.5.), there may be a space for particle size distribution improvement, as indicated by relatively high *D_Z_*_50_ values observed for the mentioned system (Table 2). Due to the unsatisfactorily low *EE* values determined for C samples, no design prediction was applied for the C systems. This fact highlights the importance of achieving higher chlorophyll dye encapsulation efficiency before proceeding with further optimization of the systems.

## 3. Materials and Methods

### 3.1. Materials

#### 3.1.1. Dye Agents

The dyes were ordered from the company Ekokoza CZ (Fryčovice, Czech Republic). Three different types of colouring agents were selected. Pink-red powder from freeze-dried raspberries represents the use of fruit for colouring foods. Furthermore, copper complexes of chlorophyllins, as a representative agent of a pure pigment, and commercial β-carotene, which is widely used as a food additive, were also considered. The application of dyes in food products is governed by the European Commission Regulation No. 1333/2008 on food additives [45].

Raspberry powder containing raspberries and maltodextrin was prepared by the freeze-drying of raspberries, which were ground into a coarse powder. This type of dye was chosen due to its simplicity and effectiveness: it is a natural raw material that contains anthocyanins and other pigments [46]. For the analyses, a 10% (*w*/*w*) solution of raspberry powder in distilled water was prepared. Due to the presence of raspberry seeds, the solution was filtered through the sieve before the homogenization process. Chlorophyll powder is composed of copper complexes of chlorophyllins (E141), having a green colour and characteristic odour. For the analyses, chlorophyll powder was diluted to a 1% aqueous solution. Besides the dyestuff, the commercial β-carotene contains modified starch, a mixture of coconut and rapeseed oil, maltodextrin, sunflower oil, citric acid, DL-alpha-tocopherol, ascorbyl palmitate, and potassium sorbate. As declared by the manufacturer, the content of β-carotene isolated from *Blakeslea trispora* (E160a (iii)) was 1.0 ± 0.2% (*w*/*w*) in the product, forming a viscous, orange-yellow emulsion with a carrot aroma. For its intense pigmentation, β-carotene was diluted with sunflower oil (trademark ARO; manufacturer MCC Trading International GmbH, Düsseldorf, Nordrhein-Westfalen, Germany) in a ratio of 1:1 (*v*/*v*).

#### 3.1.2. Sunflower Lecithin

Powdered sunflower lecithin (E322) was obtained by Green Medical, s.r.o. (Prague, Czech Republic). The lecithin was dissolved in distilled water and used as a 1% (*w*/*w*) solution for liposomal systems’ emulsification.

#### 3.1.3. Carboxymethylcellulose Sodium Salt (CMC-Na)

Carboxymethylcellulose sodium salt (CMC-Na, E466) was delivered by Thermo Fisher Scientific (Waltham, MA, USA). The molecular weight of the CMC-Na powder was 90 000 g/mol, the degree of substitution was 0.7, and the purity was ≥99.5%. By dissolving it in distilled water, the CMC-Na was applied as a 1% (*w*/*w*) aqueous solution with a pH of approx. 7.0 (as declared by the producer).

### 3.2. Design of Liposomal Carrier Systems Formulation and Preparation Procedure

For each dye encapsulated, a total of 15 samples with different ratios of water, lecithin and CMC-Na were prepared, as shown in Table 4. Central Composite Design (CCD) in the program Design-Expert 13 (Stat-Ease Inc., Minneapolis, MN, USA) was employed to calculate the samples’ composition based on the random combinations (20 in total) obtained by the response surface methodology (RSM). With the independent factors entered, it was possible to determine the effectiveness of the applied amount of CMC-Na (*X*_1_, 0.5–5.0 mL), lecithin (*X*_2_, 20–40 mL), and distilled water (*X*_3_, 20–90 mL), depending on the response variables: median particle size (*Y*_1_, nm), ζ-potential (*Y*_2_, mV), polydispersity (*Y*_3_, -), and encapsulation efficiency *EE* (*Y*_4_, %). To minimize the pure error, 6 repetitions were set for the centre point (sample 15). Response variables were correlated to the linear (*X_i_*), quadratic (*X_i_*^2^), and interaction (*X_i_X_j_*) independent factor terms from a second-order polynomial equation [47]:

(1) $$Y_{i}=\beta_{0}+\Sigma \beta_{i}X_{i}+\Sigma \beta_{ii}X_{i}^{2}+\beta_{ij}X_{i}X_{j}$$

where *β_0_*, *β_i_*, *β_ii_*, and *β_ij_* are the constant, main, quadratic, and interaction coefficients, respectively, of the independent factors under study [47]. All variables were analysed using linear regression, and each sample was optimized according to specified constraints: the minimization of polydispersity, the maximization of ζ-potential (in absolute values), and the maximization of encapsulation efficiency. The median particle size was limited in the range of the expected results.

At the beginning of the liposomal preparation, compounds were mixed in a given ratio (Table 4). The dye for encapsulation was applied in an equivalent amount using 0.1 mL of 10% anthocyanins (raspberry powder), 0.2 mL of 1% chlorophylls, or 20 μL of 50% β-carotene solution. The blends were homogenized using the SilentCrusher M homogenizer (Heidolph Instruments GmbH & Co. KG, Schwabach, Bavaria, Germany). Each sample was mixed for 4 min at a speed of 10,000 to 12,000 RPM at a laboratory temperature of 24 ± 1 °C in order to standardize the prepared liposomal dispersions. Homogenized samples were stored in tightly sealed glass bottles in the dark at a refrigeration temperature of 4 ± 1 °C and used for further analyses. The samples were labelled as follows: dispersions with raspberry anthocyanins (R), with chlorophylls (C), and with β-carotene (B).

### 3.3. Particle Size Determination by Dynamic Light Scattering (DLS) Measurement

DLS was used as a non-invasive technique suitable for the characterization of studied liposomal dispersions with encapsulated dyes by measuring the sizes of particles smaller than 1 μm. The ZetaPlus Zeta Potential Analyzer (Brookhaven Instruments Corporation, Holtsville, NY, USA) was used to determine the median diameter D_Z50_ (to 50% of the distribution). The span was evaluated as the distribution width, i.e., how far apart the 10 per cent and 90 per cent points are on the particle size distribution curve, normalized with the midpoint [48]. The measurements were carried out at a temperature of 23.0 ± 0.1 °C using a wavelength of 658 nm, a detection angle of 90°, and a refractive index of 1.331. The samples, before the analyses, were diluted in distilled water in a ratio of 1:1 (*v*/*v*). Each sample was measured by eight repetitions.

### 3.4. Rheological Analysis

To determine the rheological profile of the samples, rotational rheometry in the shear field was applied. The rheometer Kinexus PRO (Malvern Panalytical Ltd., Malvern, Worcestershire, UK) was employed using a bob-cup (cylinder–cylinder) arrangement with a sample volume of 17.1 mL and a gap size of 9.15 mm. The observed data were related to the rotational characteristics: the shear rate to the angular velocity of the geometry and the shear stress to the torque. The measurement was realized at an increasing shear rate of 0.1–200 s*^−^*^1^ to determine upward flow curves and subsequently at a decreasing shear rate (in the same range) for downward flow curves at the temperature of 20.0 ± 0.1 °C. The viscometric parameters were calculated using the Newton’s equation:

(2) $$\eta =\tau \cdot \dot{\gamma }$$

where *η* is the dynamic shear viscosity (Pa.s), *τ* the shear stress (Pa), and $\dot{\gamma }$ the shear rate (s*^−^*^1^) [49].

The Herschel–Bulkley model was applied to the average data of upward flow curves. The relevant parameters were calculated using the Herschel–Bulkley equation:

(3) $$\tau =\tau_{0}+K\cdot {\dot{\gamma }}^{n}$$

where *τ_0_* is the yield stress (Pa), *K* is the consistency coefficient (Pa·s^n^), and *n* is the flow behaviour index (dimensionless) [49].

The rSpace software (version 2.2), developed by Malvern Panalytical Ltd. (Malvern, Worcestershire, UK), was utilized for plotting and data validation. When evaluating the flow curves (upwards and downwards), the plots of shear stress versus shear rate were divided into five segments, through which linear regressions were fitted. The curves were intersected at the point, where the slope changed, representing a trend change of the shear (dynamic) viscosity. Using linear regression, equations for relevant lines were generated by the SigmaPlot 12.5 program (Microsoft Co., Redmond, Washington, DC, USA), and the intersections (both for upward and downward curves) were calculated using the system of several equations:

(4) $$y=k_{1}x+q_{1}$$

(5) $$y=k_{2}x+q_{2}$$

(6) $$x=(q_{2}-q_{1})/(k_{1}-k_{2})$$

where *y* (Pa) and *x* (s*^−^*^1^) are intersection points of shear stress versus shear rate curves, *k_1,2_* are the slopes of the linear equations, and *q_1,2_* are the intercepts of the linear equations.

### 3.5. CIE L*a*b* Analysis

Colorimetric analysis of dispersion samples was performed using the UltraScan VIS spectrophotometer (Hunter Associates Laboratory, Inc., Reston, VA, USA), operated in the total transmission mode. The transmission was measured under an Illuminant D65/10, simulating standard daylight conditions at an angle of 10°. Samples were poured into a 20 mm glass cuvette (transmission cell), having an optical path of 20 mm adequate for attaining reliable values of colorimetric parameters regarding the influence of the background colour [50]. Distilled water was used as a reference sample for all samples studied. The analysis was realized under controlled conditions at a laboratory temperature of 23.5 ± 0.5 °C and relative humidity of 23 ± 2%. Each sample underwent three repeated measurements.

The colourimeter was calibrated to the CIE L*a*b* colour space to quantify the dispersions’ lightness (luminosity) L*, where 0 signifies completely black and 100 completely white, along with the chromaticity coordinates a* ranging from greenness (−) to redness (+) and b* from blueness (−) to yellowness (+). Considering that the integration of a* and b* offers a more accurate and defining indication of the sample’s colour, the hue angle h* (°) was derived from the standard equation [49]:

(7) $$h^{*}=\arctan\left(b^{*}/a^{*}\right)$$

Equation (7) is valid for the first quadrant of CIE L*a*b* colour space with positive coordinates [+*a*, +*b*]. In the case of other quadrants, the calculation was adjusted to 360° representation to gain a positive sign of hue angles. Therefore, relevant modifications of Equation (7) have to be applied: for the second quadrant [−*a*, +*b*] and third quadrant [−*a*, −*b*], the hue angle h* (°) equals the sum of 180 + arctan(b*/a*), and for the fourth quadrant [+*a*, −*b*], the sum of 360 + arctan(b*/a*) [51].

Colour disparities of the samples were assessed in relation to the distilled water (reference) by the total colour difference (ΔE*). This parameter was evaluated contingent on the range of obtained values, which may be categorized as no visible difference (ΔE* < 0.2), a minor difference (0.2 < ΔE* < 2.0), a noticeable difference (2.0 < ΔE* < 6.0), a great difference (6.0 <ΔE* < 12.0), and a very significant difference (12.0 < ΔE*) [35]. The total colour difference ΔE* was computed using the following formula [52]:

(8) $$∆E^{*}=\sqrt{{\left(L_{2}^{*}-L_{1}^{*}\right)}^{2}+{\left(a_{2}^{*}-a_{1}^{*}\right)}^{2}+{\left(b_{2}^{*}-b_{1}^{*}\right)}^{2}}$$

### 3.6. ζ-Potential and pH Analyses

The ζ-potential of the liposomal dispersions was analysed using the ZetaPlus Zeta Potential Analyzer (Brookhaven Instruments Corporation, Holtsville, NY, USA), with eight repetitions for each sample. To determine the pH of the samples, the FiveEasy Plus pH meter (Mettler Toledo, Columbus, OH, USA) was used. Each sample was measured three times at a temperature of 20.0 ± 0.5 °C.

### 3.7. Encapsulation Efficiency Determination

The encapsulation efficiency (*EE*) was evaluated as the concentration of the encapsulated substance within the liposomal coating, i.e., as the ratio of the encapsulated dye amount to the total amount added. The encapsulation efficiency was determined by the spectrophotometric method, where the absorbance values of the samples with a dye, blank samples without a dye, and the supernatants after the centrifugation were measured. The weight concentration (% *w*/*w*) of free and encapsulated dyes was evaluated from the calibration series. The *EE* value (%) was computed using the following equation:

(9) $$EE\;(\%)=\frac{\left(W_{t}-BS\right)-W_{S}}{W_{t}}\cdot 100$$

where *W_t_* is the total concentration of the dye added, *BS* is the dye concentration in the blank sample, and *W_S_* is the dye concentration in the supernatant.

To obtain the supernatant, 5 mL from each sample was centrifuged using the high-speed micro-centrifuge Dlab D3024 (Dlab Scientific Co., Ltd., Beijing, China). The centrifugation process was realized at 6000 rpm for 20 min. For spectrophotometric analysis, 2.5 mL of the supernatant was taken from the upper part of the Eppendorf tube. The measurements were conducted by the UV/VIS spectrophotometer Cecil CE 1021 (Cecil Instruments, Milton Cambridge, Cambridgeshire, UK). The absorbance of the supernatant was measured in polyacrylamide four-sided clear cuvettes of 1.5 mL volume with an optical path of 10 mm at relevant detection wavelengths [53]. For the dyes under study, a specific wavelength was selected: 520 nm for the detection of anthocyanins in R samples [54] and 450 nm for the detection of β-carotene in B samples [55].

For C samples, it was impossible to determine chlorophylls at a single wavelength: the spectrophotometric method yielded negative *EE* values for some samples. For this reason, we decided to use inductively coupled plasma-mass spectrometry (ICP-MS) to assess the encapsulation effectiveness of chlorophylls. This method determined the amount of metallic elements in the samples by their incineration and the subsequent measurement of the vapours using the mass spectrometer. For the analysis, the device iCAP Q ICP-MS equipped with Qtegra software version 2.10 was used (Thermo Fisher Scientific Inc., Waltham, MA, USA). Since the C samples are copper complexes of chlorophyllins, *EE* was determined depending on the amount of copper in the samples and their supernatants [56]. The results of the *EE* for chlorophylls were calculated in a similar way as the dyes measured spectrophotometrically:

(10) $$EE\;(\%)=\frac{W_{t}-W_{S}}{W_{t}}\cdot 100$$

where *W_t_* is the total amount of the copper in the sample and *W_S_* is the copper amount in the supernatant.

### 3.8. Statistical Analysis

Parametric analyses were statistically evaluated through one-way analysis of variance (ANOVA) to assess normal data distribution, i.e., to find statistically significant differences between the samples. Mean differences among statistical groups were tested at a significance level of *p* ≤ 0.05. For multiple comparisons of the mean responses across treatment groups, the Tukey test was applied. Data analysis was performed using SigmaStat version 2.03 (Systat Software, Inc., San Jose, CA, USA) to ascertain the probability of the null hypothesis (*p*-value). Each analysed sample was measured in triplicate to ensure the accuracy and reliability of the results.

## 4. Conclusions

The present study aimed to optimize the formulation of liposomal dispersions for the effective encapsulation of natural food dyes (i.e., raspberry anthocyanins (R), chlorophylls (C), and β-carotene (B)) into liposomes. The focus was placed on key parameters including encapsulation efficiency, particle size, zeta potential, colour characteristics, and rheological properties.

It was found that liposomes containing anthocyanins and chlorophyllins exhibited median diameters fluctuating around 200 nm, whereas β-carotene-loaded liposomes showed a broader size range, approximately between 165 and 405 nm. Zeta potential measurements indicated that the dispersions were electrokinetically stabilized, with values of approx. −30 mV. Rheometric analysis showed a specific rheological profile characterized by changing flow behaviour patterns at various shear rates. Colour analysis indicated a translucent dispersion profile with hue angles characteristic of the dyes incorporated. Encapsulation efficiency varied significantly among the samples, with anthocyanins achieving the highest efficiency from 36.17 to 84.61%, and chlorophylls exhibited the lowest efficiency of 1.82–16.03%. Using the Central Composite Design (CCD), the optimal formulation for raspberry anthocyanins (with a suitability index of 0.804) was identified as 5 mL of CMC-Na, 20 mL of lecithin, and 60 mL of water. For β-carotene, the optimal combination (with a suitability index of 0.652) was 5 mL of CMC-Na, 20 mL of lecithin, and 90 mL of water. Given the evidently low encapsulation efficiency of the chlorophyllins systems, not enabling effective evaluation by the CCD, the optimal liposomal formulation was not predicted for the C systems.

This study underscores the importance of balancing lecithin, water, and CMC-Na concentrations to achieve optimal stability and encapsulation efficiency in liposomal dispersions. The results suggest that higher encapsulation efficiencies are associated with smaller particle sizes and higher ζ-potential values, indicating better electrokinetic stability. These insights provide valuable guidance for the formulation of liposomal systems and highlight their potential applications across various industries. As demonstrated by the results, the application of advanced design methods, such as Central Composite Design (CCD), enables a systematic evaluation and prediction of optimal liposomal formulations by considering variable system parameters. Future research should be focused on further refinement of these formulations and the investigation of their practical applications, particularly in the food sector.

## Figures and Tables

**Table 1:** Particle size median diameter and span of the distribution curves (carrier systems No. 1–15).

	R Samples		C Samples		B Samples	
System No.	^1^ *D_Z_*_50_ (nm)	Span (–)	*D_Z_*_50_ (nm)	Span (–)	*D_Z_*_50_ (nm)	Span (–)
1	208.4 ± 2.1 ^a^	0.972 ± 0.032 ^a^	200.7 ± 3.4 ^a^	0.648 ± 0.004 ^ad^	171.6 ± 3.4 ^a^	1.230 ± 0.011 ^a^
2	227.6 ± 3.6 ^a^	0.690 ± 0.009 ^b^	232.2 ± 4.0 ^a^	0.643 ± 0.013 ^ad^	196.1 ± 2.2 ^a^	1.095 ± 0.001 ^bd^
3	208.4 ± 4.0 ^a^	0.715 ± 0.008 ^bc^	198.0 ± 2.5 ^a^	0.625 ± 0.007 ^ac^	168.9 ± 3.0 ^a^	1.039 ± 0.012 ^bch^
4	211.1 ± 3.7 ^a^	0.805 ± 0.002 ^ce^	221.5 ± 5.0 ^a^	0.585 ± 0.007 ^b^	180.2 ± 2.9 ^a^	1.139 ± 0.014 ^cdf^
5	166.1 ± 2.9 ^a^	1.261 ± 0.049 ^d^	206.7 ± 3.3 ^a^	0.594 ± 0.005 ^bc^	172.8 ± 1.9 ^a^	1.118 ± 0.023 ^d^
6	201.1 ± 5.5 ^a^	0.972 ± 0.062 ^a^	229.1 ± 3.9 ^a^	0.606 ± 0.001 ^bc^	219.9 ± 5.9 ^a^	1.090 ± 0.016 ^bd^
7	207.2 ± 5.0 ^a^	0.738 ± 0.013 ^be^	204.1 ± 3.6 ^a^	0.626 ± 0.012 ^ac^	176.1 ± 3.0 ^a^	0.962 ± 0.009 ^e^
8	215.5 ± 3.1 ^a^	0.833 ± 0.005 ^e^	222.6 ± 4.1 ^a^	0.587 ± 0.013 ^b^	177.5 ± 2.8 ^a^	1.085 ± 0.002 ^bd^
9	213.1 ± 3.3 ^a^	0.719 ± 0.003 ^bc^	200.1 ± 3.3 ^a^	0.665 ± 0.013 ^de^	165.1 ± 1.8 ^a^	1.080 ± 0.014 ^bd^
10	246.0 ± 2.8 ^a^	0.681 ± 0.007 ^b^	219.8 ± 3.6 ^a^	0.654 ± 0.010 ^ae^	199.3 ± 3.1 ^a^	1.102 ± 0.003 ^d^
11	249.3 ± 3.0 ^a^	0.826 ± 0.050 ^e^	225.2 ± 4.5 ^a^	0.626 ± 0.016 ^ac^	204.5 ± 4.0 ^a^	1.092 ± 0.005 ^bd^
12	212.7 ± 4.2 ^a^	0.701 ± 0.002 ^bf^	206.6 ± 3.8 ^a^	0.626 ± 0.002 ^ac^	312.6 ± 5.5 ^b^	1.253 ± 0.019 ^a^
13	219.7 ± 5.0 ^a^	0.747 ± 0.012 ^be^	228.8 ± 4.0 ^a^	0.674 ± 0.016 ^de^	316.5 ± 5.9 ^b^	1.196 ± 0.014 ^af^
14	226.1 ± 3.2 ^a^	0.731 ± 0.002 ^be^	209.0 ± 2.9 ^a^	0.641 ± 0.003 ^ad^	406.4 ± 6.1 ^c^	0.802 ± 0.037 ^g^
15	228.1 ± 2.9 ^a^	0.778 ± 0.001 ^cef^	222.9 ± 3.1 ^a^	0.598 ± 0.004 ^bc^	382.1 ± 5.2 ^c^	0.981 ± 0.021 ^eh^

^1^ *D_Z_*_50_—median diameter. The values are presented as the arithmetic mean ± standard deviation of three measurements. Different superscript letters in the same column indicate statistically significant differences between the samples (*p* ≤ 0.05, Tukey test). Samples labelling: R—dispersions with raspberry anthocyanins; C—dispersions with chlorophylls; B—dispersions with β-carotene.

**Table 2:** Parameters of Herschel–Bulkley model fit for liposomal dispersions (data from complete upward flow curves).

	R Samples			C Samples			B Samples		
System No.	^1^ *τ*_0_ (Pa)	^2^ *K* (Pa.s^n^)	^3^ *n* (–)	*τ*_0_ (Pa)	*K* (Pa.s^n^)	*n* (–)	*τ*_0_ (Pa)	*K* (Pa.s^n^)	*n* (–)
1	0.083 ± 0.002 ^a^	(3.88 ± 0.21) × 10^−5 aeg^	1.76 ± 0.07 ^ab^	0.085 ± 0.002 ^a^	(2.79 ± 0.11) × 10^−5 a^	1.82 ± 0.05 ^a^	0.087 ± 0.001 ^a^	(2.75 ± 0.10) × 10^−5 a^	1.82 ± 0.04 ^ad^
2	0.099 ± 0.003 ^bcd^	(5.92 ± 0.19) × 10^−5 b^	1.69 ± 0.05 ^ab^	0.087 ± 0.001 ^af^	(5.10 ± 0.27) × 10^−4 b^	1.30 ± 0.04 ^b^	0.076 ± 0.002 ^b^	(1.14 ± 0.08) × 10^−3 b^	1.14 ± 0.03 ^b^
3	0.082 ± 0.001 ^a^	(3.10 ± 0.17) × 10^−5 cf^	1.80 ± 0.06 ^ab^	0.088 ± 0.003 ^af^	(2.41 ± 0.08) × 10^−5 a^	1.85 ± 0.07 ^a^	0.088 ± 0.003 ^ac^	(2.52 ± 0.13) × 10^−5 a^	1.84 ± 0.05 ^a^
4	0.100 ± 0.003 ^bd^	(2.77 ± 0.14) × 10^−5 c^	1.83 ± 0.04 ^a^	0.096 ± 0.002 ^be^	(2.61 ± 0.12) × 10^−5 a^	1.85 ± 0.05 ^a^	0.093 ± 0.002 ^af^	(2.36 ± 0.09) × 10^−4 cf^	1.43 ± 0.04 ^cf^
5	0.079 ± 0.001 ^a^	(3.83 ± 0.16) × 10^−5 aeg^	1.75 ± 0.05 ^ab^	0.084 ± 0.001 ^ad^	(2.80 ± 0.10) × 10^−5 a^	1.82 ± 0.06 ^a^	0.085 ± 0.002 ^cd^	(3.56 ± 0.12) × 10^−5 a^	1.77 ± 0.05 ^ad^
6	0.093 ± 0.002 ^ce^	(2.93 ± 0.11) × 10^−5 c^	1.81 ± 0.06 ^ac^	0.093 ± 0.003 ^b^	(2.11 ± 0.09) × 10^−4 c^	1.45 ± 0.03 ^c^	0.089 ± 0.003 ^ac^	(3.99 ± 0.15) × 10^−4 d^	1.34 ± 0.02 ^c^
7	0.080 ± 0.002 ^a^	(3.27 ± 0.19) × 10^−5 ceg^	1.78 ± 0.04 ^ab^	0.083 ± 0.002 ^ad^	(3.61 ± 0.16) × 10^−5 a^	1.77 ± 0.06 ^a^	0.086 ± 0.002 ^ad^	(2.67 ± 0.08) × 10^−5 a^	1.83 ± 0.06 ^a^
8	0.094 ± 0.001 ^be^	(2.87 ± 0.13) × 10^−5 c^	1.82 ± 0.05 ^ac^	0.102 ± 0.002 ^ce^	(4.06 ± 0.13) × 10^−5 a^	1.77 ± 0.04 ^a^	0.101 ± 0.003 ^eg^	(5.57 ± 0.14) × 10^−5 a^	1.71 ± 0.04 ^d^
9	0.078 ± 0.002 ^a^	(4.00 ± 0.18) × 10^−5 ae^	1.74 ± 0.03 ^ab^	0.082 ± 0.003 ^ad^	(4.62 ± 0.19) × 10^−5 a^	1.74 ± 0.05 ^a^	0.082 ± 0.002 ^bcd^	(3.70 ± 0.13) × 10^−5 a^	1.76 ± 0.03 ^ade^
10	0.102 ± 0.003 ^df^	(6.76 ± 0.29) × 10^−5 d^	1.68 ± 0.04 ^bc^	0.078 ± 0.001 ^d^	(9.92 ± 0.31) × 10^−4 d^	1.17 ± 0.02 ^b^	0.077 ± 0.001 ^b^	(1.71 ± 0.07) × 10^−3 e^	1.07 ± 0.02 ^b^
11	0.102 ± 0.002 ^df^	(4.13 ± 0.10) × 10^−5 a^	1.77 ± 0.06 ^ab^	0.102 ± 0.002 ^ce^	(4.46 ± 0.18) × 10^−5 a^	1.75 ± 0.04 ^a^	0.100 ± 0.003 ^efg^	(8.52 ± 0.28) × 10^−5 a^	1.64 ± 0.03 ^e^
12	0.100 ± 0.003 ^bd^	(3.50 ± 0.16) × 10^−5 ef^	1.79 ± 0.05 ^ab^	0.095 ± 0.002 ^b^	(2.94 ± 0.11) × 10^−5 a^	1.82 ± 0.03 ^a^	0.099 ± 0.003 ^efg^	(1.54 ± 0.10) × 10^−4 c^	1.51 ± 0.02 ^fg^
13	0.103 ± 0.004 ^d^	(7.00 ± 0.22) × 10^−5 d^	1.66 ± 0.04 ^b^	0.101 ± 0.003 ^ce^	(4.06 ± 0.15) × 10^−5 a^	1.76 ± 0.03 ^a^	0.095 ± 0.002 ^ef^	(2.43 ± 0.11) × 10^−4 f^	1.42 ± 0.04 ^cg^
14	0.093 ± 0.001 ^ce^	(3.55 ± 0.15) × 10^−5 ef^	1.78 ± 0.03 ^ab^	0.093 ± 0.002 ^bf^	(2.94 ± 0.09) × 10^−5 a^	1.82 ± 0.05 ^a^	0.100 ± 0.003 ^efg^	(4.48 ± 0.16) × 10^−5 a^	1.75 ± 0.04 ^ade^
15	0.096 ± 0.003 ^bcf^	(3.44 ± 0.13) × 10^−5 fg^	1.79 ± 0.04 ^ab^	0.098 ± 0.001 ^bc^	(2.88 ± 0.10) × 10^−5 a^	1.83 ± 0.04 ^a^	0.103 ± 0.002 ^g^	(4.39 ± 0.14) × 10^−5 a^	1.75 ± 0.06 ^ade^

^1^ *τ*_0_—yield stress; ^2^ *K*—consistency coefficient; ^3^ *n*—flow behaviour index. The values are presented as the arithmetic mean ± standard deviation of three measurements. The same superscript letters in the same column indicate statistically insignificant differences between the samples (*p* ˃ 0.05, Tukey test). Samples labelling: R—dispersions with raspberry anthocyanins; C—dispersions with chlorophylls; B—dispersions with β-carotene.

**Table 3:** Hue angle h* and total colour difference ΔE* of liposomal dispersions.

	R Samples		C Samples		B Samples	
System No.	h* (°)	ΔE* (–)	h* (°)	ΔE* (–)	h* (°)	ΔE* (–)
1	67.82 ± 0.24 ^a^	42.09 ± 0.13 ^a^	115.87 ± 0.23 ^a^	54.37 ± 0.20 ^a^	81.13 ± 0.26 ^ae^	69.08 ± 0.21 ^a^
2	78.50 ± 0.26 ^b^	34.08 ± 0.17 ^b^	117.23 ± 0.28 ^b^	51.31 ± 0.20 ^b^	81.46 ± 0.35 ^a^	64.33 ± 0.26 ^b^
3	75.19 ± 0.34 ^c^	48.71 ± 0.16 ^c^	105.25 ± 0.32 ^c^	59.58 ± 0.18 ^c^	79.95 ± 0.24 ^bfe^	67.39 ± 0.31 ^c^
4	77.39 ± 0.30 ^d^	45.60 ± 0.17 ^d^	105.66 ± 0.34 ^c^	56.93 ± 0.21 ^d^	80.06 ± 0.33 ^bde^	70.91 ± 0.29 ^d^
5	76.15 ± 0.24 ^e^	30.85 ± 0.12 ^e^	115.49 ± 0.24 ^a^	46.31 ± 0.17 ^e^	82.11 ± 0.30 ^c^	58.20 ± 0.21 ^e^
6	73.86 ± 0.27 ^f^	31.06 ± 0.12 ^e^	117.07 ± 0.31 ^b^	43.67 ± 0.16 ^f^	82.45 ± 0.35 ^c^	53.57 ± 0.25 ^f^
7	77.22 ± 0.23 ^d^	40.51 ± 0.15 ^f^	105.86 ± 0.22 ^c^	51.11 ± 0.17 ^b^	81.14 ± 0.29 ^ae^	62.04 ± 0.21 ^g^
8	79.25 ± 0.28 ^g^	38.60 ± 0.19 ^g^	106.94 ± 0.29 ^d^	49.88 ± 0.20 ^g^	81.14 ± 0.36 ^ae^	63.48 ± 0.26 ^h^
9	69.81 ± 0.25 ^h^	39.71 ± 0.13 ^h^	109.49 ± 0.34 ^e^	53.12 ± 0.26 ^h^	80.37 ± 0.30 ^dfe^	66.48 ± 0.33 ^i^
10	77.47 ± 0.29 ^d^	35.21 ± 0.14 ^i^	110.96 ± 0.27 ^f^	50.27 ± 0.16 ^g^	81.25 ± 0.26 ^ae^	62.45 ± 0.30 ^g^
11	71.58 ± 0.35 ^i^	30.58 ± 0.10 ^e^	121.60 ± 0.24 ^g^	45.49 ± 0.18 ^i^	82.41 ± 0.34 ^c^	57.31 ± 0.26 ^j^
12	77.43 ± 0.38 ^d^	44.92 ± 0.22 ^j^	104.29 ± 0.31 ^h^	55.91 ± 0.20 ^j^	81.36 ± 0.20 ^a^	61.74 ± 0.26 ^g^
13	71.06 ± 0.29 ^j^	51.07 ± 0.25 ^k^	109.65 ± 0.28 ^e^	60.29 ± 0.23 ^k^	80.38 ± 0.24 ^e^	73.92 ± 0.37 ^k^
14	77.94 ± 0.32 ^bd^	33.24 ± 0.13 ^l^	110.66 ± 0.22 ^f^	46.02 ± 0.17 ^ei^	82.38 ± 0.30 ^c^	57.47 ± 0.20 ^ej^
15	75.44 ± 0.33 ^ce^	39.41 ± 0.14 ^h^	109.44 ± 0.26 ^e^	52.88 ± 0.21 ^h^	81.66 ± 0.29 ^a^	63.43 ± 0.20 ^h^

The values are presented as the arithmetic mean ± standard deviation of three measurements. Different superscript letters in the same column indicate statistically significant differences between the samples (*p* ≤ 0.05, Tukey test). Samples labelling: **R**—dispersions with raspberry anthocyanins; **C**—dispersions with chlorophylls; **B**—dispersions with β-carotene.

**Table 4:** The ratios of distilled water, lecithin solution, and CMC-Na solution in liposomal carrier systems obtained from 15 experimental runs by the Central Composite Design (CCD).

Carrier System No.	CMC-Na Solution 1 wt.% (mL)	Lecithin Solution 1 wt.% (mL)	Distilled Water (mL)
1	0.5	20.0	60.0
2	5.0	20.0	60.0
3	0.5	40.0	60.0
4	5.0	40.0	60.0
5	0.5	20.0	90.0
6	5.0	20.0	90.0
7	0.5	40.0	90.0
8	5.0	40.0	90.0
9	0.0	30.0	75.0
10	6.5	30.0	75.0
11	2.75	13.18	75.0
12	2.75	46.81	75.0
13	2.75	30.0	49.77
14	2.75	30.0	100.22
15	2.75	30.0	75.0

## Data Availability

The original contributions presented in the study are included in the article and the Supplementary Materials; further inquiries can be directed to the corresponding author.

## Supplementary Materials

The following supporting information can be downloaded at: https://www.mdpi.com/article/10.3390/molecules30081845/s1, Table S1: Rheological data of R dispersions; Table S2: Rheological data of C dispersions; Table S3: Rheological data of B dispersions.

## Abbreviations

The following abbreviations are used in this manuscript:

CCD	Central Composite Design
CMC-Na	Carboxymethylcellulose sodium salt
*EE*	Encapsulation efficiency
ICP-MS	Inductively coupled plasma-mass spectrometry
O/W	Oil-in-water emulsion
*PDI*	Polydispersity index
RPM	Rotations per minute
