# Supplementary material for: Study of Natural Dyes’ Liposomal Encapsulation in Food Dispersion Model Systems via High-Pressure Homogenization
Source: Molecules. 2025 Apr 20;30(8):1845. doi: 10.3390/molecules30081845 (PMC12029904; doi:10.3390/molecules30081845)
Supplement: Supplementary file 1 [file molecules-30-01845-s001.zip › Tables S1-S3. Rheological data (Supplementary material) (23.3.2025).pdf]

Supplementary Materials: Results of rheological measurements.

Table S1: Rheological data of **R** dispersions (for carrier systems No. 1–15).

1					2					3					4					5				
t (s)	γ (1/s)	τ (Pa)	η (Pa.s)	T (°C)	t (s)	γ (1/s)	τ (Pa)	η (Pa.s)	T (°C)	t (s)	γ (1/s)	τ (Pa)	η (Pa.s)	T (°C)	t (s)	γ (1/s)	τ (Pa)	η (Pa.s)	T (°C)	t (s)	γ (1/s)	τ (Pa)	η (Pa.s)	T (°C)
124.45	4.104	0.0808	0.0197	20.00	124.35	4.103	0.0761	0.0186	20.00	124.45	4.103	0.0729	0.0178	20.01	124.4	4.103	0.0754	0.0184	20.00	124.4	4.103	0.0726	0.0177	20.00
128.45	10.770	0.0803	0.0075	20.00	128.35	10.770	0.0861	0.0080	20.00	128.45	10.770	0.0790	0.0073	20.01	128.4	10.770	0.0845	0.0078	20.00	128.4	10.770	0.0783	0.0073	20.00
132.45	17.430	0.0881	0.0051	20.00	132.35	17.430	0.0966	0.0055	20.00	132.45	17.430	0.0856	0.0049	20.00	132.4	17.430	0.0946	0.0054	20.00	132.4	17.430	0.0850	0.0049	20.00
136.45	24.095	0.0953	0.0040	20.00	136.35	24.095	0.1077	0.0045	20.00	136.45	24.100	0.0937	0.0039	20.01	136.4	24.100	0.1050	0.0044	20.00	136.4	24.100	0.0911	0.0038	20.00
140.45	30.760	0.1025	0.0033	20.00	140.35	30.760	0.1194	0.0039	20.00	140.45	30.760	0.1009	0.0033	20.00	140.4	30.760	0.1155	0.0038	20.00	140.4	30.760	0.0990	0.0032	20.00
144.45	37.420	0.1113	0.0030	20.00	144.35	37.430	0.1308	0.0035	20.00	144.45	37.425	0.1096	0.0029	20.00	144.4	37.425	0.1259	0.0034	20.00	144.4	37.425	0.1057	0.0028	20.00
148.45	44.090	0.1167	0.0026	20.00	148.35	44.090	0.1424	0.0032	20.00	148.45	44.090	0.1155	0.0026	20.00	148.4	44.090	0.1373	0.0031	20.00	148.4	44.090	0.1144	0.0026	20.00
152.45	50.750	0.1243	0.0024	20.00	152.35	50.750	0.1550	0.0031	20.00	152.45	50.750	0.1235	0.0024	20.00	152.4	50.750	0.1474	0.0029	20.00	152.4	50.750	0.1232	0.0024	20.00
156.45	57.420	0.1327	0.0023	20.00	156.35	57.420	0.1668	0.0029	20.00	156.45	57.415	0.1316	0.0023	20.00	156.4	57.415	0.1592	0.0028	20.00	156.4	57.420	0.1293	0.0023	20.00
160.45	64.085	0.1424	0.0022	20.00	160.35	64.080	0.1791	0.0028	20.00	160.45	64.085	0.1420	0.0022	20.00	160.4	64.080	0.1705	0.0027	20.00	160.4	64.080	0.1374	0.0021	20.00
164.45	70.745	0.1509	0.0021	20.00	164.35	70.745	0.1911	0.0027	20.00	164.45	70.750	0.1506	0.0021	20.00	164.4	70.745	0.1821	0.0026	20.00	164.4	70.740	0.1433	0.0020	20.00
168.45	77.410	0.1591	0.0021	20.00	168.35	77.410	0.2043	0.0026	20.00	168.45	77.410	0.1591	0.0021	20.00	168.4	77.410	0.1931	0.0025	20.00	168.4	77.410	0.1558	0.0020	20.00
172.45	84.070	0.1704	0.0020	20.00	172.35	84.075	0.2173	0.0026	20.00	172.45	84.070	0.1706	0.0020	20.00	172.4	84.070	0.2045	0.0024	20.00	172.4	84.070	0.1628	0.0019	20.00
176.45	90.740	0.1757	0.0019	20.00	176.35	90.740	0.2302	0.0025	20.00	176.45	90.740	0.1753	0.0019	20.00	176.4	90.740	0.2186	0.0024	20.00	176.4	90.740	0.1726	0.0019	20.00
180.45	97.400	0.1879	0.0019	20.00	180.35	97.400	0.2426	0.0025	20.00	180.45	97.405	0.1878	0.0019	20.00	180.4	97.405	0.2302	0.0024	20.00	180.4	97.395	0.1794	0.0018	20.00
184.45	104.100	0.1968	0.0019	20.00	184.35	104.100	0.2555	0.0025	20.00	184.45	104.100	0.1963	0.0019	20.00	184.4	104.100	0.2440	0.0023	20.00	184.4	104.100	0.1892	0.0018	20.00
188.45	110.700	0.2155	0.0019	20.00	188.35	110.700	0.2701	0.0024	20.00	188.45	110.700	0.2123	0.0019	20.00	188.4	110.700	0.2542	0.0023	20.00	188.4	110.700	0.2210	0.0020	20.00
192.45	117.400	0.2504	0.0021	20.00	192.35	117.400	0.2833	0.0024	20.00	192.45	117.400	0.2437	0.0021	20.00	192.4	117.400	0.2646	0.0023	20.00	192.4	117.400	0.2436	0.0021	20.00
196.45	124.100	0.2683	0.0022	20.00	196.35	124.100	0.2975	0.0024	20.00	196.45	124.100	0.2643	0.0021	20.00	196.4	124.100	0.2796	0.0023	20.00	196.4	124.100	0.2694	0.0022	20.00
200.45	130.700	0.2863	0.0022	20.00	200.35	130.700	0.3111	0.0024	20.00	200.45	130.700	0.2912	0.0022	20.00	200.4	130.700	0.2930	0.0022	20.00	200.4	130.700	0.2737	0.0021	20.00
204.45	137.400	0.2967	0.0022	20.00	204.35	137.400	0.3251	0.0024	20.00	204.45	137.400	0.2997	0.0022	20.00	204.4	137.400	0.3078	0.0022	20.00	204.4	137.400	0.2895	0.0021	20.00
208.45	144.000	0.3210	0.0022	20.00	208.35	144.100	0.3404	0.0024	20.00	208.45	144.050	0.3189	0.0022	20.00	208.4	144.050	0.3201	0.0022	20.00	208.4	144.100	0.3118	0.0022	20.00
212.45	150.700	0.3468	0.0023	20.00	212.35	150.700	0.3551	0.0024	20.00	212.45	150.700	0.3387	0.0022	20.00	212.4	150.700	0.3424	0.0023	20.00	212.4	150.700	0.3293	0.0022	20.00
216.45	157.400	0.3598	0.0023	20.00	216.35	157.400	0.3718	0.0024	20.00	216.45	157.400	0.3556	0.0023	20.00	216.4	157.400	0.3815	0.0024	20.00	216.4	157.400	0.3537	0.0022	20.00
220.45	164.000	0.3814	0.0023	20.00	220.35	164.000	0.3997	0.0024	20.00	220.45	164.000	0.3812	0.0023	20.00	220.4	164.000	0.4155	0.0025	20.00	220.4	164.000	0.3658	0.0022	20.00
224.45	170.700	0.4000	0.0023	20.00	224.35	170.700	0.4424	0.0026	20.00	224.45	170.700	0.4004	0.0023	20.00	224.4	170.700	0.4484	0.0026	20.00	224.4	170.700	0.3817	0.0022	20.00
228.45	177.400	0.4218	0.0024	20.00	228.35	177.400	0.4800	0.0027	20.00	228.45	177.400	0.4268	0.0024	20.00	228.4	177.400	0.4698	0.0026	20.00	228.4	177.400	0.4068	0.0023	20.00
232.45	184.000	0.4445	0.0024	20.00	232.35	184.000	0.5159	0.0028	20.00	232.45	184.000	0.4463	0.0024	20.00	232.4	184.000	0.4987	0.0027	20.00	232.4	184.000	0.4268	0.0023	20.00
236.45	190.700	0.4579	0.0024	20.00	236.35	190.700	0.5373	0.0028	20.00	236.45	190.700	0.4640	0.0024	20.00	236.4	190.700	0.5299	0.0028	20.00	236.4	190.700	0.4517	0.0024	20.00
240.45	197.400	0.4752	0.0024	20.00	240.35	197.400	0.5640	0.0029	20.00	240.45	197.400	0.4897	0.0025	20.00	240.4	197.400	0.5512	0.0028	20.00	240.4	197.400	0.4704	0.0024	20.00
241.5	199.900	5.5410	0.0277	20.00	241.35	199.950	5.9600	0.0298	20.00	241.45	199.950	14.7100	0.0736	20.00	241.45	199.950	9.1822	0.0459	20.00	241.4	200.000	17.7550	0.0888	20.00
245.55	195.700	0.3278	0.0017	20.00	245.45	195.700	0.4136	0.0021	20.00	245.5	195.700	0.3438	0.0018	20.00	245.45	195.700	0.4018	0.0021	20.00	245.4	195.700	0.3258	0.0017	20.00
249.55	189.100	0.3101	0.0016	20.00	249.45	189.050	0.3837	0.0020	20.00	249.5	189.000	0.3254	0.0017	20.00	249.45	189.000	0.3687	0.0020	20.00	249.4	189.100	0.2998	0.0016	20.00
253.55	182.650	0.2942	0.0016	20.00	253.45	182.400	0.3583	0.0020	20.00	253.5	182.400	0.2993	0.0016	20.00	253.45	182.400	0.3414	0.0019	20.00	253.4	182.700	0.2888	0.0016	20.00
257.55	176.100	0.2675	0.0015	20.00	257.45	175.700	0.3276	0.0019	20.00	257.5	176.100	0.2777	0.0016	20.00	257.45	175.700	0.3156	0.0018	20.00	257.4	176.100	0.2617	0.0015	20.00
261.55	169.400	0.2422	0.0014	20.00	261.45	169.100	0.2975	0.0018	20.00	261.5	169.400	0.2502	0.0015	20.00	261.45	169.150	0.2976	0.0018	20.00	261.4	169.400	0.2449	0.0014	20.00
265.55	162.700	0.2251	0.0014	20.00	265.45	162.700	0.2642	0.0016	20.00	265.5	162.700	0.2304	0.0014	20.00	265.45	162.700	0.2650	0.0016	20.00	265.4	162.700	0.2247	0.0014	20.00
269.55	156.100	0.2031	0.0013	20.00	269.45	156.100	0.2319	0.0015	20.00	269.5	156.100	0.2087	0.0013	20.00	269.45	156.100	0.2386	0.0015	20.00	269.4	156.100	0.2020	0.0013	20.00
273.55	149.400	0.1861	0.0012	20.00	273.45	149.400	0.2107	0.0014	20.00	273.5	149.400	0.1944	0.0013	20.00	273.45	149.400	0.2077	0.0014	20.00	273.4	149.400	0.1810	0.0012	20.00
277.55	142.700	0.1661	0.0012	20.00	277.45	142.700	0.1946	0.0014	20.00	277.5	142.700	0.1709	0.0012	20.00	277.45	142.700	0.1826	0.0013	20.00	277.4	142.700	0.1599	0.0011	20.00
281.55	136.100	0.1451	0.0011	20.00	281.45	136.100	0.1828	0.0013	20.00	281.5	136.100	0.1540	0.0011	20.00	281.45	136.100	0.1626	0.0012	20.00	281.4	136.100	0.1443	0.0011	20.00
285.55	129.400	0.1271	0.0010	20.00	285.45	129.400	0.1675	0.0013	20.00	285.5	129.400	0.1353	0.0010	20.00	285.45	129.400	0.1502	0.0012	20.00	285.4	129.400	0.1264	0.0010	20.00
289.55	122.750	0.1069	0.0009	20.00	289.45	122.750	0.1553	0.0013	20.00	289.5	122.700	0.1137	0.0009	20.00	289.45	122.700	0.1367	0.0011	20.00	289.4	122.700	0.1119	0.0009	20.00
293.55	116.100	0.0893	0.0008	20.00	293.45	116.100	0.1391	0.0012	20.00	293.5	116.100	0.0909	0.0008	20.00	293.45	116.100	0.1256	0.0011	20.00	293.4	116.100	0.0917	0.0008	20.00
297.55	109.400	0.0680	0.0006	20.00	297.45	109.400	0.1272	0.0012	20.00	297.5	109.400	0.0712	0.0007	20.00	297.45	109.400	0.1114	0.0010	20.00	297.4	109.400	0.0773	0.0007	20.00
301.55	102.800	0.0545	0.0005	20.00	301.45	102.750	0.1126	0.0011	20.00	301.5	102.750	0.0583	0.0006	20.00	301.45	102.750	0.0987	0.0010	20.00	301.4	102.750	0.0533	0.0005	20.00
305.55	96.125	0.0441	0.0005	20.00	305.45	96.090	0.1007	0.0010	20.00	305.5	96.090	0.0466	0.0005	20.00	305.45	96.090	0.0864	0.0009	20.00	305.4	96.155	0.0501	0.0005	20.00
309.55	89.925	0.0340	0.0004	20.00	309.45	89.425	0.0873	0.0010	20.00	309.5	89.680	0.0508	0.0006	20.00	309.45	89.425	0.0734	0.0008	20.00	309.4	89.920	0.0288	0.0003	20.00
313.55	83.260	0.0234	0.0003	20.00	313.45	82.765	0.0757	0.0009	20.00	313.5	83.250	0.0303	0.0004	20.00	313.45	82.760	0.0610	0.0007	20.00	313.4	83.250	0.0239	0.0003	20.00
317.55	76.595	0.0188	0.0002	20.00	317.45	76.095	0.0619	0.0008	20.00	317.5	76.590	0.0224	0.0003	20.00	317.45	76.095	0.0497	0.0007	20.00	317.4	76.635	0.0145	0.0002	20.00

321.55	70.100	0.0081	0.0001	20.00	321.45	69.430	0.0495	0.0007	20.00	321.5	70.020	0.0119	0.0002	20.00	321.45	69.435	0.0392	0.0006	20.00	321.4	70.115	0.0091	0.0001	20.00
325.55	63.660	0.0047	0.0001	20.00	325.45	62.780	0.0414	0.0007	20.00	325.5	63.535	0.0090	0.0001	20.00	325.45	63.275	0.0289	0.0005	20.00	325.4	63.695	0.0021	0.0000	20.00
329.55	57.035	0.0078	0.0001	20.00	329.45	56.610	0.0262	0.0005	20.00	329.5	57.025	0.0056	0.0001	20.00	329.45	56.600	0.0182	0.0003	20.00	329.4	57.030	0.0106	0.0002	20.00
333.55	50.375	0.0141	0.0003	20.00	333.45	49.935	0.0136	0.0003	20.00	333.5	50.360	0.0111	0.0002	20.00	333.45	50.090	0.0112	0.0002	20.00	333.4	50.365	0.0184	0.0004	20.00
337.55	43.255	0.0302	0.0007	20.00	337.45	43.535	0.0082	0.0002	20.00	337.5	43.675	0.0268	0.0006	20.00	337.45	43.710	0.0023	0.0001	20.00	337.4	43.190	0.0278	0.0006	20.00
341.55	36.545	0.0320	0.0009	20.00	341.45	37.040	0.0077	0.0002	20.00	341.5	36.540	0.0296	0.0008	20.00	341.45	37.035	0.0134	0.0004	20.00	341.4	36.545	0.0321	0.0009	20.00
345.55	29.885	0.0388	0.0013	20.00	345.45	30.375	0.0193	0.0006	20.00	345.5	29.870	0.0372	0.0012	20.00	345.45	30.370	0.0262	0.0009	20.00	345.4	29.880	0.0400	0.0013	20.00
349.55	23.220	0.0467	0.0020	20.00	349.45	23.255	0.0368	0.0016	20.00	349.5	23.215	0.0454	0.0020	20.00	349.45	23.210	0.0346	0.0015	20.00	349.4	23.215	0.0461	0.0020	20.00
353.55	16.555	0.0528	0.0032	20.00	353.45	16.555	0.0422	0.0026	20.00	353.5	16.550	0.0522	0.0032	20.00	353.45	16.550	0.0446	0.0027	20.00	353.4	16.550	0.0535	0.0032	20.00
357.55	9.891	0.0595	0.0060	20.00	357.45	9.888	0.0528	0.0053	20.00	357.5	9.884	0.0589	0.0060	20.00	357.45	9.888	0.0546	0.0055	20.00	357.4	9.887	0.0594	0.0060	20.00
361.55	3.228	0.0656	0.0203	20.00	361.45	3.223	0.0633	0.0197	20.00	361.5	3.220	0.0654	0.0203	20.00	361.45	3.223	0.0640	0.0199	20.00	361.4	3.222	0.0656	0.0204	20.00
6					7					8					9					10				
t (s)	γ (1/s)	τ (Pa)	η (Pa.s)	T (°C)	t (s)	γ (1/s)	τ (Pa)	η (Pa.s)	T (°C)	t (s)	γ (1/s)	τ (Pa)	η (Pa.s)	T (°C)	t (s)	γ (1/s)	τ (Pa)	η (Pa.s)	T (°C)	t (s)	γ (1/s)	τ (Pa)	η (Pa.s)	T (°C)
124.45	4.103	0.0739	0.0180	20.00	124.4	4.103	0.0724	0.0177	19.99	124.5	4.103	0.0739	0.0180	20.00	124.5	4.102	0.0725	0.0177	20.00	124.4	4.101	0.0797	0.0194	20.00
128.45	10.770	0.0821	0.0076	20.00	128.4	10.770	0.0785	0.0073	20.00	128.5	10.770	0.0823	0.0076	20.00	128.5	10.770	0.0780	0.0072	20.00	128.4	10.770	0.0878	0.0082	20.00
132.45	17.430	0.0912	0.0052	20.00	132.4	17.430	0.0847	0.0049	20.00	132.5	17.430	0.0916	0.0053	20.00	132.5	17.430	0.0841	0.0048	20.00	132.4	17.430	0.0999	0.0057	20.00
136.45	24.095	0.0999	0.0041	20.00	136.4	24.095	0.0929	0.0039	20.00	136.5	24.100	0.1003	0.0042	20.00	136.5	24.090	0.0920	0.0038	20.00	136.4	24.100	0.1111	0.0046	20.00
140.45	30.760	0.1100	0.0036	20.00	140.4	30.760	0.0991	0.0032	20.00	140.5	30.760	0.1104	0.0036	20.00	140.5	30.760	0.0982	0.0032	20.00	140.4	30.760	0.1222	0.0040	20.00
144.45	37.425	0.1194	0.0032	20.00	144.4	37.420	0.1076	0.0029	19.99	144.5	37.425	0.1198	0.0032	20.00	144.5	37.425	0.1067	0.0029	20.00	144.4	37.425	0.1343	0.0036	20.00
148.45	44.090	0.1306	0.0030	20.00	148.4	44.090	0.1128	0.0026	19.99	148.5	44.090	0.1311	0.0030	20.00	148.5	44.090	0.1113	0.0025	20.00	148.4	44.090	0.1474	0.0033	20.00
152.45	50.750	0.1418	0.0028	20.00	152.4	50.750	0.1200	0.0024	19.99	152.5	50.750	0.1418	0.0028	20.00	152.5	50.750	0.1191	0.0023	20.00	152.4	50.750	0.1597	0.0031	20.00
156.45	57.420	0.1498	0.0026	20.00	156.4	57.415	0.1295	0.0023	19.99	156.5	57.415	0.1508	0.0026	20.00	156.5	57.420	0.1274	0.0022	20.00	156.4	57.415	0.1718	0.0030	20.00
160.45	64.080	0.1611	0.0025	20.00	160.4	64.085	0.1384	0.0022	19.99	160.5	64.080	0.1612	0.0025	20.00	160.5	64.085	0.1367	0.0021	20.00	160.4	64.080	0.1853	0.0029	20.00
164.45	70.745	0.1698	0.0024	20.00	164.4	70.750	0.1480	0.0021	19.99	164.5	70.740	0.1712	0.0024	20.00	164.5	70.750	0.1460	0.0021	20.00	164.4	70.745	0.1981	0.0028	20.00
168.45	77.410	0.1847	0.0024	20.00	168.4	77.410	0.1531	0.0020	20.00	168.5	77.410	0.1840	0.0024	20.00	168.5	77.410	0.1527	0.0020	20.00	168.4	77.410	0.2119	0.0027	20.00
172.45	84.070	0.1940	0.0023	20.00	172.4	84.075	0.1642	0.0020	20.00	172.5	84.070	0.1938	0.0023	20.00	172.5	84.070	0.1642	0.0020	20.00	172.4	84.075	0.2247	0.0027	20.00
176.45	90.740	0.2059	0.0023	20.00	176.4	90.740	0.1703	0.0019	20.00	176.5	90.735	0.2067	0.0023	20.00	176.5	90.740	0.1681	0.0019	20.00	176.4	90.740	0.2388	0.0026	20.00
180.45	97.400	0.2153	0.0022	20.00	180.4	97.405	0.1827	0.0019	20.00	180.5	97.400	0.2164	0.0022	20.00	180.5	97.400	0.1806	0.0019	20.00	180.4	97.400	0.2526	0.0026	20.00
184.45	104.100	0.2258	0.0022	20.00	184.4	104.100	0.1933	0.0019	20.00	184.5	104.100	0.2276	0.0022	20.00	184.5	104.100	0.1911	0.0018	20.00	184.4	104.100	0.2667	0.0026	20.00
188.45	110.700	0.2405	0.0022	20.00	188.4	110.700	0.2158	0.0019	20.00	188.5	110.700	0.2399	0.0022	20.00	188.5	110.700	0.2216	0.0020	20.00	188.4	110.700	0.2775	0.0025	20.00
192.45	117.400	0.2505	0.0021	20.00	192.4	117.400	0.2473	0.0021	20.00	192.5	117.400	0.2507	0.0021	20.00	192.5	117.400	0.2488	0.0021	20.00	192.4	117.400	0.2887	0.0025	20.00
196.45	124.100	0.2654	0.0021	20.00	196.4	124.100	0.2657	0.0021	20.00	196.5	124.100	0.2657	0.0021	20.00	196.5	124.100	0.2617	0.0021	20.00	196.4	124.100	0.3041	0.0025	20.00
200.45	130.700	0.2756	0.0021	20.00	200.4	130.700	0.2776	0.0021	20.00	200.5	130.700	0.2765	0.0021	20.00	200.5	130.700	0.2714	0.0021	20.00	200.4	130.700	0.3188	0.0024	20.00
204.45	137.400	0.2894	0.0021	20.00	204.4	137.400	0.2932	0.0021	20.00	204.5	137.400	0.2904	0.0021	20.00	204.5	137.400	0.2875	0.0021	20.00	204.4	137.400	0.3341	0.0024	20.00
208.45	144.050	0.3271	0.0023	20.00	208.4	144.050	0.3111	0.0022	20.00	208.5	144.050	0.3212	0.0022	20.00	208.5	144.100	0.3105	0.0022	20.00	208.4	144.050	0.3491	0.0024	20.00
212.45	150.700	0.3640	0.0024	20.00	212.4	150.700	0.3338	0.0022	20.00	212.5	150.700	0.3562	0.0024	20.00	212.5	150.700	0.3321	0.0022	20.00	212.4	150.700	0.3650	0.0024	20.00
216.45	157.400	0.3982	0.0025	20.00	216.4	157.400	0.3463	0.0022	20.00	216.5	157.400	0.3903	0.0025	20.00	216.5	157.400	0.3482	0.0022	20.00	216.4	157.400	0.3812	0.0024	20.00
220.45	164.000	0.4091	0.0025	20.00	220.4	164.000	0.3727	0.0023	20.00	220.5	164.000	0.4097	0.0025	20.00	220.5	164.000	0.3686	0.0022	20.00	220.4	164.000	0.4234	0.0026	20.00
224.45	170.700	0.4254	0.0025	20.00	224.4	170.700	0.3947	0.0023	20.00	224.5	170.700	0.4307	0.0025	20.00	224.5	170.700	0.3871	0.0023	20.00	224.4	170.700	0.4631	0.0027	20.00
228.45	177.400	0.4535	0.0026	20.00	228.4	177.400	0.4144	0.0023	20.00	228.5	177.400	0.4513	0.0025	20.00	228.5	177.400	0.4052	0.0023	20.00	228.4	177.400	0.5000	0.0028	20.00
232.45	184.000	0.4754	0.0026	20.00	232.4	184.000	0.4380	0.0024	20.00	232.5	184.000	0.4760	0.0026	20.00	232.5	184.000	0.4287	0.0023	20.00	232.4	184.000	0.5374	0.0029	20.00
236.45	190.700	0.5035	0.0026	20.00	236.4	190.700	0.4528	0.0024	20.00	236.5	190.700	0.5036	0.0026	20.00	236.5	190.700	0.4425	0.0023	20.00	236.4	190.700	0.5512	0.0029	20.00
240.45	197.400	0.5281	0.0027	20.00	240.4	197.400	0.4777	0.0024	20.00	240.5	197.400	0.5255	0.0027	20.00	240.5	197.400	0.4684	0.0024	20.00	240.4	197.400	0.5705	0.0029	20.00
241.5	199.950	15.1450	0.0758	20.00	241.4	199.900	12.1015	0.0605	20.00	241.55	199.900	14.2400	0.0712	20.00	241.5	199.900	15.5750	0.0779	20.00	241.4	199.950	17.8700	0.0894	20.00
245.55	195.700	0.3801	0.0019	20.00	245.5	195.700	0.3299	0.0017	20.00	245.55	195.700	0.3781	0.0019	20.00	245.6	195.700	0.3223	0.0016	20.00	245.4	195.700	0.4145	0.0021	20.00
249.55	189.100	0.3502	0.0019	20.00	249.5	189.050	0.3117	0.0016	20.00	249.55	189.100	0.3461	0.0018	20.00	249.55	189.050	0.3056	0.0016	20.00	249.4	189.050	0.3846	0.0020	20.00
253.55	182.400	0.3289	0.0018	20.00	253.5	182.650	0.2928	0.0016	20.00	253.55	182.400	0.3266	0.0018	20.00	253.55	182.700	0.2810	0.0015	20.00	253.4	182.400	0.3597	0.0020	20.00
257.55	175.700	0.3051	0.0017	20.00	257.5	176.100	0.2694	0.0015	20.00	257.55	175.700	0.2956	0.0017	20.00	257.55	176.100	0.2624	0.0015	20.00	257.4	175.700	0.3333	0.0019	20.00
261.55	169.400	0.2793	0.0016	20.00	261.5	169.400	0.2436	0.0014	20.00	261.55	169.400	0.2767	0.0016	20.00	261.6	169.400	0.2330	0.0014	20.00	261.4	169.100	0.3061	0.0018	20.00
265.55	162.700	0.2559	0.0016	20.00	265.5	162.700	0.2241	0.0014	20.00	265.55	162.700	0.2507	0.0015	20.00	265.55	162.700	0.2150	0.0013	20.00	265.4	162.700	0.2731	0.0017	20.00
269.55	156.100	0.2318	0.0015	20.00	269.5	156.100	0.2049	0.0013	20.00	269.55	156.100	0.2289	0.0015	20.00	269.55	156.100	0.1941	0.0012	20.00	269.4	156.100	0.2393	0.0015	20.00
273.55	149.400	0.2077	0.0014	20.00	273.5	149.400	0.1882	0.0013	20.00	273.55	149.400	0.2020	0.0014	20.00	273.55	149.400	0.1809	0.0012	20.00	273.4	149.400	0.2104	0.0014	20.00
277.55	142.700	0.1815	0.0013	20.00	277.5	142.700	0.1667	0.0012	20.00	277.55	142.700	0.1774	0.0012	20.00	277.6	142.700	0.1605	0.0011	20.00	277.4	142.700	0.1942	0.0014	20.00
281.55	136.100	0.1576	0.0012	20.00	281.5	136.100	0.1441	0.0011	20.00	281.55	136.100	0.1531	0.0011	20.00	281.55	136.100	0.1409	0.0010	20.00	281.4	136.100	0.1796	0.0013	20.00
285.55	129.400	0.1331	0.0010	20.00	285.5	129.400	0.1279	0.0010	20.00	285.55	129.400	0.1332	0.0010	20.00	285.55	129.400	0.1239	0.0010	20.00	285.4	129.400	0.1655	0.0013	20.00
289.55	122.700	0.1221	0.0010	20.00	289.5	122.700	0.1066	0.0009	20.00	289.55	122.750	0.1232	0.0010	20.00	289.55	122.800	0.1030	0.0008	20.00	289.4	122.700	0.1518	0.0012	20.00
293.55	116.100	0.1067	0.0009	20.00	293.5	116.100	0.0905	0.0008	20.00	293.55	116.100	0.1100	0.0009	20.00	293.6	116.100	0.0877	0.0008	20.00	293.4	116.100	0.1386	0.0012	20.00
297.55	109.400	0.0973	0.0009	20.00	297.5	109.400	0.0688	0.0006	20.00	297.55	109.400	0.0995	0.0009	20.00	297.6	109.400	0.0688	0.0006	20.00	297.4	109.400	0.1256	0.0011	20.00
301.55	102.750	0.0831	0.0008	20.00	301.5	102.750	0.0557	0.0005	20.00	301.55	102.800	0.0845	0.0008	20.00	301.55	102.800	0.0568	0.0006	20.00	301.4	102.750	0.1112	0.0011	20.00

305.55	96.090	0.0735	0.0008	20.00	305.5	96.085	0.0403	0.0004	20.00	305.55	96.095	0.0748	0.0008	20.00	305.55	96.240	0.0477	0.0005	20.00	305.4	96.085	0.0982	0.0010	20.00
309.55	89.425	0.0614	0.0007	20.00	309.5	89.920	0.0343	0.0004	20.00	309.55	89.430	0.0612	0.0007	20.00	309.55	89.925	0.0322	0.0004	20.00	309.4	89.425	0.0847	0.0009	20.00
313.55	82.765	0.0532	0.0006	20.00	313.5	83.255	0.0221	0.0003	20.00	313.55	82.765	0.0529	0.0006	20.00	313.6	83.265	0.0203	0.0002	20.00	313.4	82.760	0.0726	0.0009	20.00
317.55	76.095	0.0400	0.0005	20.00	317.5	76.590	0.0187	0.0002	20.00	317.55	76.105	0.0397	0.0005	20.00	317.55	76.665	0.0186	0.0002	20.00	317.4	76.100	0.0599	0.0008	20.00
321.55	69.945	0.0305	0.0004	20.00	321.5	70.115	0.0071	0.0001	20.00	321.55	69.925	0.0366	0.0005	20.00	321.55	70.095	0.0053	0.0001	20.00	321.4	69.435	0.0483	0.0007	20.00
325.55	63.265	0.0198	0.0003	20.00	325.5	63.675	0.0033	0.0001	20.00	325.55	63.270	0.0204	0.0003	20.00	325.55	63.710	0.0022	0.0000	20.00	325.4	62.765	0.0354	0.0006	20.00
329.55	56.660	0.0119	0.0002	20.00	329.5	57.025	0.0074	0.0001	20.00	329.55	56.650	0.0134	0.0002	20.00	329.6	57.035	0.0095	0.0002	20.00	329.4	56.605	0.0248	0.0004	20.00
333.55	50.375	0.0006	0.0000	20.00	333.5	50.365	0.0140	0.0003	20.00	333.55	50.380	0.0030	0.0001	20.00	333.55	50.375	0.0153	0.0003	20.00	333.4	49.940	0.0133	0.0003	20.00
337.55	43.705	0.0098	0.0002	20.00	337.5	43.230	0.0295	0.0007	20.00	337.55	43.710	0.0087	0.0002	20.00	337.55	43.210	0.0234	0.0005	20.00	337.4	43.605	0.0071	0.0002	20.00
341.55	37.045	0.0186	0.0005	20.00	341.5	36.540	0.0327	0.0009	20.00	341.55	37.045	0.0185	0.0005	20.00	341.55	36.545	0.0338	0.0009	20.00	341.4	37.040	0.0086	0.0002	20.00
345.55	29.875	0.0314	0.0011	20.00	345.5	29.875	0.0389	0.0013	20.00	345.55	29.890	0.0328	0.0011	20.00	345.6	29.885	0.0399	0.0013	20.00	345.4	30.375	0.0200	0.0007	20.00
349.55	23.220	0.0381	0.0016	20.00	349.5	23.215	0.0472	0.0020	20.00	349.55	23.220	0.0378	0.0016	20.00	349.6	23.225	0.0480	0.0021	20.00	349.4	23.215	0.0346	0.0015	20.00
353.55	16.555	0.0476	0.0029	20.00	353.5	16.550	0.0529	0.0032	20.00	353.55	16.555	0.0471	0.0028	20.00	353.55	16.555	0.0535	0.0032	20.00	353.4	16.550	0.0428	0.0026	20.00
357.55	9.891	0.0562	0.0057	20.00	357.5	9.885	0.0599	0.0061	20.00	357.55	9.893	0.0559	0.0057	20.00	357.55	9.895	0.0602	0.0061	20.00	357.4	9.887	0.0535	0.0054	20.00
361.55	3.226	0.0648	0.0201	20.00	361.5	3.221	0.0659	0.0205	20.00	361.55	3.229	0.0648	0.0201	20.00	361.55	3.231	0.0661	0.0205	20.00	361.4	3.223	0.0640	0.0198	20.00
11					12					13					14					15				
t (s)	*γ* (1/s)	*τ* (Pa)	*η* (Pa.s)	*T* (°C)	*t* (s)	*γ* (1/s)	*τ* (Pa)	*η* (Pa.s)	*T* (°C)	*t* (s)	*γ* (1/s)	*τ* (Pa)	*η* (Pa.s)	*T* (°C)	*t* (s)	*γ* (1/s)	*τ* (Pa)	*η* (Pa.s)	*T* (°C)	*t* (s)	*γ* (1/s)	*τ* (Pa)	*η* (Pa.s)	*T* (°C)
124.5	4.102	0.0762	0.0186	19.99	124.45	4.102	0.0772	0.0188	20.00	124.4	4.103	0.0769	0.0188	20.00	130.9	4.103	0.0748	0.0182	20.01	124.4	4.103	0.0751	0.0183	20.00
128.5	10.770	0.0867	0.0081	19.99	128.45	10.770	0.0864	0.0080	20.00	128.4	10.770	0.0886	0.0082	20.00	134.9	10.770	0.0833	0.0077	20.01	128.4	10.770	0.0845	0.0078	20.00
132.5	17.430	0.0976	0.0056	19.99	132.45	17.430	0.0969	0.0056	20.00	132.4	17.430	0.1009	0.0058	20.00	138.9	17.430	0.0924	0.0053	20.01	132.4	17.430	0.0942	0.0054	20.00
136.5	24.100	0.1090	0.0045	19.99	136.45	24.100	0.1072	0.0044	20.00	136.4	24.100	0.1123	0.0047	20.00	142.9	24.100	0.1022	0.0042	20.01	136.4	24.100	0.1042	0.0043	20.01
140.5	30.760	0.1204	0.0039	19.99	140.45	30.760	0.1178	0.0038	20.00	140.4	30.760	0.1250	0.0041	20.00	146.9	30.760	0.1118	0.0036	20.01	140.4	30.760	0.1145	0.0037	20.01
144.5	37.425	0.1321	0.0035	19.99	144.45	37.420	0.1285	0.0034	20.00	144.4	37.425	0.1367	0.0037	20.00	150.9	37.425	0.1222	0.0033	20.01	144.4	37.425	0.1246	0.0033	20.01
148.5	44.090	0.1436	0.0033	20.00	148.45	44.090	0.1398	0.0032	20.00	148.4	44.090	0.1505	0.0034	20.00	154.9	44.090	0.1311	0.0030	20.01	148.4	44.090	0.1354	0.0031	20.01
152.5	50.750	0.1552	0.0031	20.00	152.45	50.750	0.1492	0.0029	20.00	152.4	50.750	0.1629	0.0032	20.00	158.9	50.750	0.1427	0.0028	20.01	152.4	50.750	0.1458	0.0029	20.00
156.5	57.415	0.1678	0.0029	20.00	156.45	57.420	0.1623	0.0028	20.00	156.4	57.415	0.1748	0.0030	20.00	162.9	57.420	0.1510	0.0026	20.01	156.4	57.418	0.1572	0.0027	20.00
160.5	64.080	0.1801	0.0028	20.00	160.45	64.085	0.1731	0.0027	20.00	160.4	64.080	0.1868	0.0029	20.00	166.9	64.085	0.1632	0.0025	20.01	160.4	64.083	0.1677	0.0026	20.00
164.5	70.745	0.1924	0.0027	20.00	164.45	70.745	0.1850	0.0026	20.00	164.4	70.740	0.1986	0.0028	20.00	170.9	70.745	0.1730	0.0024	20.01	164.4	70.743	0.1792	0.0025	20.00
168.5	77.410	0.2046	0.0026	20.00	168.45	77.410	0.1958	0.0025	20.00	168.4	77.410	0.2135	0.0028	20.00	174.9	77.410	0.1855	0.0024	20.01	168.4	77.410	0.1901	0.0025	20.00
172.5	84.070	0.2176	0.0026	20.00	172.45	84.075	0.2056	0.0024	20.00	172.4	84.070	0.2246	0.0027	20.00	178.9	84.070	0.1979	0.0024	20.01	172.4	84.073	0.2015	0.0024	20.00
176.5	90.740	0.2293	0.0025	20.00	176.45	90.735	0.2196	0.0024	20.00	176.4	90.740	0.2404	0.0026	20.00	182.9	90.740	0.2063	0.0023	20.01	176.4	90.738	0.2139	0.0024	20.00
180.5	97.405	0.2431	0.0025	20.00	180.45	97.400	0.2316	0.0024	20.00	180.4	97.405	0.2515	0.0026	20.00	186.9	97.395	0.2186	0.0022	20.01	180.4	97.402	0.2255	0.0023	20.00
184.5	104.100	0.2561	0.0025	20.00	184.45	104.100	0.2454	0.0024	20.00	184.4	104.100	0.2654	0.0026	20.00	190.9	104.100	0.2286	0.0022	20.00	184.4	104.100	0.2382	0.0023	20.00
188.5	110.700	0.2697	0.0024	20.00	188.45	110.700	0.2559	0.0023	20.00	188.4	110.700	0.2790	0.0025	20.00	194.9	110.700	0.2440	0.0022	20.00	188.4	110.700	0.2496	0.0023	20.00
192.5	117.400	0.2840	0.0024	20.00	192.45	117.400	0.2667	0.0023	20.00	192.4	117.400	0.2914	0.0025	20.00	198.9	117.400	0.2560	0.0022	20.00	192.4	117.400	0.2622	0.0022	20.00
196.5	124.100	0.2958	0.0024	20.00	196.45	124.100	0.2811	0.0023	20.00	196.4	124.100	0.3091	0.0025	20.00	202.9	124.100	0.2662	0.0021	20.00	196.4	124.100	0.2753	0.0022	20.00
200.5	130.700	0.3110	0.0024	20.00	200.45	130.700	0.2946	0.0023	20.00	200.4	130.700	0.3216	0.0025	20.00	206.9	130.700	0.2788	0.0021	20.00	200.4	130.700	0.2886	0.0022	20.00
204.5	137.400	0.3251	0.0024	20.00	204.45	137.400	0.3097	0.0023	20.00	204.4	137.400	0.3364	0.0024	20.00	210.9	137.400	0.2928	0.0021	20.00	204.4	137.400	0.3031	0.0022	20.00
208.5	144.050	0.3402	0.0024	20.00	208.45	144.100	0.3207	0.0022	20.00	208.4	144.050	0.3511	0.0024	20.00	214.9	144.100	0.3342	0.0023	20.00	208.4	144.067	0.3217	0.0022	20.00
212.5	150.700	0.3565	0.0024	20.00	212.45	150.700	0.3482	0.0023	20.00	212.4	150.700	0.3646	0.0024	20.00	218.9	150.700	0.3655	0.0024	20.00	212.4	150.700	0.3596	0.0024	20.00
216.5	157.400	0.3758	0.0024	20.00	216.45	157.400	0.3886	0.0025	20.00	216.4	157.400	0.3846	0.0024	20.00	222.9	157.400	0.3943	0.0025	20.00	216.4	157.400	0.3930	0.0025	20.00
220.5	164.000	0.4179	0.0025	20.00	220.45	164.000	0.4221	0.0026	20.00	220.4	164.000	0.4012	0.0024	20.00	226.9	164.000	0.4241	0.0026	20.00	220.4	164.000	0.4252	0.0026	20.00
224.5	170.700	0.4543	0.0027	20.00	224.45	170.700	0.4562	0.0027	20.00	224.4	170.700	0.4394	0.0026	20.00	230.9	170.700	0.4315	0.0025	20.00	224.4	170.700	0.4565	0.0027	20.00
228.5	177.400	0.4895	0.0028	20.00	228.45	177.400	0.4819	0.0027	20.00	228.4	177.400	0.4799	0.0027	20.00	234.9	177.400	0.4563	0.0026	20.00	228.4	177.400	0.4756	0.0027	20.00
232.5	184.000	0.5255	0.0029	20.00	232.45	184.000	0.5004	0.0027	20.00	232.4	184.000	0.5153	0.0028	20.00	238.9	184.000	0.4793	0.0026	20.00	232.4	184.000	0.4881	0.0027	20.00
236.5	190.700	0.5598	0.0029	20.00	236.45	190.700	0.5185	0.0027	20.00	236.4	190.700	0.5554	0.0029	20.00	242.9	190.700	0.5002	0.0026	20.00	236.4	190.700	0.5128	0.0027	20.00
240.5	197.400	0.5663	0.0029	20.00	240.45	197.400	0.5414	0.0027	20.00	240.4	197.400	0.5903	0.0030	20.00	246.9	197.400	0.5263	0.0027	20.00	240.4	197.400	0.5378	0.0027	20.00
241.5	199.900	11.8620	0.0593	20.00	241.5	199.950	9.8555	0.0493	20.00	241.4	199.950	9.2765	0.0464	20.00	247.9	199.950	18.6150	0.0931	20.00	241.4	199.917	16.0337	0.0802	20.00
245.55	195.700	0.4110	0.0021	20.00	245.55	195.700	0.3938	0.0020	20.00	245.5	195.700	0.4392	0.0022	20.00	251.9	195.700	0.3815	0.0019	20.00	245.5	195.700	0.3901	0.0020	20.00
249.55	189.050	0.3880	0.0021	20.00	249.55	189.100	0.3645	0.0019	20.00	249.5	189.050	0.3987	0.0021	20.00	255.9	189.100	0.3526	0.0019	20.00	249.5	189.067	0.3633	0.0019	20.00
253.55	182.400	0.3585	0.0020	20.00	253.55	182.400	0.3376	0.0019	20.00	253.5	182.400	0.3705	0.0020	20.00	259.9	182.400	0.3269	0.0018	20.00	253.5	182.400	0.3389	0.0019	20.00
257.55	175.700	0.3383	0.0019	20.00	257.55	175.700	0.3143	0.0018	20.00	257.5	175.700	0.3300	0.0019	20.00	263.9	175.700	0.3056	0.0017	20.00	257.5	175.700	0.3129	0.0018	20.00
261.55	169.100	0.3046	0.0018	20.00	261.55	169.150	0.2952	0.0017	20.00	261.5	169.100	0.2948	0.0017	20.00	267.9	169.400	0.2825	0.0017	20.00	261.5	169.150	0.2964	0.0018	20.00
265.55	162.700	0.2741	0.0017	20.00	265.55	162.700	0.2664	0.0016	20.00	265.5	162.700	0.2646	0.0016	20.00	271.9	162.700	0.2618	0.0016	20.00	265.5	162.700	0.2687	0.0017	20.00
269.55	156.100	0.2406	0.0015	20.00	269.55	156.100	0.2398	0.0015	20.00	269.5	156.100	0.2396	0.0015	20.00	275.9	156.100	0.2341	0.0015	20.00	269.5	156.100	0.2439	0.0016	20.00
273.55	149.400	0.2179	0.0015	20.00	273.55	149.400	0.2092	0.0014	20.00	273.5	149.400	0.2201	0.0015	20.00	279.9	149.400	0.2111	0.0014	20.00	273.5	149.400	0.2144	0.0014	20.00
277.55	142.700	0.1986	0.0014	20.00	277.55	142.700	0.1823	0.0013	20.00	277.5	142.700	0.2059	0.0014	20.00	283.9	142.750	0.1814	0.0013	20.00	277.5	142.700	0.1859	0.0013	20.00
281.55	136.100	0.1822	0.0013	20.00	281.55	136.100	0.1615	0.0012	20.00	281.5	136.100	0.1941	0.0014	20.00	287.9	136.100	0.1586	0.0012	20.00	281.5	136.100	0.1609	0.0012	20.00
285.55	129.400	0.1683	0.0013	20.00	285.55	129.400	0.1487	0.0011	20.00	285.5	129.400	0.1783	0.0014	20.00	291.9	129.400	0.1362	0.0011	20.00	285.5	129.400	0.1451	0.0011	20.00

289.55	122.750	0.1517	0.0012	20.00	289.55	122.750	0.1349	0.0011	20.00	289.5	122.700	0.1667	0.0014	20.00	295.9	122.750	0.1240	0.0010	20.00	289.5	122.717	0.1322	0.0011	20.00
293.55	116.100	0.1392	0.0012	20.00	293.55	116.100	0.1239	0.0011	20.00	293.5	116.100	0.1507	0.0013	20.00	299.9	116.100	0.1084	0.0009	20.00	293.5	116.100	0.1202	0.0010	20.00
297.55	109.400	0.1245	0.0011	20.00	297.55	109.400	0.1097	0.0010	20.00	297.5	109.400	0.1390	0.0013	20.00	303.9	109.400	0.0989	0.0009	20.00	297.5	109.400	0.1076	0.0010	20.00
301.55	102.750	0.1151	0.0011	20.00	301.55	102.750	0.0981	0.0010	20.00	301.5	102.750	0.1221	0.0012	20.00	307.9	102.800	0.0874	0.0009	20.00	301.5	102.767	0.0950	0.0009	20.00
305.55	96.090	0.1003	0.0010	20.00	305.55	96.090	0.0857	0.0009	20.00	305.5	96.090	0.1102	0.0011	20.00	311.9	96.100	0.0765	0.0008	20.00	305.5	96.087	0.0831	0.0009	20.00
309.55	89.420	0.0898	0.0010	20.00	309.55	89.425	0.0740	0.0008	20.00	309.5	89.425	0.0950	0.0011	20.00	315.9	89.430	0.0663	0.0007	20.00	309.5	89.423	0.0710	0.0008	20.00
313.55	82.760	0.0741	0.0009	20.00	313.55	82.760	0.0605	0.0007	20.00	313.5	82.760	0.0849	0.0010	20.00	319.9	82.770	0.0559	0.0007	20.00	313.5	82.758	0.0594	0.0007	20.00
317.55	76.095	0.0643	0.0008	20.00	317.55	76.100	0.0502	0.0007	20.00	317.5	76.100	0.0694	0.0009	20.00	323.9	76.105	0.0447	0.0006	20.00	317.5	76.093	0.0480	0.0006	20.00
321.55	69.430	0.0496	0.0007	20.00	321.55	69.435	0.0393	0.0006	20.00	321.5	69.435	0.0580	0.0008	20.00	327.9	69.935	0.0379	0.0005	20.00	321.5	69.437	0.0403	0.0006	20.00
325.55	62.770	0.0388	0.0006	20.00	325.55	63.280	0.0294	0.0005	20.00	325.5	62.775	0.0444	0.0007	20.00	331.9	63.275	0.0230	0.0004	20.00	325.5	63.267	0.0269	0.0004	20.00
329.55	56.610	0.0265	0.0005	20.00	329.55	56.600	0.0189	0.0003	20.00	329.5	56.215	0.0432	0.0008	20.00	335.9	56.610	0.0129	0.0002	20.00	329.5	56.598	0.0170	0.0003	20.00
333.55	49.935	0.0166	0.0003	20.00	333.55	50.085	0.0118	0.0002	20.00	333.5	49.940	0.0199	0.0004	20.00	339.9	50.280	0.0073	0.0001	20.00	333.5	50.117	0.0105	0.0002	20.00
337.55	43.525	0.0108	0.0002	20.00	337.55	43.715	0.0018	0.0000	20.00	337.5	43.315	0.0104	0.0002	20.00	343.9	43.710	0.0075	0.0002	20.00	337.5	43.705	0.0034	0.0001	20.00
341.55	37.040	0.0062	0.0002	20.00	341.55	37.045	0.0128	0.0003	20.00	341.5	37.050	0.0023	0.0001	20.00	347.9	37.055	0.0159	0.0004	20.00	341.5	37.038	0.0139	0.0004	20.00
345.55	30.370	0.0180	0.0006	20.00	345.55	30.375	0.0246	0.0008	20.00	345.5	30.385	0.0138	0.0005	20.00	351.9	30.005	0.0343	0.0011	20.00	345.5	30.288	0.0305	0.0010	20.00
349.55	23.350	0.0366	0.0016	20.00	349.55	23.215	0.0340	0.0015	20.00	349.5	23.710	0.0267	0.0011	20.00	355.9	23.230	0.0364	0.0016	20.00	349.5	23.213	0.0348	0.0015	20.00
353.55	16.555	0.0415	0.0025	20.00	353.55	16.555	0.0439	0.0027	20.00	353.5	16.550	0.0381	0.0023	20.00	359.9	16.565	0.0462	0.0028	20.00	353.5	16.550	0.0445	0.0027	20.00
357.55	9.888	0.0521	0.0053	20.00	357.55	9.892	0.0540	0.0055	20.00	357.5	9.891	0.0502	0.0051	20.00	363.9	9.899	0.0550	0.0056	20.00	357.5	9.886	0.0542	0.0055	20.00
361.55	3.224	0.0629	0.0195	20.00	361.55	3.227	0.0637	0.0197	20.00	361.5	3.227	0.0624	0.0193	20.00	367.9	3.235	0.0641	0.0198	20.00	361.5	3.222	0.0638	0.0198	20.00

Note: t – time (s); γ – shear rate (1/s); τ – shear stress (Pa); η – dynamic shear viscosity (Pa.s); T – temperature (°C).

Table S2: Rheological data of **C** dispersions (for carrier systems No. 1–15).

1					2					3					4					5				
t (s)	γ (1/s)	τ (Pa)	η (Pa.s)	T (°C)	t (s)	γ (1/s)	τ (Pa)	η (Pa.s)	T (°C)	t (s)	γ (1/s)	τ (Pa)	η (Pa.s)	T (°C)	t (s)	γ (1/s)	τ (Pa)	η (Pa.s)	T (°C)	t (s)	γ (1/s)	τ (Pa)	η (Pa.s)	T (°C)
124.45	4.103	0.0731	0.0178	20.00	124.4	4.102	0.0782	0.0191	20.00	124.35	4.103	0.0734	0.0179	20.01	124.45	4.102	0.0744	0.0181	19.99	124.4	4.103	0.0731	0.0178	20.01
128.45	10.770	0.0798	0.0074	20.00	128.4	10.770	0.0905	0.0084	20.00	128.35	10.770	0.0809	0.0075	20.01	128.45	10.770	0.0832	0.0077	19.99	128.4	10.770	0.0796	0.0074	20.01
132.45	17.430	0.0874	0.0050	20.00	132.4	17.430	0.1036	0.0059	20.00	132.35	17.430	0.0887	0.0051	20.01	132.45	17.430	0.0926	0.0053	19.99	132.4	17.430	0.0865	0.0050	20.00
136.45	24.100	0.0944	0.0039	20.00	136.4	24.095	0.1171	0.0049	20.00	136.35	24.100	0.0966	0.0040	20.01	136.45	24.095	0.1022	0.0042	19.99	136.4	24.100	0.0951	0.0039	20.01
140.45	30.760	0.1031	0.0034	20.00	140.4	30.760	0.1308	0.0043	20.00	140.35	30.760	0.1047	0.0034	20.01	140.45	30.760	0.1126	0.0037	19.99	140.4	30.760	0.1025	0.0033	20.00
144.45	37.425	0.1109	0.0030	20.00	144.4	37.425	0.1432	0.0038	20.00	144.35	37.420	0.1128	0.0030	20.01	144.45	37.425	0.1226	0.0033	19.99	144.4	37.425	0.1114	0.0030	20.00
148.45	44.090	0.1211	0.0027	20.00	148.4	44.090	0.1570	0.0036	20.00	148.35	44.090	0.1219	0.0028	20.01	148.45	44.090	0.1329	0.0030	19.99	148.4	44.090	0.1172	0.0027	20.00
152.45	50.750	0.1303	0.0026	20.00	152.4	50.750	0.1711	0.0034	20.00	152.35	50.750	0.1297	0.0026	20.01	152.45	50.750	0.1445	0.0028	19.99	152.4	50.750	0.1265	0.0025	20.00
156.45	57.420	0.1375	0.0024	20.00	156.4	57.415	0.1850	0.0032	20.00	156.35	57.415	0.1409	0.0025	20.01	156.45	57.420	0.1534	0.0027	19.99	156.4	57.415	0.1346	0.0023	20.00
160.45	64.080	0.1464	0.0023	20.00	160.4	64.085	0.2001	0.0031	20.00	160.35	64.085	0.1482	0.0023	20.01	160.45	64.080	0.1650	0.0026	19.99	160.4	64.085	0.1451	0.0023	20.00
164.45	70.740	0.1535	0.0022	20.00	164.4	70.745	0.2143	0.0030	20.00	164.35	70.745	0.1587	0.0022	20.01	164.45	70.740	0.1749	0.0025	19.99	164.4	70.750	0.1545	0.0022	20.00
168.45	77.410	0.1670	0.0022	20.00	168.4	77.410	0.2288	0.0030	20.00	168.35	77.410	0.1654	0.0021	20.01	168.45	77.410	0.1889	0.0024	20.00	168.4	77.410	0.1622	0.0021	20.00
172.45	84.070	0.1747	0.0021	20.00	172.4	84.070	0.2439	0.0029	20.00	172.35	84.070	0.1744	0.0021	20.01	172.45	84.070	0.2000	0.0024	20.00	172.4	84.070	0.1740	0.0021	20.00
176.45	90.740	0.1859	0.0020	20.00	176.4	90.740	0.2581	0.0028	20.00	176.35	90.740	0.1872	0.0021	20.01	176.45	90.740	0.2109	0.0023	20.00	176.4	90.740	0.1791	0.0020	20.00
180.45	97.395	0.1932	0.0020	20.00	180.4	97.405	0.2743	0.0028	20.00	180.35	97.410	0.1971	0.0020	20.01	180.45	97.395	0.2218	0.0023	20.00	180.4	97.405	0.1919	0.0020	20.00
184.45	104.100	0.2023	0.0019	20.00	184.4	104.100	0.2893	0.0028	20.00	184.35	104.100	0.2096	0.0020	20.01	184.45	104.100	0.2328	0.0022	20.00	184.4	104.100	0.2007	0.0019	20.00
188.45	110.700	0.2155	0.0019	20.00	188.4	110.700	0.3049	0.0028	20.00	188.35	110.700	0.2153	0.0019	20.01	188.45	110.700	0.2478	0.0022	20.00	188.4	110.700	0.2138	0.0019	20.00
192.45	117.400	0.2276	0.0019	20.00	192.4	117.400	0.3212	0.0027	20.00	192.35	117.400	0.2296	0.0020	20.00	192.45	117.400	0.2593	0.0022	20.00	192.4	117.400	0.2357	0.0020	20.00
196.45	124.100	0.2630	0.0021	20.00	196.4	124.100	0.3363	0.0027	20.00	196.35	124.100	0.2549	0.0021	20.00	196.45	124.100	0.2726	0.0022	20.00	196.4	124.100	0.2604	0.0021	20.00
200.45	130.700	0.2868	0.0022	20.00	200.4	130.700	0.3536	0.0027	20.00	200.35	130.700	0.2816	0.0022	20.00	200.45	130.700	0.2845	0.0022	20.00	200.4	130.700	0.2881	0.0022	20.00
204.45	137.400	0.3096	0.0023	20.00	204.4	137.400	0.3694	0.0027	20.00	204.35	137.400	0.3110	0.0023	20.00	204.45	137.400	0.2981	0.0022	20.00	204.4	137.400	0.3110	0.0023	20.00
208.45	144.100	0.3255	0.0023	20.00	208.4	144.050	0.3861	0.0027	20.00	208.35	144.000	0.3276	0.0023	20.00	208.45	144.000	0.3245	0.0023	20.00	208.4	144.050	0.3251	0.0023	20.00
212.45	150.700	0.3409	0.0023	20.00	212.4	150.700	0.4034	0.0027	20.00	212.35	150.700	0.3458	0.0023	20.00	212.45	150.700	0.3594	0.0024	20.00	212.4	150.700	0.3448	0.0023	20.00
216.45	157.400	0.3670	0.0023	20.00	216.4	157.400	0.4184	0.0027	20.00	216.35	157.400	0.3667	0.0023	20.00	216.45	157.400	0.3939	0.0025	20.00	216.4	157.400	0.3583	0.0023	20.00
220.45	164.000	0.3857	0.0024	20.00	220.4	164.000	0.4361	0.0027	20.00	220.35	164.000	0.3911	0.0024	20.00	220.45	164.000	0.4261	0.0026	20.00	220.4	164.000	0.3818	0.0023	20.00
224.45	170.700	0.4076	0.0024	20.00	224.4	170.700	0.4508	0.0026	20.00	224.35	170.700	0.4163	0.0024	20.00	224.45	170.700	0.4494	0.0026	20.00	224.4	170.700	0.4040	0.0024	20.00
228.45	177.400	0.4364	0.0025	20.00	228.4	177.400	0.4694	0.0026	20.00	228.35	177.400	0.4345	0.0024	20.00	228.45	177.400	0.4690	0.0026	20.00	228.4	177.400	0.4262	0.0024	20.00
232.45	184.000	0.4545	0.0025	20.00	232.4	184.000	0.4931	0.0027	20.00	232.35	184.000	0.4559	0.0025	20.00	232.45	184.000	0.4909	0.0027	20.00	232.4	184.000	0.4520	0.0025	20.00
236.45	190.700	0.4768	0.0025	20.00	236.4	190.700	0.5436	0.0029	20.00	236.35	190.700	0.4821	0.0025	20.00	236.45	190.700	0.5143	0.0027	20.00	236.4	190.700	0.4708	0.0025	20.00
240.45	197.400	0.4939	0.0025	20.00	240.4	197.400	0.5881	0.0030	20.00	240.35	197.400	0.5009	0.0025	20.00	240.45	197.400	0.5478	0.0028	20.00	240.4	197.400	0.4996	0.0025	20.00

241.45	199.950	13.9800	0.0699	20.00	241.4	199.900	6.5106	0.0326	20.00	241.35	199.900	18.2500	0.0913	20.00	241.5	199.900	12.1000	0.0605	20.00	241.45	199.900	5.5115	0.0276	20.00
245.5	195.700	0.3517	0.0018	20.00	245.5	195.700	0.4371	0.0022	20.00	245.4	195.700	0.3534	0.0018	20.00	245.5	195.700	0.3920	0.0020	20.00	245.45	195.700	0.3515	0.0018	20.00
249.5	189.000	0.3218	0.0017	20.00	249.5	189.000	0.3991	0.0021	20.00	249.4	189.000	0.3302	0.0017	20.00	249.5	189.100	0.3636	0.0019	20.00	249.45	189.050	0.3340	0.0018	20.00
253.5	182.400	0.3068	0.0017	20.00	253.5	182.400	0.3523	0.0019	20.00	253.4	182.400	0.3025	0.0017	20.00	253.5	182.400	0.3415	0.0019	20.00	253.45	182.400	0.3040	0.0017	20.00
257.5	176.100	0.2752	0.0016	20.00	257.5	175.700	0.3226	0.0018	20.00	257.4	176.050	0.2904	0.0017	20.00	257.5	175.700	0.3130	0.0018	20.00	257.45	176.100	0.2922	0.0017	20.00
261.5	169.400	0.2566	0.0015	20.00	261.5	169.100	0.2994	0.0018	20.00	261.4	169.400	0.2668	0.0016	20.00	261.5	169.200	0.2964	0.0018	20.00	261.45	169.400	0.2590	0.0015	20.00
265.5	162.700	0.2332	0.0014	20.00	265.5	162.700	0.2890	0.0018	20.00	265.4	162.700	0.2422	0.0015	20.00	265.5	162.700	0.2671	0.0016	20.00	265.45	162.700	0.2409	0.0015	20.00
269.5	156.100	0.2137	0.0014	20.00	269.5	156.100	0.2677	0.0017	20.00	269.4	156.100	0.2251	0.0014	20.00	269.5	156.100	0.2424	0.0016	20.00	269.45	156.100	0.2179	0.0014	20.00
273.5	149.400	0.1899	0.0013	20.00	273.5	149.400	0.2541	0.0017	20.00	273.4	149.400	0.2008	0.0013	20.00	273.5	149.400	0.2132	0.0014	20.00	273.45	149.400	0.1997	0.0013	20.00
277.5	142.700	0.1701	0.0012	20.00	277.5	142.700	0.2363	0.0017	20.00	277.4	142.700	0.1818	0.0013	20.00	277.5	142.700	0.1826	0.0013	20.00	277.45	142.700	0.1764	0.0012	20.00
281.5	136.100	0.1571	0.0012	20.01	281.5	136.100	0.2183	0.0016	20.00	281.4	136.100	0.1572	0.0012	20.00	281.5	136.100	0.1587	0.0012	20.00	281.45	136.100	0.1562	0.0011	20.00
285.5	129.400	0.1359	0.0010	20.00	285.5	129.400	0.2032	0.0016	20.00	285.4	129.400	0.1392	0.0011	20.00	285.5	129.400	0.1407	0.0011	20.00	285.45	129.400	0.1373	0.0011	20.00
289.5	122.700	0.1181	0.0010	20.00	289.5	122.700	0.1852	0.0015	20.00	289.4	122.700	0.1139	0.0009	20.00	289.5	122.800	0.1297	0.0011	20.00	289.45	122.700	0.1105	0.0009	20.00
293.5	116.100	0.0910	0.0008	20.00	293.5	116.100	0.1709	0.0015	20.00	293.4	116.100	0.0961	0.0008	20.00	293.5	116.100	0.1152	0.0010	20.00	293.45	116.100	0.0892	0.0008	20.00
297.5	109.400	0.0754	0.0007	20.00	297.5	109.400	0.1541	0.0014	20.00	297.4	109.400	0.0754	0.0007	20.00	297.5	109.400	0.1043	0.0010	20.00	297.45	109.400	0.0667	0.0006	20.00
301.5	102.700	0.0599	0.0006	20.00	301.5	102.800	0.1425	0.0014	20.00	301.4	102.700	0.0648	0.0006	20.00	301.5	102.800	0.0906	0.0009	20.00	301.45	102.800	0.0617	0.0006	20.00
305.5	96.085	0.0525	0.0005	20.00	305.5	96.090	0.1260	0.0013	20.00	305.4	96.080	0.0538	0.0006	20.00	305.5	96.095	0.0800	0.0008	20.00	305.45	96.090	0.0492	0.0005	20.00
309.5	89.420	0.0411	0.0005	20.00	309.5	89.425	0.1136	0.0013	20.00	309.4	89.420	0.0431	0.0005	20.00	309.5	89.435	0.0678	0.0008	20.00	309.45	89.430	0.0477	0.0005	20.00
313.5	83.260	0.0347	0.0004	20.00	313.5	82.760	0.0961	0.0012	20.00	313.4	83.245	0.0375	0.0005	20.00	313.5	82.770	0.0580	0.0007	20.00	313.45	83.255	0.0312	0.0004	20.00
317.5	76.590	0.0235	0.0003	20.00	317.5	76.095	0.0839	0.0011	20.00	317.4	76.585	0.0246	0.0003	20.00	317.5	76.105	0.0450	0.0006	20.00	317.45	76.595	0.0253	0.0003	20.00
321.5	69.925	0.0177	0.0003	20.00	321.5	69.430	0.0678	0.0010	20.00	321.4	69.925	0.0171	0.0002	20.00	321.5	69.670	0.0454	0.0007	20.00	321.45	69.940	0.0137	0.0002	20.00
325.5	63.460	0.0057	0.0001	20.00	325.5	62.765	0.0547	0.0009	20.00	325.4	63.430	0.0093	0.0001	20.00	325.5	63.275	0.0243	0.0004	20.00	325.45	63.480	0.0122	0.0002	20.00
329.5	57.035	0.0028	0.0000	20.00	329.5	56.100	0.0399	0.0007	20.00	329.4	57.035	0.0014	0.0000	20.00	329.5	56.610	0.0143	0.0003	20.00	329.45	57.025	0.0030	0.0001	20.00
333.5	50.365	0.0120	0.0002	20.00	333.5	49.845	0.0381	0.0008	20.00	333.4	50.365	0.0087	0.0002	20.00	333.5	50.245	0.0081	0.0002	20.00	333.45	50.365	0.0083	0.0002	20.00
337.5	43.700	0.0209	0.0005	20.00	337.5	43.270	0.0136	0.0003	20.00	337.4	43.700	0.0162	0.0004	20.00	337.5	43.710	0.0061	0.0001	20.00	337.45	43.700	0.0206	0.0005	20.00
341.5	36.540	0.0267	0.0007	20.00	341.5	37.035	0.0055	0.0001	20.00	341.4	36.525	0.0270	0.0007	20.00	341.5	37.050	0.0157	0.0004	20.00	341.45	36.530	0.0285	0.0008	20.00
345.5	29.875	0.0361	0.0012	20.00	345.5	30.375	0.0113	0.0004	20.00	345.4	29.875	0.0348	0.0012	20.00	345.5	30.075	0.0359	0.0012	20.00	345.45	29.875	0.0358	0.0012	20.00
349.5	23.210	0.0430	0.0019	20.00	349.5	23.685	0.0293	0.0012	20.00	349.4	23.210	0.0433	0.0019	20.00	349.5	23.225	0.0363	0.0016	20.00	349.45	23.215	0.0448	0.0019	20.00
353.5	16.550	0.0514	0.0031	20.00	353.5	16.550	0.0382	0.0023	20.00	353.4	16.550	0.0500	0.0030	20.00	353.5	16.560	0.0460	0.0028	20.00	353.45	16.545	0.0514	0.0031	20.00
357.5	9.884	0.0582	0.0059	20.00	357.5	9.887	0.0505	0.0051	20.00	357.4	9.883	0.0581	0.0059	20.00	357.5	9.896	0.0552	0.0056	20.00	357.45	9.886	0.0588	0.0059	20.00
361.5	3.219	0.0654	0.0203	20.00	361.5	3.224	0.0628	0.0195	20.00	361.4	3.219	0.0653	0.0203	20.00	361.5	3.232	0.0643	0.0199	20.00	361.45	3.222	0.0655	0.0203	20.00

6					7					8					9					10				
t (s)	γ (1/s)	τ (Pa)	η (Pa.s)	T (°C)	t (s)	γ (1/s)	τ (Pa)	η (Pa.s)	T (°C)	t (s)	γ (1/s)	τ (Pa)	η (Pa.s)	T (°C)	t (s)	γ (1/s)	τ (Pa)	η (Pa.s)	T (°C)	t (s)	γ (1/s)	τ (Pa)	η (Pa.s)	T (°C)
124.45	4.103	0.0760	0.0185	20.00	124.4	4.103	0.0732	0.0178	20.00	124.4	4.103	0.0753	0.0184	20.00	124.4	4.102	0.0738	0.0180	20.00	124.35	4.103	0.0776	0.0189	20.00
128.45	10.770	0.0878	0.0081	20.00	128.4	10.770	0.0798	0.0074	20.00	128.4	10.770	0.0860	0.0080	20.00	128.4	10.770	0.0798	0.0074	20.00	128.35	10.770	0.0910	0.0085	20.00
132.45	17.430	0.1002	0.0057	20.00	132.4	17.430	0.0871	0.0050	20.00	132.4	17.430	0.0970	0.0056	20.00	132.4	17.430	0.0874	0.0050	20.00	132.35	17.430	0.1048	0.0060	20.00
136.45	24.100	0.1122	0.0047	20.00	136.4	24.100	0.0952	0.0040	20.00	136.4	24.100	0.1084	0.0045	20.00	136.4	24.095	0.0947	0.0039	20.00	136.35	24.095	0.1188	0.0049	20.00
140.45	30.760	0.1252	0.0041	20.00	140.4	30.760	0.1033	0.0034	20.00	140.4	30.760	0.1197	0.0039	20.00	140.4	30.760	0.1033	0.0034	20.00	140.35	30.760	0.1331	0.0043	20.00
144.45	37.425	0.1379	0.0037	20.00	144.4	37.430	0.1122	0.0030	20.00	144.4	37.425	0.1312	0.0035	20.00	144.4	37.425	0.1117	0.0030	20.00	144.35	37.425	0.1475	0.0039	20.00
148.45	44.090	0.1524	0.0035	20.00	148.4	44.090	0.1195	0.0027	20.00	148.4	44.090	0.1433	0.0033	20.00	148.4	44.090	0.1208	0.0027	20.00	148.35	44.090	0.1618	0.0037	20.00
152.45	50.750	0.1660	0.0033	20.00	152.4	50.750	0.1296	0.0026	20.00	152.4	50.755	0.1550	0.0031	20.00	152.4	50.750	0.1314	0.0026	20.00	152.35	50.750	0.1764	0.0035	20.00
156.45	57.415	0.1793	0.0031	20.00	156.4	57.420	0.1363	0.0024	20.00	156.4	57.420	0.1679	0.0029	20.00	156.4	57.420	0.1370	0.0024	20.00	156.35	57.420	0.1921	0.0033	20.00
160.45	64.080	0.1921	0.0030	20.00	160.4	64.085	0.1470	0.0023	20.00	160.4	64.085	0.1796	0.0028	20.00	160.4	64.075	0.1466	0.0023	20.00	160.35	64.080	0.2065	0.0032	20.00
164.45	70.745	0.2054	0.0029	20.00	164.4	70.750	0.1553	0.0022	20.00	164.4	70.745	0.1917	0.0027	20.00	164.4	70.745	0.1540	0.0022	20.00	164.35	70.745	0.2224	0.0031	20.00
168.45	77.410	0.2207	0.0029	20.00	168.4	77.410	0.1672	0.0022	20.00	168.4	77.410	0.2041	0.0026	20.00	168.4	77.410	0.1685	0.0022	20.00	168.35	77.410	0.2367	0.0031	20.00
172.45	84.070	0.2335	0.0028	20.00	172.4	84.070	0.1784	0.0021	20.00	172.4	84.075	0.2168	0.0026	20.00	172.4	84.070	0.1777	0.0021	20.00	172.35	84.075	0.2522	0.0030	20.00
176.45	90.735	0.2497	0.0028	20.00	176.4	90.740	0.1842	0.0020	20.00	176.4	90.740	0.2310	0.0025	20.00	176.4	90.735	0.1859	0.0020	20.00	176.35	90.740	0.2678	0.0030	20.00
180.45	97.405	0.2626	0.0027	20.00	180.4	97.400	0.1955	0.0020	20.00	180.4	97.400	0.2438	0.0025	20.00	180.4	97.400	0.1947	0.0020	20.00	180.35	97.400	0.2841	0.0029	20.00
184.45	104.100	0.2774	0.0027	20.00	184.4	104.100	0.2036	0.0020	20.00	184.4	104.100	0.2578	0.0025	20.00	184.4	104.100	0.2025	0.0019	20.00	184.35	104.100	0.3002	0.0029	20.00
188.45	110.700	0.2915	0.0026	20.00	188.4	110.700	0.2193	0.0020	20.00	188.4	110.700	0.2705	0.0024	20.00	188.4	110.700	0.2198	0.0020	20.00	188.35	110.700	0.3157	0.0029	20.00
192.45	117.400	0.3052	0.0026	20.00	192.4	117.400	0.2357	0.0020	20.00	192.4	117.400	0.2844	0.0024	20.00	192.4	117.400	0.2409	0.0021	20.00	192.35	117.400	0.3324	0.0028	20.00
196.45	124.100	0.3231	0.0026	20.00	196.4	124.100	0.2641	0.0021	20.00	196.4	124.100	0.2998	0.0024	20.00	196.4	124.100	0.2715	0.0022	20.00	196.35	124.100	0.3480	0.0028	20.00
200.45	130.700	0.3373	0.0026	20.00	200.4	130.700	0.2913	0.0022	20.00	200.4	130.700	0.3139	0.0024	20.00	200.4	130.700	0.2946	0.0023	20.00	200.35	130.700	0.3649	0.0028	20.00
204.45	137.400	0.3533	0.0026	20.00	204.4	137.400	0.3158	0.0023	20.00	204.4	137.400	0.3292	0.0024	20.00	204.4	137.400	0.3174	0.0023	20.00	204.35	137.400	0.3819	0.0028	20.00
208.45	144.050	0.3684	0.0026	20.00	208.4	144.000	0.3378	0.0023	20.00	208.4	144.100	0.3430	0.0024	20.00	208.4	144.050	0.3416	0.0024	20.00	208.35	144.050	0.3984	0.0028	20.00
212.45	150.700	0.3832	0.0025	20.00	212.4	150.700	0.3519	0.0023	20.00	212.4	150.700	0.3586	0.0024	20.00	212.4	150.700	0.3547	0.0024	20.00	212.35	150.700	0.4159	0.0028	20.00
216.45	157.400	0.4028	0.0026	20.00	216.4	157.400	0.3683	0.0023	20.00	216.4	157.400	0.3773	0.0024	20.00	216.4	157.400	0.3750	0.0024	20.00	216.35	157.400	0.4325	0.0027	20.00
220.45	164.000	0.4187	0.0026	20.00	220.4	164.000	0.3896	0.0024	20.00	220.4	164.000	0.4139	0.0025	20.00	220.4	164.000	0.3906	0.0024	20.00	220.35	164.000	0.4504	0.0027	20.00
224.45	170.700	0.4363	0.0026	20.00	224.4	170.700	0.4103	0.0024	20.00	224.4	170.700	0.4535	0.0027	20.00	224.4	170.700	0.4085	0.0024	20.00	224.35	170.700	0.4691	0.0027	20.00

228.45	177.400	0.4617	0.0026	20.00	228.4	177.400	0.4384	0.0025	20.00	228.4	177.400	0.4879	0.0028	20.00	228.4	177.400	0.4340	0.0024	20.00	228.35	177.400	0.4857	0.0027	20.00
232.45	184.000	0.5090	0.0028	20.00	232.4	184.000	0.4578	0.0025	20.00	232.4	184.000	0.5260	0.0029	20.00	232.4	184.000	0.4541	0.0025	20.00	232.35	184.000	0.5046	0.0027	20.00
236.45	190.700	0.5539	0.0029	20.00	236.4	190.700	0.4757	0.0025	20.00	236.4	190.700	0.5649	0.0030	20.00	236.4	190.700	0.4738	0.0025	20.00	236.35	190.700	0.5259	0.0028	20.00
240.45	197.400	0.5940	0.0030	20.00	240.4	197.400	0.4978	0.0025	20.00	240.4	197.400	0.5774	0.0029	20.00	240.4	197.400	0.4981	0.0025	20.00	240.35	197.400	0.5671	0.0029	20.00
241.45	199.950	16.8700	0.0844	20.00	241.4	199.900	12.1260	0.0607	20.00	241.4	199.950	10.6655	0.0533	20.00	241.4	200.000	13.8695	0.0694	20.00	241.35	199.950	10.7895	0.0540	20.00
245.5	195.700	0.4444	0.0023	20.00	245.5	195.700	0.3475	0.0018	20.00	245.45	195.700	0.4238	0.0022	20.00	245.5	195.700	0.3498	0.0018	20.00	245.45	195.700	0.4242	0.0022	20.00
249.5	189.050	0.3998	0.0021	20.00	249.5	189.050	0.3324	0.0018	20.00	249.45	189.050	0.3940	0.0021	20.00	249.5	189.100	0.3232	0.0017	20.00	249.45	189.050	0.3836	0.0020	20.00
253.5	182.400	0.3647	0.0020	20.00	253.5	182.400	0.3063	0.0017	20.00	253.45	182.400	0.3666	0.0020	20.00	253.5	182.400	0.3080	0.0017	20.00	253.45	182.400	0.3560	0.0020	20.00
257.5	175.700	0.3222	0.0018	20.00	257.5	176.100	0.2858	0.0016	20.00	257.45	175.700	0.3348	0.0019	20.00	257.5	176.100	0.2822	0.0016	20.00	257.45	175.700	0.3383	0.0019	20.00
261.5	169.100	0.2951	0.0017	20.00	261.5	169.400	0.2575	0.0015	20.00	261.45	169.100	0.3053	0.0018	20.00	261.5	169.400	0.2587	0.0015	20.00	261.45	169.050	0.3228	0.0019	20.00
265.5	162.700	0.2736	0.0017	20.00	265.5	162.700	0.2396	0.0015	20.00	265.45	162.700	0.2690	0.0017	20.00	265.5	162.700	0.2372	0.0015	20.00	265.45	162.400	0.3047	0.0019	20.00
269.5	156.100	0.2564	0.0016	20.00	269.5	156.100	0.2140	0.0014	20.00	269.45	156.100	0.2389	0.0015	20.00	269.5	156.100	0.2139	0.0014	20.00	269.45	155.900	0.2964	0.0019	20.00
273.5	149.400	0.2378	0.0016	20.00	273.5	149.400	0.2011	0.0013	20.00	273.45	149.400	0.2132	0.0014	20.00	273.5	149.400	0.1964	0.0013	20.00	273.45	149.400	0.2725	0.0018	20.00
277.5	142.700	0.2230	0.0016	20.00	277.5	142.700	0.1775	0.0012	20.00	277.45	142.700	0.2004	0.0014	20.00	277.5	142.700	0.1733	0.0012	20.00	277.45	142.700	0.2562	0.0018	20.00
281.5	136.100	0.2095	0.0015	20.00	281.5	136.100	0.1616	0.0012	20.00	281.45	136.100	0.1824	0.0013	20.00	281.5	136.100	0.1602	0.0012	20.00	281.45	136.100	0.2368	0.0017	20.00
285.5	129.400	0.1930	0.0015	20.00	285.5	129.400	0.1390	0.0011	20.00	285.45	129.400	0.1699	0.0013	20.00	285.5	129.400	0.1374	0.0011	20.00	285.45	129.400	0.2213	0.0017	20.00
289.5	122.750	0.1804	0.0015	20.00	289.5	122.750	0.1148	0.0009	20.00	289.45	122.700	0.1553	0.0013	20.00	289.5	122.750	0.1175	0.0010	20.00	289.45	122.750	0.2033	0.0017	20.00
293.5	116.100	0.1637	0.0014	20.00	293.5	116.100	0.0875	0.0008	20.00	293.45	116.100	0.1445	0.0012	20.00	293.5	116.100	0.0895	0.0008	20.00	293.45	116.100	0.1890	0.0016	20.00
297.5	109.400	0.1506	0.0014	20.00	297.5	109.400	0.0708	0.0006	20.00	297.45	109.400	0.1286	0.0012	20.00	297.5	109.400	0.0734	0.0007	20.00	297.45	109.400	0.1709	0.0016	20.00
301.5	102.800	0.1330	0.0013	20.00	301.5	102.750	0.0637	0.0006	20.00	301.45	102.750	0.1139	0.0011	20.00	301.5	102.750	0.0597	0.0006	20.00	301.45	102.750	0.1570	0.0015	20.00
305.5	96.095	0.1198	0.0012	20.00	305.5	96.085	0.0530	0.0006	20.00	305.45	96.090	0.1008	0.0010	20.00	305.5	96.095	0.0514	0.0005	20.00	305.45	96.085	0.1402	0.0015	20.00
309.5	89.430	0.1040	0.0012	20.00	309.5	89.420	0.0473	0.0005	20.00	309.45	89.430	0.0860	0.0010	20.00	309.5	89.425	0.0427	0.0005	20.00	309.45	89.425	0.1254	0.0014	20.00
313.5	82.765	0.0924	0.0011	20.00	313.5	83.270	0.0382	0.0005	20.00	313.45	82.760	0.0725	0.0009	20.00	313.5	83.260	0.0353	0.0004	20.00	313.45	82.755	0.1082	0.0013	20.00
317.5	76.100	0.0760	0.0010	20.00	317.5	76.595	0.0283	0.0004	20.00	317.45	76.095	0.0604	0.0008	20.00	317.5	76.595	0.0246	0.0003	20.00	317.45	76.095	0.0942	0.0012	20.00
321.5	69.440	0.0640	0.0009	20.00	321.5	69.925	0.0150	0.0002	20.00	321.45	69.435	0.0502	0.0007	20.00	321.5	69.930	0.0171	0.0002	20.00	321.45	69.430	0.0790	0.0011	20.00
325.5	62.770	0.0493	0.0008	20.00	325.5	63.405	0.0100	0.0002	20.00	325.45	62.770	0.0371	0.0006	20.00	325.5	63.435	0.0095	0.0001	20.00	325.45	62.770	0.0645	0.0010	20.00
329.5	56.110	0.0370	0.0007	20.00	329.5	57.045	0.0028	0.0000	20.00	329.45	56.610	0.0276	0.0005	20.00	329.5	57.045	0.0029	0.0001	20.00	329.45	56.105	0.0497	0.0009	20.00
333.5	49.955	0.0234	0.0005	20.00	333.5	50.365	0.0079	0.0002	20.00	333.45	49.940	0.0153	0.0003	20.00	333.5	50.370	0.0109	0.0002	20.00	333.45	49.440	0.0356	0.0007	20.00
337.5	43.275	0.0112	0.0003	20.00	337.5	43.705	0.0182	0.0004	20.00	337.45	43.590	0.0105	0.0002	20.00	337.5	43.705	0.0207	0.0005	20.00	337.45	43.280	0.0209	0.0005	20.00
341.5	37.045	0.0019	0.0001	20.00	341.5	36.525	0.0280	0.0008	20.00	341.45	37.040	0.0080	0.0002	20.00	341.5	36.535	0.0275	0.0008	20.00	341.45	36.625	0.0100	0.0003	20.00
345.5	30.380	0.0132	0.0004	20.00	345.5	29.875	0.0353	0.0012	20.00	345.45	30.375	0.0189	0.0006	20.00	345.5	29.880	0.0360	0.0012	20.00	345.45	30.375	0.0063	0.0002	20.00
349.5	23.610	0.0318	0.0013	20.00	349.5	23.220	0.0437	0.0019	20.00	349.45	23.240	0.0361	0.0016	20.00	349.5	23.225	0.0433	0.0019	20.00	349.45	23.715	0.0206	0.0009	20.00
353.5	16.555	0.0387	0.0023	20.00	353.5	16.550	0.0514	0.0031	20.00	353.45	16.550	0.0419	0.0025	20.00	353.5	16.555	0.0516	0.0031	20.00	353.45	16.575	0.0410	0.0025	20.00
357.5	9.892	0.0510	0.0052	20.00	357.5	9.887	0.0584	0.0059	20.00	357.45	9.889	0.0531	0.0054	20.00	357.5	9.890	0.0582	0.0059	20.00	357.45	9.886	0.0487	0.0049	20.00
361.5	3.228	0.0630	0.0195	20.00	361.5	3.223	0.0654	0.0203	20.00	361.45	3.225	0.0637	0.0197	20.00	361.5	3.226	0.0654	0.0203	20.00	361.45	3.223	0.0622	0.0193	20.00
11					12					13					14					15				
t (s)	γ (1/s)	τ (Pa)	η (Pa.s)	T (°C)	t (s)	γ (1/s)	τ (Pa)	η (Pa.s)	T (°C)	t (s)	γ (1/s)	τ (Pa)	η (Pa.s)	T (°C)	t (s)	γ (1/s)	τ (Pa)	η (Pa.s)	T (°C)	t (s)	γ (1/s)	τ (Pa)	η (Pa.s)	T (°C)
124.4	4.103	0.0752	0.0183	20.00	124.5	4.103	0.0742	0.0181	19.99	124.45	4.103	0.0756	0.0184	19.99	124.4	4.103	0.0740	0.0180	19.99	124.525	4.103	0.0749	0.0183	19.99
128.4	10.770	0.0860	0.0080	20.00	128.5	10.770	0.0829	0.0077	19.99	128.45	10.770	0.0860	0.0080	19.99	128.4	10.770	0.0826	0.0077	19.99	128.525	10.770	0.0842	0.0078	19.99
132.4	17.430	0.0972	0.0056	20.00	132.5	17.430	0.0924	0.0053	20.00	132.45	17.430	0.0966	0.0055	19.99	132.4	17.430	0.0920	0.0053	19.99	132.525	17.430	0.0941	0.0054	19.99
136.4	24.100	0.1083	0.0045	20.00	136.5	24.095	0.1014	0.0042	20.00	136.45	24.100	0.1084	0.0045	19.99	136.4	24.100	0.1008	0.0042	19.99	136.525	24.098	0.1040	0.0043	19.99
140.4	30.760	0.1199	0.0039	20.00	140.5	30.760	0.1119	0.0036	20.00	140.45	30.760	0.1191	0.0039	19.99	140.4	30.760	0.1107	0.0036	19.99	140.525	30.760	0.1146	0.0037	19.99
144.4	37.425	0.1314	0.0035	20.00	144.5	37.425	0.1217	0.0033	20.00	144.45	37.425	0.1311	0.0035	20.00	144.4	37.425	0.1201	0.0032	19.99	144.525	37.425	0.1249	0.0033	19.99
148.4	44.090	0.1444	0.0033	20.00	148.5	44.090	0.1330	0.0030	20.00	148.45	44.090	0.1406	0.0032	20.00	148.4	44.090	0.1314	0.0030	20.00	148.525	44.090	0.1361	0.0031	19.99
152.4	50.750	0.1557	0.0031	20.00	152.5	50.750	0.1449	0.0029	20.00	152.45	50.750	0.1515	0.0030	20.00	152.4	50.750	0.1411	0.0028	20.00	152.525	50.750	0.1473	0.0029	19.99
156.4	57.415	0.1697	0.0030	20.00	156.5	57.420	0.1539	0.0027	20.00	156.45	57.415	0.1646	0.0029	20.00	156.4	57.415	0.1529	0.0027	20.00	156.525	57.418	0.1580	0.0028	19.99
160.4	64.080	0.1810	0.0028	20.00	160.5	64.080	0.1653	0.0026	20.00	160.45	64.080	0.1775	0.0028	20.00	160.4	64.080	0.1615	0.0025	20.00	160.525	64.083	0.1693	0.0026	19.99
164.4	70.745	0.1938	0.0027	20.00	164.5	70.745	0.1746	0.0025	20.00	164.45	70.750	0.1910	0.0027	20.00	164.4	70.745	0.1725	0.0024	20.00	164.525	70.745	0.1801	0.0025	19.99
168.4	77.410	0.2051	0.0026	20.00	168.5	77.410	0.1897	0.0025	20.00	168.45	77.410	0.2015	0.0026	20.00	168.4	77.410	0.1833	0.0024	20.00	168.525	77.410	0.1924	0.0025	19.99
172.4	84.075	0.2167	0.0026	20.00	172.5	84.070	0.1998	0.0024	20.00	172.45	84.075	0.2160	0.0026	20.00	172.4	84.070	0.1926	0.0023	20.00	172.525	84.073	0.2036	0.0024	19.99
176.4	90.740	0.2334	0.0026	20.00	176.5	90.735	0.2117	0.0023	20.00	176.45	90.740	0.2260	0.0025	20.00	176.4	90.740	0.2074	0.0023	20.00	176.525	90.738	0.2165	0.0024	20.00
180.4	97.400	0.2459	0.0025	20.00	180.5	97.400	0.2220	0.0023	20.00	180.45	97.400	0.2418	0.0025	20.00	180.4	97.405	0.2164	0.0022	20.00	180.525	97.403	0.2278	0.0023	20.00
184.4	104.100	0.2612	0.0025	20.00	184.5	104.100	0.2327	0.0022	20.00	184.45	104.100	0.2550	0.0025	20.00	184.4	104.100	0.2292	0.0022	20.00	184.525	104.100	0.2393	0.0023	20.00
188.4	110.700	0.2708	0.0024	20.00	188.5	110.700	0.2480	0.0022	20.00	188.45	110.700	0.2679	0.0024	20.00	188.4	110.700	0.2383	0.0022	20.00	188.525	110.700	0.2525	0.0023	20.00
192.4	117.400	0.2846	0.0024	20.00	192.5	117.400	0.2584	0.0022	20.00	192.45	117.400	0.2840	0.0024	20.00	192.4	117.400	0.2493	0.0021	20.00	192.525	117.400	0.2644	0.0023	20.00
196.4	124.100	0.3020	0.0024	20.00	196.5	124.100	0.2738	0.0022	20.00	196.45	124.100	0.2900	0.0023	20.00	196.4	124.100	0.2657	0.0021	20.00	196.525	124.100	0.2790	0.0022	20.00
200.4	130.700	0.3162	0.0024	20.00	200.5	130.700	0.2843	0.0022	20.00	200.45	130.700	0.3070	0.0023	20.00	200.4	130.700	0.2777	0.0021	20.00	200.525	130.700	0.2915	0.0022	19.99
204.4	137.400	0.3330	0.0024	20.00	204.5	137.400	0.2969	0.0022	20.00	204.45	137.400	0.3208	0.0023	20.00	204.4	137.400	0.2949	0.0021	20.00	204.525	137.400	0.3054	0.0022	19.99
208.4	144.050	0.3430	0.0024	20.00	208.5	144.050	0.3255	0.0023	20.00	208.45	144.100	0.3369	0.0023	20.00	208.4	144.050	0.3300	0.0023	20.00	208.525	144.050	0.3237	0.0022	19.99

212.4	150.700	0.3583	0.0024	20.00	212.5	150.700	0.3621	0.0024	20.00	212.45	150.700	0.3549	0.0024	20.00	212.4	150.700	0.3614	0.0024	20.00	212.525	150.700	0.3615	0.0024	20.00
216.4	157.400	0.3780	0.0024	20.00	216.5	157.400	0.3983	0.0025	20.00	216.45	157.400	0.3748	0.0024	20.00	216.4	157.400	0.3977	0.0025	20.00	216.525	157.400	0.3969	0.0025	20.00
220.4	164.000	0.3992	0.0024	20.00	220.5	164.000	0.4294	0.0026	20.00	220.45	164.000	0.4179	0.0025	20.00	220.4	164.000	0.4260	0.0026	20.00	220.525	164.000	0.4296	0.0026	20.00
224.4	170.700	0.4478	0.0026	20.00	224.5	170.700	0.4598	0.0027	20.00	224.45	170.700	0.4546	0.0027	20.00	224.4	170.700	0.4353	0.0025	20.00	224.525	170.700	0.4625	0.0027	20.00
228.4	177.400	0.4830	0.0027	20.00	228.5	177.400	0.4705	0.0027	20.00	228.45	177.400	0.4897	0.0028	20.00	228.4	177.400	0.4533	0.0026	20.00	228.525	177.400	0.4877	0.0027	20.00
232.4	184.000	0.5216	0.0028	20.00	232.5	184.000	0.4923	0.0027	20.00	232.45	184.000	0.5273	0.0029	20.00	232.4	184.000	0.4755	0.0026	20.00	232.525	184.000	0.5008	0.0027	20.00
236.4	190.700	0.5657	0.0030	20.00	236.5	190.700	0.5156	0.0027	20.00	236.45	190.700	0.5436	0.0029	20.00	236.4	190.700	0.5073	0.0027	20.00	236.525	190.700	0.5253	0.0028	20.00
240.4	197.400	0.5830	0.0030	20.00	240.5	197.400	0.5400	0.0027	20.00	240.45	197.400	0.5641	0.0029	20.00	240.4	197.400	0.5288	0.0027	20.00	240.525	197.400	0.5505	0.0028	20.00
241.45	199.900	9.9345	0.0497	20.00	241.5	199.950	18.5350	0.0927	20.00	241.45	199.900	16.0250	0.0802	20.00	241.4	199.950	12.4155	0.0621	20.00	241.55	199.950	10.6036	0.0530	20.00
245.5	195.700	0.4207	0.0021	20.00	245.55	195.700	0.3929	0.0020	20.00	245.55	195.700	0.4131	0.0021	20.00	245.5	195.700	0.3797	0.0019	20.00	245.6	195.700	0.4021	0.0021	20.00
249.5	189.050	0.3913	0.0021	20.00	249.55	189.100	0.3656	0.0019	20.00	249.55	189.000	0.3889	0.0021	20.00	249.5	189.000	0.3487	0.0018	20.00	249.6	189.100	0.3753	0.0020	20.00
253.5	182.400	0.3667	0.0020	20.00	253.55	182.400	0.3467	0.0019	20.00	253.55	182.400	0.3577	0.0020	20.00	253.5	182.400	0.3306	0.0018	20.00	253.6	182.400	0.3509	0.0019	20.00
257.5	175.700	0.3336	0.0019	20.00	257.55	175.700	0.3201	0.0018	20.00	257.55	175.700	0.3355	0.0019	20.00	257.5	175.700	0.3014	0.0017	20.00	257.6	175.700	0.3260	0.0019	20.00
261.5	169.100	0.3006	0.0018	20.00	261.55	169.100	0.2956	0.0017	20.00	261.55	169.050	0.3046	0.0018	20.00	261.5	169.300	0.2947	0.0017	20.00	261.6	169.100	0.3036	0.0018	20.00
265.5	162.700	0.2639	0.0016	20.00	265.55	162.700	0.2733	0.0017	20.00	265.55	162.700	0.2726	0.0017	20.00	265.5	162.700	0.2627	0.0016	20.00	265.6	162.675	0.2804	0.0017	20.00
269.5	156.100	0.2367	0.0015	20.00	269.55	156.100	0.2463	0.0016	20.00	269.55	156.100	0.2393	0.0015	20.00	269.5	156.100	0.2434	0.0016	20.00	269.6	156.100	0.2474	0.0016	20.00
273.5	149.400	0.2165	0.0014	20.00	273.55	149.400	0.2151	0.0014	20.00	273.55	149.400	0.2125	0.0014	20.00	273.5	149.400	0.2120	0.0014	20.00	273.6	149.400	0.2149	0.0014	20.00
277.5	142.700	0.2033	0.0014	20.00	277.55	142.700	0.1827	0.0013	20.00	277.55	142.700	0.1941	0.0014	20.00	277.5	142.700	0.1861	0.0013	20.00	277.6	142.700	0.1843	0.0013	20.00
281.5	136.100	0.1871	0.0014	20.00	281.55	136.100	0.1595	0.0012	20.00	281.55	136.100	0.1754	0.0013	20.00	281.5	136.100	0.1571	0.0012	20.00	281.6	136.100	0.1623	0.0012	20.00
285.5	129.400	0.1736	0.0013	20.00	285.55	129.400	0.1408	0.0011	20.00	285.55	129.400	0.1626	0.0013	20.00	285.5	129.400	0.1361	0.0011	20.00	285.6	129.400	0.1480	0.0011	20.00
289.5	122.700	0.1595	0.0013	20.00	289.55	122.750	0.1309	0.0011	20.00	289.55	122.700	0.1460	0.0012	20.00	289.5	122.700	0.1246	0.0010	20.00	289.6	122.750	0.1352	0.0011	20.00
293.5	116.100	0.1466	0.0013	20.00	293.55	116.100	0.1147	0.0010	20.00	293.55	116.100	0.1353	0.0012	20.00	293.5	116.100	0.1135	0.0010	20.00	293.6	116.100	0.1221	0.0011	20.00
297.5	109.400	0.1323	0.0012	20.00	297.55	109.400	0.1055	0.0010	20.00	297.55	109.400	0.1192	0.0011	20.00	297.5	109.400	0.1018	0.0009	20.00	297.6	109.400	0.1099	0.0010	20.00
301.5	102.800	0.1170	0.0011	20.00	301.55	102.800	0.0908	0.0009	20.00	301.55	102.750	0.1102	0.0011	20.00	301.5	102.700	0.0855	0.0008	20.00	301.6	102.800	0.0966	0.0009	20.00
305.5	96.090	0.1040	0.0011	20.00	305.55	96.095	0.0808	0.0008	20.00	305.55	96.085	0.0950	0.0010	20.00	305.5	96.085	0.0759	0.0008	20.00	305.6	96.093	0.0850	0.0009	20.00
309.5	89.425	0.0890	0.0010	20.00	309.55	89.430	0.0682	0.0008	20.00	309.55	89.420	0.0845	0.0009	20.00	309.5	89.430	0.0609	0.0007	20.00	309.6	89.430	0.0724	0.0008	20.00
313.5	82.760	0.0765	0.0009	20.00	313.55	82.765	0.0595	0.0007	20.00	313.55	82.755	0.0684	0.0008	20.00	313.5	82.760	0.0523	0.0006	20.00	313.6	82.765	0.0614	0.0007	20.00
317.5	76.095	0.0635	0.0008	20.00	317.55	76.105	0.0461	0.0006	20.00	317.55	76.090	0.0585	0.0008	20.00	317.5	76.095	0.0393	0.0005	20.00	317.6	76.100	0.0494	0.0006	20.00
321.5	69.430	0.0523	0.0008	20.00	321.55	69.670	0.0456	0.0007	20.00	321.55	69.430	0.0453	0.0007	20.00	321.5	69.930	0.0366	0.0005	20.00	321.6	69.438	0.0406	0.0006	20.00
325.5	62.770	0.0390	0.0006	20.00	325.55	63.270	0.0245	0.0004	20.00	325.55	62.850	0.0449	0.0007	20.00	325.5	63.270	0.0210	0.0003	20.00	325.6	63.275	0.0278	0.0004	20.00
329.5	56.615	0.0290	0.0005	20.00	329.55	56.605	0.0140	0.0002	20.00	329.55	56.595	0.0230	0.0004	20.00	329.5	56.635	0.0145	0.0003	20.00	329.6	56.605	0.0170	0.0003	20.00
333.5	49.935	0.0161	0.0003	20.00	333.55	50.245	0.0076	0.0002	20.00	333.55	49.935	0.0154	0.0003	20.00	333.5	50.290	0.0062	0.0001	20.00	333.6	50.108	0.0112	0.0002	20.00
337.5	43.480	0.0104	0.0002	20.00	337.55	43.710	0.0064	0.0001	20.00	337.55	43.695	0.0041	0.0001	20.00	337.5	43.700	0.0073	0.0002	20.00	337.6	43.708	0.0037	0.0001	20.00
341.5	37.040	0.0070	0.0002	20.00	341.55	37.050	0.0155	0.0004	20.00	341.55	37.035	0.0093	0.0003	20.00	341.5	37.040	0.0183	0.0005	20.00	341.6	37.045	0.0138	0.0004	20.00
345.5	30.375	0.0184	0.0006	20.00	345.55	30.060	0.0352	0.0012	20.00	345.55	30.365	0.0205	0.0007	20.00	345.5	29.895	0.0339	0.0011	20.00	345.6	30.325	0.0306	0.0010	20.00
349.5	23.370	0.0399	0.0017	20.00	349.55	23.220	0.0362	0.0016	20.00	349.55	23.200	0.0328	0.0014	20.00	349.5	23.215	0.0374	0.0016	20.00	349.6	23.220	0.0349	0.0015	20.00
353.5	16.555	0.0418	0.0025	20.00	353.55	16.560	0.0462	0.0028	20.00	353.55	16.545	0.0431	0.0026	20.00	353.5	16.550	0.0465	0.0028	20.00	353.6	16.558	0.0450	0.0027	20.00
357.5	9.887	0.0530	0.0054	20.00	357.55	9.895	0.0553	0.0056	20.00	357.55	9.883	0.0535	0.0054	20.00	357.5	9.887	0.0557	0.0056	20.00	357.6	9.892	0.0546	0.0055	20.00
361.5	3.224	0.0637	0.0198	20.00	361.55	3.230	0.0644	0.0200	20.00	361.55	3.219	0.0638	0.0198	20.00	361.5	3.223	0.0646	0.0201	20.00	361.6	3.227	0.0641	0.0199	20.00

Note: t – time (s); γ – shear rate (1/s); τ – shear stress (Pa); η – dynamic shear viscosity (Pa.s); T – temperature (°C).

Table S3: Rheological data of **B** dispersions (for carrier systems No. 1–15).

1					2					3					4					5				
t (s)	γ (1/s)	τ (Pa)	η (Pa.s)	T (°C)	t (s)	γ (1/s)	τ (Pa)	η (Pa.s)	T (°C)	t (s)	γ (1/s)	τ (Pa)	η (Pa.s)	T (°C)	t (s)	γ (1/s)	τ (Pa)	η (Pa.s)	T (°C)	t (s)	γ (1/s)	τ (Pa)	η (Pa.s)	T (°C)
143.6	4.102	0.0735	0.0179	19.99	128.15	4.104	0.0790	0.0193	19.99	124.4	4.103	0.0733	0.0179	20.01	124.4	4.102	0.0768	0.0187	20.00	124.45	4.104	0.0777	0.0189	20.00
147.6	10.770	0.0807	0.0075	19.99	132.15	10.770	0.0917	0.0085	19.99	128.4	10.770	0.0809	0.0075	20.01	128.4	10.770	0.0888	0.0082	20.00	128.45	10.770	0.0808	0.0075	20.00
151.6	17.430	0.0881	0.0051	19.99	136.15	17.430	0.1057	0.0061	19.99	132.4	17.430	0.0890	0.0051	20.01	132.4	17.430	0.1012	0.0058	20.00	132.45	17.430	0.0887	0.0051	20.00
155.6	24.095	0.0970	0.0040	19.99	140.15	24.095	0.1201	0.0050	19.99	136.4	24.100	0.0965	0.0040	20.01	136.4	24.095	0.1145	0.0048	20.00	136.45	24.100	0.0963	0.0040	20.00
159.6	30.760	0.1049	0.0034	19.99	144.15	30.760	0.1346	0.0044	20.00	140.4	30.760	0.1053	0.0034	20.01	140.4	30.760	0.1271	0.0041	20.00	140.45	30.760	0.1045	0.0034	20.00
163.6	37.425	0.1144	0.0031	19.99	148.15	37.420	0.1493	0.0040	20.00	144.4	37.420	0.1129	0.0030	20.01	144.4	37.425	0.1407	0.0038	20.00	144.45	37.420	0.1121	0.0030	20.00
167.6	44.090	0.1213	0.0028	20.00	152.15	44.090	0.1645	0.0037	20.00	148.4	44.090	0.1239	0.0028	20.01	148.4	44.090	0.1530	0.0035	20.00	148.45	44.090	0.1224	0.0028	20.00
171.6	50.755	0.1310	0.0026	19.99	156.15	50.750	0.1796	0.0035	20.00	152.4	50.750	0.1308	0.0026	20.01	152.4	50.750	0.1667	0.0033	20.00	152.45	50.750	0.1284	0.0025	20.00
175.6	57.420	0.1394	0.0024	19.99	160.15	57.420	0.1957	0.0034	20.00	156.4	57.415	0.1426	0.0025	20.01	156.4	57.415	0.1787	0.0031	20.00	156.45	57.415	0.1419	0.0025	20.00
179.6	64.085	0.1504	0.0023	19.99	164.15	64.085	0.2106	0.0033	20.00	160.4	64.080	0.1497	0.0023	20.01	160.4	64.080	0.1936	0.0030	20.00	160.45	64.080	0.1477	0.0023	20.00
183.6	70.745	0.1600	0.0023	19.99	168.15	70.745	0.2263	0.0032	20.00	164.4	70.745	0.1598	0.0023	20.01	164.4	70.745	0.2071	0.0029	20.00	164.45	70.740	0.1563	0.0022	20.00
187.6	77.410	0.1669	0.0022	19.99	172.15	77.410	0.2415	0.0031	20.00	168.4	77.410	0.1672	0.0022	20.01	168.4	77.410	0.2215	0.0029	20.00	168.45	77.410	0.1615	0.0021	20.00
191.6	84.075	0.1788	0.0021	19.99	176.15	84.070	0.2573	0.0031	20.00	172.4	84.075	0.1747	0.0021	20.01	172.4	84.070	0.2373	0.0028	20.00	172.45	84.075	0.1694	0.0020	20.00
195.6	90.740	0.1847	0.0020	19.99	180.15	90.735	0.2742	0.0030	20.00	176.4	90.735	0.1903	0.0021	20.01	176.4	90.740	0.2487	0.0027	20.01	176.45	90.735	0.1843	0.0020	20.00
199.6	97.400	0.1972	0.0020	19.99	184.15	97.400	0.2902	0.0030	20.00	180.4	97.405	0.1984	0.0020	20.01	180.4	97.400	0.2640	0.0027	20.00	180.45	97.400	0.1932	0.0020	20.00
203.6	104.100	0.2065	0.0020	19.99	188.15	104.100	0.3076	0.0030	20.00	184.4	104.100	0.2116	0.0020	20.01	184.4	104.100	0.2774	0.0027	20.00	184.45	104.100	0.2066	0.0020	20.00
207.6	110.700	0.2197	0.0020	19.99	192.15	110.700	0.3231	0.0029	20.00	188.4	110.700	0.2162	0.0020	20.01	188.4	110.700	0.2949	0.0027	20.00	188.45	110.700	0.2098	0.0019	20.00
211.6	117.400	0.2338	0.0020	19.99	196.15	117.400	0.3397	0.0029	20.00	192.4	117.400	0.2271	0.0019	20.00	192.4	117.400	0.3103	0.0026	20.00	192.45	117.400	0.2315	0.0020	20.00
215.6	124.100	0.2558	0.0021	20.00	200.15	124.100	0.3576	0.0029	20.00	196.4	124.100	0.2540	0.0020	20.00	196.4	124.100	0.3232	0.0026	20.00	196.45	124.100	0.2651	0.0021	20.00
219.6	130.700	0.2876	0.0022	20.00	204.15	130.700	0.3745	0.0029	20.00	200.4	130.700	0.2860	0.0022	20.00	200.4	130.700	0.3389	0.0026	20.00	200.45	130.700	0.2930	0.0022	20.00
223.6	137.400	0.3135	0.0023	20.00	208.15	137.400	0.3927	0.0029	20.00	204.4	137.400	0.3175	0.0023	20.00	204.4	137.400	0.3531	0.0026	20.00	204.45	137.400	0.3222	0.0023	20.00
227.6	144.050	0.3432	0.0024	20.00	212.15	144.050	0.4089	0.0028	20.00	208.4	144.050	0.3376	0.0023	20.01	208.4	144.000	0.3722	0.0026	20.00	208.45	144.050	0.3192	0.0022	20.00
231.6	150.700	0.3559	0.0024	20.00	216.15	150.700	0.4267	0.0028	20.00	212.4	150.700	0.3448	0.0023	20.01	212.4	150.700	0.3888	0.0026	20.00	212.45	150.700	0.3376	0.0022	20.00
235.6	157.400	0.3637	0.0023	20.00	220.15	157.400	0.4453	0.0028	20.00	216.4	157.400	0.3706	0.0024	20.01	216.4	157.400	0.4023	0.0026	20.00	216.45	157.400	0.3625	0.0023	20.00
239.6	164.000	0.3860	0.0024	20.00	224.15	164.000	0.4634	0.0028	20.00	220.4	164.000	0.3902	0.0024	20.01	220.4	164.000	0.4167	0.0025	20.00	220.45	164.000	0.3836	0.0023	20.00
243.6	170.700	0.4096	0.0024	20.00	228.15	170.700	0.4828	0.0028	20.00	224.4	170.700	0.4157	0.0024	20.01	224.4	170.700	0.4328	0.0025	20.00	224.45	170.700	0.4083	0.0024	20.00
247.6	177.400	0.4363	0.0025	20.00	232.15	177.400	0.5000	0.0028	20.00	228.4	177.400	0.4334	0.0024	20.01	228.4	177.400	0.4599	0.0026	20.00	228.45	177.400	0.4208	0.0024	20.00
251.6	184.000	0.4628	0.0025	20.00	236.15	184.000	0.5192	0.0028	20.00	232.4	184.000	0.4566	0.0025	20.01	232.4	184.000	0.5107	0.0028	20.00	232.45	184.000	0.4376	0.0024	20.00
255.6	190.700	0.4784	0.0025	20.00	240.15	190.700	0.5398	0.0028	20.00	236.4	190.700	0.4869	0.0026	20.01	236.4	190.700	0.5496	0.0029	20.00	236.45	190.700	0.4692	0.0025	20.00
259.6	197.400	0.5095	0.0026	20.00	244.15	197.400	0.5628	0.0029	20.00	240.4	197.400	0.5074	0.0026	20.00	240.4	197.400	0.5936	0.0030	20.00	240.45	197.400	0.4862	0.0025	20.00
260.6	199.850	16.6300	0.0832	20.00	245.15	199.950	12.0960	0.0605	20.00	241.45	199.950	9.3565	0.0468	20.00	241.4	199.950	14.1655	0.0709	20.00	241.5	199.950	12.0715	0.0604	20.00
264.65	195.700	0.3655	0.0019	20.00	249.2	195.700	0.4206	0.0021	20.00	245.5	195.700	0.3539	0.0018	20.00	245.5	195.700	0.4423	0.0023	20.00	245.5	195.700	0.3437	0.0018	20.00
268.65	189.050	0.3449	0.0018	20.00	253.2	189.050	0.3945	0.0021	20.00	249.5	189.000	0.3200	0.0017	20.00	249.5	189.050	0.4046	0.0021	20.00	249.5	189.050	0.3140	0.0017	20.00
272.65	182.400	0.3180	0.0017	20.00	257.2	182.400	0.3759	0.0021	20.00	253.5	182.400	0.3022	0.0017	20.00	253.5	182.400	0.3593	0.0020	20.00	253.5	182.400	0.2942	0.0016	20.00
276.65	175.900	0.2992	0.0017	20.00	261.2	175.700	0.3568	0.0020	20.00	257.5	176.100	0.2783	0.0016	20.00	257.5	175.700	0.3243	0.0018	20.00	257.5	176.100	0.2681	0.0015	20.00
280.65	169.400	0.2676	0.0016	20.00	265.2	169.100	0.3361	0.0020	20.00	261.5	169.400	0.2643	0.0016	20.00	261.5	169.150	0.2960	0.0018	20.00	261.5	169.400	0.2552	0.0015	20.00
284.65	162.700	0.2469	0.0015	20.00	269.2	162.400	0.3187	0.0020	20.00	265.5	162.700	0.2402	0.0015	20.00	265.5	162.700	0.2725	0.0017	20.00	265.5	162.700	0.2313	0.0014	20.00
288.65	156.100	0.2226	0.0014	20.00	273.2	155.700	0.2978	0.0019	20.00	269.5	156.100	0.2232	0.0014	20.00	269.5	156.100	0.2540	0.0016	20.00	269.5	156.100	0.2129	0.0014	20.00
292.65	149.400	0.2059	0.0014	20.00	277.2	149.350	0.2844	0.0019	20.00	273.5	149.400	0.1952	0.0013	20.00	273.5	149.400	0.2420	0.0016	20.00	273.5	149.400	0.1877	0.0013	20.00
296.65	142.700	0.1825	0.0013	20.00	281.2	142.750	0.2642	0.0019	20.00	277.5	142.700	0.1794	0.0013	20.00	277.5	142.700	0.2232	0.0016	20.00	277.5	142.700	0.1746	0.0012	20.00
300.65	136.100	0.1610	0.0012	20.00	285.2	136.100	0.2492	0.0018	20.00	281.5	136.100	0.1575	0.0012	20.00	281.5	136.100	0.2081	0.0015	20.00	281.5	136.100	0.1558	0.0011	20.00
304.65	129.400	0.1376	0.0011	20.00	289.2	129.400	0.2304	0.0018	20.00	285.5	129.400	0.1353	0.0010	20.00	285.5	129.400	0.1925	0.0015	20.00	285.5	129.400	0.1389	0.0011	20.00
308.65	122.750	0.1117	0.0009	20.00	293.2	122.750	0.2149	0.0018	20.00	289.5	122.700	0.1146	0.0009	20.00	289.5	122.750	0.1756	0.0014	20.00	289.5	122.700	0.1188	0.0010	20.00
312.65	116.100	0.0884	0.0008	20.00	297.2	116.100	0.1960	0.0017	20.00	293.5	116.100	0.0945	0.0008	20.00	293.5	116.100	0.1601	0.0014	20.00	293.5	116.100	0.0987	0.0009	20.00
316.65	109.400	0.0750	0.0007	20.00	301.2	109.400	0.1808	0.0017	20.00	297.5	109.400	0.0816	0.0007	20.00	297.5	109.400	0.1457	0.0013	20.00	297.5	109.400	0.0761	0.0007	20.00
320.65	102.800	0.0672	0.0007	20.00	305.2	102.800	0.1629	0.0016	20.00	301.5	102.700	0.0662	0.0006	20.00	301.5	102.750	0.1341	0.0013	20.00	301.5	102.750	0.0600	0.0006	20.00
324.65	96.095	0.0559	0.0006	20.00	309.2	96.090	0.1479	0.0015	20.00	305.5	96.085	0.0580	0.0006	20.00	305.5	96.090	0.1185	0.0012	20.00	305.5	96.085	0.0516	0.0005	20.00
328.65	89.430	0.0486	0.0005	20.00	313.2	89.430	0.1314	0.0015	20.00	309.5	89.425	0.0443	0.0005	20.00	309.5	89.425	0.1068	0.0012	20.00	309.5	89.425	0.0388	0.0004	20.00
332.65	82.980	0.0484	0.0006	20.00	317.2	82.765	0.1168	0.0014	20.00	313.5	82.850	0.0466	0.0006	20.00	313.5	82.765	0.0916	0.0011	20.00	313.5	83.260	0.0302	0.0004	20.00
336.65	76.600	0.0296	0.0004	20.00	321.2	76.105	0.0997	0.0013	20.00	317.5	76.590	0.0259	0.0003	20.00	317.5	76.095	0.0794	0.0010	20.00	317.5	76.590	0.0209	0.0003	20.00
340.65	69.935	0.0178	0.0003	20.00	325.2	69.440	0.0838	0.0012	20.00	321.5	69.930	0.0205	0.0003	20.00	321.5	69.430	0.0631	0.0009	20.00	321.5	69.950	0.0169	0.0002	20.00
344.65	63.410	0.0119	0.0002	20.00	329.2	62.770	0.0682	0.0011	20.00	325.5	63.380	0.0112	0.0002	20.00	325.5	62.765	0.0508	0.0008	20.00	325.5	63.540	0.0101	0.0002	20.00
348.65	56.940	0.0076	0.0001	20.00	333.2	56.110	0.0526	0.0009	20.00	329.5	57.040	0.0028	0.0000	20.00	329.5	56.105	0.0359	0.0006	20.00	329.5	57.035	0.0014	0.0000	20.00
352.65	50.375	0.0068	0.0001	20.00	337.2	49.445	0.0367	0.0007	20.00	333.5	50.370	0.0088	0.0002	20.00	333.5	49.950	0.0253	0.0005	20.00	333.5	50.365	0.0120	0.0002	20.00

356.65	43.710	0.0165	0.0004	20.00	341.2	43.280	0.0257	0.0006	20.00	337.5	43.700	0.0158	0.0004	20.00	337.5	43.270	0.0113	0.0003	20.00	337.5	43.705	0.0188	0.0004	20.00
360.65	36.540	0.0275	0.0008	20.00	345.2	36.625	0.0116	0.0003	20.00	341.5	36.600	0.0342	0.0009	20.00	341.5	37.040	0.0035	0.0001	20.00	341.5	36.540	0.0275	0.0008	20.00
364.65	29.885	0.0342	0.0011	20.00	349.2	30.390	0.0044	0.0001	20.00	345.5	29.885	0.0337	0.0011	20.00	345.5	30.380	0.0123	0.0004	20.00	345.5	29.885	0.0359	0.0012	20.00
368.65	23.225	0.0427	0.0018	20.00	353.2	23.715	0.0185	0.0008	20.00	349.5	23.210	0.0413	0.0018	20.00	349.5	23.690	0.0291	0.0012	20.00	349.5	23.210	0.0432	0.0019	20.00
372.65	16.555	0.0503	0.0030	20.00	357.2	16.620	0.0401	0.0024	20.00	353.5	16.555	0.0494	0.0030	20.00	353.5	16.545	0.0385	0.0023	20.00	353.5	16.555	0.0506	0.0031	20.00
376.65	9.893	0.0577	0.0058	20.00	361.2	9.893	0.0476	0.0048	20.00	357.5	9.886	0.0573	0.0058	20.00	357.5	9.888	0.0505	0.0051	20.00	357.5	9.887	0.0581	0.0059	20.00
380.65	3.229	0.0649	0.0201	20.00	365.2	3.228	0.0616	0.0191	20.00	361.5	3.221	0.0649	0.0202	20.00	361.5	3.223	0.0627	0.0194	20.00	361.5	3.223	0.0653	0.0203	20.00
6					7					8					9					10				
t (s)	γ (1/s)	τ (Pa)	η (Pa.s)	T (°C)	t (s)	γ (1/s)	τ (Pa)	η (Pa.s)	T (°C)	t (s)	γ (1/s)	τ (Pa)	η (Pa.s)	T (°C)	t (s)	γ (1/s)	τ (Pa)	η (Pa.s)	T (°C)	t (s)	γ (1/s)	τ (Pa)	η (Pa.s)	T (°C)
124.35	4.102	0.0770	0.0188	20.00	124.35	4.103	0.0730	0.0178	20.00	124.35	4.101	0.0757	0.0185	20.00	124.55	4.103	0.0731	0.0178	20.00	124.4	4.096	0.0854	0.0209	20.00
128.35	10.770	0.0895	0.0083	20.00	128.35	10.770	0.0803	0.0075	20.00	128.35	10.770	0.0863	0.0080	20.00	128.55	10.770	0.0796	0.0074	20.00	128.4	10.765	0.0955	0.0089	20.00
132.35	17.430	0.1017	0.0058	20.00	132.35	17.430	0.0877	0.0050	20.00	132.35	17.430	0.0975	0.0056	20.00	132.55	17.430	0.0863	0.0049	20.00	132.4	17.425	0.1113	0.0064	20.00
136.35	24.100	0.1151	0.0048	20.00	136.35	24.100	0.0959	0.0040	20.00	136.35	24.095	0.1089	0.0045	20.00	136.55	24.100	0.0951	0.0039	20.00	136.4	24.095	0.1273	0.0053	20.00
140.35	30.760	0.1283	0.0042	20.00	140.35	30.760	0.1033	0.0034	20.00	140.35	30.760	0.1210	0.0039	20.00	140.55	30.760	0.1020	0.0033	20.00	140.4	30.755	0.1436	0.0047	20.00
144.35	37.425	0.1426	0.0038	20.00	144.35	37.420	0.1117	0.0030	20.00	144.35	37.425	0.1328	0.0035	20.00	144.55	37.425	0.1111	0.0030	20.00	144.4	37.420	0.1597	0.0043	20.00
148.35	44.090	0.1553	0.0035	20.00	148.35	44.090	0.1199	0.0027	20.00	148.35	44.090	0.1448	0.0033	20.00	148.55	44.090	0.1162	0.0026	20.00	148.4	44.085	0.1767	0.0040	20.00
152.35	50.755	0.1703	0.0034	20.00	152.35	50.750	0.1258	0.0025	20.00	152.35	50.750	0.1583	0.0031	20.00	152.55	50.750	0.1242	0.0024	20.00	152.4	50.750	0.1934	0.0038	20.00
156.35	57.420	0.1826	0.0032	20.00	156.35	57.420	0.1383	0.0024	20.00	156.35	57.420	0.1690	0.0029	20.00	156.55	57.420	0.1343	0.0023	20.00	156.4	57.410	0.2109	0.0037	20.00
160.35	64.085	0.1987	0.0031	20.00	160.35	64.085	0.1465	0.0023	20.00	160.35	64.080	0.1824	0.0028	20.00	160.55	64.090	0.1439	0.0022	20.00	160.4	64.075	0.2270	0.0035	20.00
164.35	70.745	0.2125	0.0030	20.00	164.35	70.745	0.1573	0.0022	20.00	164.35	70.745	0.1938	0.0027	20.00	164.55	70.750	0.1543	0.0022	20.00	164.4	70.740	0.2448	0.0035	20.00
168.35	77.410	0.2286	0.0030	20.00	168.35	77.410	0.1615	0.0021	20.00	168.35	77.410	0.2090	0.0027	20.00	168.55	77.410	0.1600	0.0021	20.00	168.4	77.405	0.2596	0.0034	20.00
172.35	84.075	0.2444	0.0029	20.00	172.35	84.080	0.1712	0.0020	20.00	172.35	84.070	0.2220	0.0026	20.00	172.55	84.070	0.1725	0.0021	20.00	172.4	84.065	0.2766	0.0033	20.00
176.35	90.740	0.2569	0.0028	20.00	176.35	90.740	0.1826	0.0020	20.00	176.35	90.735	0.2344	0.0026	20.00	176.55	90.740	0.1777	0.0020	20.00	176.4	90.730	0.2941	0.0032	20.00
180.35	97.400	0.2708	0.0028	20.00	180.35	97.400	0.1939	0.0020	20.00	180.35	97.400	0.2474	0.0025	20.00	180.55	97.400	0.1909	0.0020	20.00	180.4	97.395	0.3091	0.0032	20.00
184.35	104.100	0.2847	0.0027	20.00	184.35	104.100	0.2067	0.0020	20.00	184.35	104.100	0.2601	0.0025	20.00	184.55	104.100	0.2008	0.0019	20.00	184.4	104.100	0.3238	0.0031	20.00
188.35	110.700	0.3033	0.0027	20.00	188.35	110.700	0.2112	0.0019	20.00	188.35	110.700	0.2769	0.0025	20.00	188.55	110.700	0.2117	0.0019	20.00	188.4	110.700	0.3403	0.0031	20.00
192.35	117.400	0.3190	0.0027	20.00	192.35	117.400	0.2283	0.0019	20.00	192.35	117.400	0.2898	0.0025	20.00	192.55	117.400	0.2389	0.0020	20.00	192.4	117.400	0.3577	0.0030	20.00
196.35	124.100	0.3334	0.0027	20.00	196.35	124.100	0.2599	0.0021	20.00	196.35	124.100	0.3046	0.0025	20.00	196.55	124.100	0.2617	0.0021	20.00	196.4	124.050	0.3762	0.0030	20.00
200.35	130.700	0.3470	0.0027	20.00	200.35	130.700	0.2898	0.0022	20.00	200.35	130.700	0.3180	0.0024	20.00	200.55	130.700	0.2918	0.0022	20.00	200.4	130.700	0.3945	0.0030	20.00
204.35	137.400	0.3611	0.0026	20.00	204.35	137.400	0.3191	0.0023	20.00	204.35	137.400	0.3318	0.0024	20.00	204.55	137.400	0.3154	0.0023	20.00	204.4	137.400	0.4099	0.0030	20.00
208.35	144.100	0.3792	0.0026	20.00	208.35	144.100	0.3303	0.0023	20.00	208.35	144.050	0.3501	0.0024	20.00	208.55	144.050	0.3212	0.0022	20.00	208.4	144.000	0.4276	0.0030	20.00
212.35	150.700	0.3931	0.0026	20.00	212.35	150.700	0.3388	0.0022	20.00	212.35	150.700	0.3647	0.0024	20.00	212.55	150.700	0.3433	0.0023	20.00	212.4	150.700	0.4452	0.0030	20.00
216.35	157.400	0.4072	0.0026	20.00	216.35	157.400	0.3588	0.0023	20.00	216.35	157.400	0.3813	0.0024	20.00	216.55	157.400	0.3559	0.0023	20.00	216.4	157.400	0.4633	0.0029	20.00
220.35	164.000	0.4243	0.0026	20.00	220.35	164.000	0.3831	0.0023	20.00	220.35	164.000	0.4036	0.0025	20.00	220.55	164.000	0.3810	0.0023	20.00	220.4	164.000	0.4808	0.0029	20.00
224.35	170.700	0.4408	0.0026	20.00	224.35	170.700	0.4099	0.0024	20.00	224.35	170.700	0.4459	0.0026	20.00	224.55	170.700	0.4048	0.0024	20.00	224.4	170.700	0.5001	0.0029	20.00
228.35	177.400	0.4645	0.0026	20.00	228.35	177.400	0.4240	0.0024	20.00	228.35	177.400	0.4902	0.0028	20.00	228.55	177.400	0.4233	0.0024	20.00	228.4	177.400	0.5163	0.0029	20.00
232.35	184.000	0.5067	0.0028	20.00	232.35	184.000	0.4472	0.0024	20.00	232.35	184.000	0.5292	0.0029	20.00	232.55	184.000	0.4460	0.0024	20.00	232.4	184.000	0.5360	0.0029	20.00
236.35	190.700	0.5485	0.0029	20.00	236.35	190.700	0.4790	0.0025	20.00	236.35	190.700	0.5661	0.0030	20.00	236.55	190.700	0.4607	0.0024	20.00	236.4	190.700	0.5571	0.0029	20.00
240.35	197.400	0.5929	0.0030	20.00	240.35	197.400	0.5027	0.0025	20.00	240.35	197.400	0.5763	0.0029	20.00	240.55	197.400	0.4880	0.0025	20.00	240.4	197.400	0.5766	0.0029	20.00
241.4	199.950	7.1105	0.0356	20.00	241.35	199.900	9.3924	0.0470	20.00	241.35	199.950	17.9750	0.0899	20.00	241.55	199.850	13.6950	0.0685	20.00	241.4	199.950	13.1890	0.0660	20.00
245.45	195.700	0.4417	0.0023	20.00	245.45	195.700	0.3552	0.0018	20.00	245.4	195.700	0.4215	0.0022	20.00	245.65	195.700	0.3422	0.0017	20.00	245.5	195.700	0.4297	0.0022	20.00
249.45	189.000	0.4031	0.0021	20.00	249.45	189.000	0.3262	0.0017	20.00	249.4	189.000	0.3929	0.0021	20.00	249.65	189.050	0.3194	0.0017	20.00	249.5	189.100	0.4072	0.0022	20.00
253.45	182.400	0.3592	0.0020	20.00	253.45	182.400	0.2993	0.0016	20.00	253.4	182.400	0.3661	0.0020	20.00	253.65	182.450	0.2902	0.0016	20.00	253.5	182.400	0.3845	0.0021	20.00
257.45	175.700	0.3241	0.0018	20.00	257.45	176.050	0.2869	0.0016	20.00	257.4	175.700	0.3366	0.0019	20.00	257.65	176.100	0.2714	0.0015	20.00	257.5	175.700	0.3641	0.0021	20.00
261.45	169.100	0.2976	0.0018	20.00	261.45	169.400	0.2608	0.0015	20.00	261.4	169.100	0.2998	0.0018	20.00	261.65	169.400	0.2489	0.0015	20.00	261.5	169.100	0.3449	0.0020	20.00
265.45	162.700	0.2798	0.0017	20.00	265.45	162.700	0.2370	0.0015	20.00	265.4	162.700	0.2650	0.0016	20.00	265.65	162.700	0.2304	0.0014	20.00	265.5	162.400	0.3264	0.0020	20.00
269.45	156.100	0.2614	0.0017	20.00	269.45	156.100	0.2215	0.0014	20.00	269.4	156.100	0.2350	0.0015	20.00	269.65	156.100	0.2137	0.0014	20.00	269.5	155.700	0.3081	0.0020	20.00
273.45	149.400	0.2476	0.0017	20.00	273.45	149.400	0.1936	0.0013	20.00	273.4	149.400	0.2178	0.0015	20.00	273.65	149.400	0.1944	0.0013	20.00	273.5	149.200	0.2953	0.0020	20.00
277.45	142.700	0.2293	0.0016	20.00	277.45	142.700	0.1778	0.0012	20.00	277.4	142.700	0.2023	0.0014	20.00	277.65	142.750	0.1777	0.0012	20.00	277.5	142.700	0.2731	0.0019	20.00
281.45	136.100	0.2142	0.0016	20.00	281.45	136.100	0.1533	0.0011	20.00	281.4	136.100	0.1883	0.0014	20.00	281.65	136.100	0.1515	0.0011	20.00	281.5	136.100	0.2550	0.0019	20.00
285.45	129.400	0.1981	0.0015	20.00	285.45	129.400	0.1357	0.0010	20.00	285.4	129.400	0.1732	0.0013	20.00	285.65	129.400	0.1361	0.0011	20.00	285.5	129.400	0.2374	0.0018	20.00
289.45	122.750	0.1819	0.0015	20.00	289.45	122.700	0.1118	0.0009	20.00	289.4	122.700	0.1597	0.0013	20.00	289.65	122.750	0.1107	0.0009	20.00	289.5	122.700	0.2196	0.0018	20.00
293.45	116.100	0.1653	0.0014	20.00	293.45	116.100	0.0948	0.0008	20.00	293.4	116.100	0.1444	0.0012	20.00	293.65	116.100	0.0948	0.0008	20.00	293.5	116.100	0.2030	0.0017	20.00
297.45	109.400	0.1510	0.0014	20.00	297.45	109.400	0.0743	0.0007	20.00	297.4	109.400	0.1311	0.0012	20.00	297.65	109.400	0.0693	0.0006	20.00	297.5	109.400	0.1854	0.0017	20.00
301.45	102.750	0.1381	0.0013	20.00	301.45	102.700	0.0635	0.0006	20.00	301.4	102.750	0.1171	0.0011	20.00	301.65	102.800	0.0614	0.0006	20.00	301.5	102.800	0.1694	0.0016	20.00
305.45	96.090	0.1224	0.0013	20.00	305.45	96.085	0.0528	0.0005	20.00	305.4	96.090	0.1043	0.0011	20.00	305.65	96.090	0.0487	0.0005	20.00	305.5	96.090	0.1524	0.0016	20.00
309.45	89.425	0.1096	0.0012	20.00	309.45	89.420	0.0417	0.0005	20.00	309.4	89.425	0.0909	0.0010	20.00	309.65	89.435	0.0409	0.0005	20.00	309.5	89.425	0.1363	0.0015	20.00
313.45	82.760	0.0947	0.0011	20.00	313.45	83.255	0.0331	0.0004	20.00	313.4	82.765	0.0782	0.0009	20.00	313.65	83.270	0.0282	0.0003	20.00	313.5	82.765	0.1193	0.0014	20.00
317.45	76.095	0.0813	0.0011	20.00	317.45	76.580	0.0233	0.0003	20.00	317.4	76.095	0.0648	0.0009	20.00	317.65	76.595	0.0227	0.0003	20.00	317.5	76.100	0.1032	0.0014	20.00

321.45	69.430	0.0653	0.0009	20.00	321.45	69.930	0.0161	0.0002	20.00	321.4	69.435	0.0522	0.0008	20.00	321.65	69.960	0.0138	0.0002	20.00	321.5	69.435	0.0873	0.0013	20.00
325.45	62.770	0.0523	0.0008	20.00	325.45	63.435	0.0088	0.0001	20.00	325.4	62.770	0.0397	0.0006	20.00	325.65	63.515	0.0116	0.0002	20.00	325.5	62.765	0.0716	0.0011	20.00
329.45	56.105	0.0370	0.0007	20.00	329.45	57.035	0.0006	0.0000	20.00	329.4	56.610	0.0301	0.0005	20.00	329.65	57.035	0.0016	0.0000	20.00	329.5	56.110	0.0560	0.0010	20.00
333.45	49.945	0.0262	0.0005	20.00	333.45	50.365	0.0095	0.0002	20.00	333.4	49.940	0.0161	0.0003	20.00	333.65	50.375	0.0094	0.0002	20.00	333.5	49.440	0.0404	0.0008	20.00
337.45	43.270	0.0117	0.0003	20.00	337.45	43.700	0.0165	0.0004	20.00	337.4	43.460	0.0100	0.0002	20.00	337.65	43.710	0.0174	0.0004	20.00	337.5	43.160	0.0348	0.0008	20.00
341.45	37.035	0.0039	0.0001	20.00	341.45	36.525	0.0272	0.0007	20.00	341.4	37.045	0.0062	0.0002	20.00	341.65	36.535	0.0289	0.0008	20.00	341.5	36.605	0.0110	0.0003	20.00
345.45	30.380	0.0125	0.0004	20.00	345.45	29.875	0.0350	0.0012	20.00	345.4	30.380	0.0178	0.0006	20.00	345.65	29.885	0.0357	0.0012	20.00	345.5	30.385	0.0016	0.0001	20.00
349.45	23.690	0.0289	0.0012	20.00	349.45	23.210	0.0433	0.0019	20.00	349.4	23.260	0.0364	0.0016	20.00	349.65	23.220	0.0447	0.0019	20.00	349.5	23.715	0.0174	0.0007	20.00
353.45	16.545	0.0388	0.0023	20.00	353.45	16.545	0.0501	0.0030	20.00	353.4	16.550	0.0415	0.0025	20.00	353.65	16.555	0.0507	0.0031	20.00	353.5	16.690	0.0416	0.0025	20.00
357.45	9.887	0.0509	0.0051	20.00	357.45	9.882	0.0580	0.0059	20.00	357.4	9.890	0.0525	0.0053	20.00	357.65	9.893	0.0585	0.0059	20.00	357.5	9.890	0.0474	0.0048	20.00
361.45	3.224	0.0628	0.0195	20.00	361.45	3.218	0.0653	0.0203	20.00	361.4	3.225	0.0633	0.0196	20.00	361.65	3.229	0.0653	0.0202	20.00	361.5	3.226	0.0617	0.0191	20.00
11					12					13					14					15				
t (s)	γ (1/s)	τ (Pa)	η (Pa.s)	T (°C)	t (s)	γ (1/s)	τ (Pa)	η (Pa.s)	T (°C)	t (s)	γ (1/s)	τ (Pa)	η (Pa.s)	T (°C)	t (s)	γ (1/s)	τ (Pa)	η (Pa.s)	T (°C)	t (s)	γ (1/s)	τ (Pa)	η (Pa.s)	T (°C)
124.45	4.100	0.0760	0.0185	20.00	124.4	4.102	0.0781	0.0190	19.99	124.4	4.101	0.0783	0.0191	20.00	124.45	4.103	0.0764	0.0186	19.99	124.42	4.102	0.0771	0.0188	19.99
128.45	10.770	0.0871	0.0081	20.00	128.4	10.770	0.0907	0.0084	19.99	128.4	10.770	0.0918	0.0085	20.00	128.45	10.770	0.0865	0.0080	19.99	128.42	10.770	0.0880	0.0082	19.99
132.45	17.430	0.0988	0.0057	20.00	132.4	17.430	0.1031	0.0059	19.99	132.4	17.430	0.1042	0.0060	20.00	132.45	17.430	0.0971	0.0056	20.00	132.42	17.430	0.0993	0.0057	19.99
136.45	24.095	0.1105	0.0046	20.00	136.4	24.095	0.1162	0.0048	19.99	136.4	24.100	0.1169	0.0049	20.00	136.45	24.100	0.1072	0.0044	20.00	136.42	24.098	0.1109	0.0046	19.99
140.45	30.760	0.1232	0.0040	20.00	140.4	30.760	0.1281	0.0042	20.00	140.4	30.760	0.1295	0.0042	20.00	140.45	30.760	0.1186	0.0039	20.00	140.42	30.760	0.1220	0.0040	19.99
144.45	37.430	0.1354	0.0036	20.00	144.4	37.425	0.1413	0.0038	20.00	144.4	37.420	0.1420	0.0038	20.00	144.45	37.425	0.1292	0.0035	20.00	144.42	37.426	0.1338	0.0036	19.99
148.45	44.090	0.1486	0.0034	20.00	148.4	44.090	0.1524	0.0035	20.00	148.4	44.090	0.1554	0.0035	20.00	148.45	44.090	0.1414	0.0032	20.00	148.42	44.090	0.1451	0.0033	20.00
152.45	50.750	0.1622	0.0032	20.00	152.4	50.750	0.1652	0.0033	20.00	152.4	50.750	0.1676	0.0033	20.00	152.45	50.750	0.1543	0.0030	20.00	152.42	50.750	0.1570	0.0031	20.00
156.45	57.420	0.1733	0.0030	20.00	156.4	57.420	0.1791	0.0031	20.00	156.4	57.415	0.1821	0.0032	20.00	156.45	57.420	0.1641	0.0029	20.00	156.42	57.418	0.1691	0.0029	20.00
160.45	64.075	0.1875	0.0029	20.00	160.4	64.085	0.1921	0.0030	20.00	160.4	64.080	0.1940	0.0030	20.00	160.45	64.080	0.1764	0.0028	20.00	160.42	64.084	0.1823	0.0028	20.00
164.45	70.745	0.1998	0.0028	20.00	164.4	70.745	0.2071	0.0029	20.00	164.4	70.745	0.2086	0.0029	20.00	164.45	70.740	0.1869	0.0026	20.00	164.42	70.746	0.1940	0.0027	20.00
168.45	77.410	0.2167	0.0028	20.00	168.4	77.410	0.2188	0.0028	20.00	168.4	77.410	0.2213	0.0029	20.00	168.45	77.410	0.2030	0.0026	20.00	168.42	77.410	0.2060	0.0027	20.00
172.45	84.070	0.2301	0.0027	20.00	172.4	84.075	0.2335	0.0028	20.00	172.4	84.070	0.2344	0.0028	20.00	172.45	84.070	0.2130	0.0025	20.00	172.42	84.072	0.2197	0.0026	20.00
176.45	90.735	0.2431	0.0027	20.00	176.4	90.740	0.2446	0.0027	20.00	176.4	90.735	0.2510	0.0028	20.00	176.45	90.735	0.2253	0.0025	20.00	176.42	90.740	0.2313	0.0025	20.00
180.45	97.400	0.2533	0.0026	20.00	180.4	97.400	0.2612	0.0027	20.00	180.4	97.405	0.2650	0.0027	20.00	180.45	97.400	0.2372	0.0024	20.00	180.42	97.404	0.2453	0.0025	20.00
184.45	104.100	0.2653	0.0025	20.00	184.4	104.100	0.2752	0.0026	20.00	184.4	104.100	0.2809	0.0027	20.00	184.45	104.100	0.2484	0.0024	20.00	184.42	104.100	0.2588	0.0025	20.00
188.45	110.700	0.2827	0.0026	20.00	188.4	110.700	0.2907	0.0026	20.00	188.4	110.700	0.2931	0.0026	20.00	188.45	110.700	0.2657	0.0024	20.00	188.42	110.700	0.2727	0.0025	20.00
192.45	117.400	0.2931	0.0025	20.00	192.4	117.400	0.3075	0.0026	20.00	192.4	117.400	0.3081	0.0026	20.00	192.45	117.400	0.2767	0.0024	20.00	192.42	117.400	0.2873	0.0024	20.00
196.45	124.100	0.3091	0.0025	20.00	196.4	124.100	0.3188	0.0026	20.00	196.4	124.100	0.3255	0.0026	20.00	196.45	124.100	0.2926	0.0024	20.00	196.42	124.100	0.3002	0.0024	20.00
200.45	130.700	0.3222	0.0025	20.00	200.4	130.700	0.3342	0.0026	20.00	200.4	130.700	0.3412	0.0026	20.00	200.45	130.700	0.3038	0.0023	20.00	200.42	130.700	0.3153	0.0024	20.00
204.45	137.400	0.3363	0.0024	20.00	204.4	137.400	0.3486	0.0025	20.00	204.4	137.400	0.3587	0.0026	20.00	204.45	137.400	0.3158	0.0023	20.00	204.42	137.400	0.3296	0.0024	20.00
208.45	144.050	0.3560	0.0025	20.00	208.4	144.100	0.3659	0.0025	20.00	208.4	144.050	0.3705	0.0026	20.00	208.45	144.100	0.3343	0.0023	20.00	208.42	144.060	0.3452	0.0024	20.00
212.45	150.700	0.3682	0.0024	20.00	212.4	150.700	0.3834	0.0025	20.00	212.4	150.700	0.3867	0.0026	20.00	212.45	150.700	0.3482	0.0023	20.00	212.42	150.700	0.3614	0.0024	20.00
216.45	157.400	0.3837	0.0024	20.00	216.4	157.400	0.3954	0.0025	20.00	216.4	157.400	0.4058	0.0026	20.00	216.45	157.400	0.3796	0.0024	20.00	216.42	157.400	0.3765	0.0024	20.00
220.45	164.000	0.4067	0.0025	20.00	220.4	164.000	0.4144	0.0025	20.00	220.4	164.000	0.4227	0.0026	20.00	220.45	164.000	0.4138	0.0025	20.00	220.42	164.000	0.4040	0.0025	20.00
224.45	170.700	0.4504	0.0026	20.00	224.4	170.700	0.4329	0.0025	20.00	224.4	170.700	0.4413	0.0026	20.00	224.45	170.700	0.4460	0.0026	20.00	224.42	170.700	0.4471	0.0026	20.00
228.45	177.400	0.4927	0.0028	20.00	228.4	177.400	0.4683	0.0026	20.00	228.4	177.400	0.4594	0.0026	20.00	228.45	177.400	0.4866	0.0027	20.00	228.42	177.400	0.4862	0.0027	20.00
232.45	184.000	0.5311	0.0029	20.00	232.4	184.000	0.5139	0.0028	20.00	232.4	184.000	0.5017	0.0027	20.00	232.45	184.000	0.5081	0.0028	20.00	232.42	184.000	0.5256	0.0029	20.00
236.45	190.700	0.5670	0.0030	20.00	236.4	190.700	0.5523	0.0029	20.00	236.4	190.700	0.5484	0.0029	20.00	236.45	190.700	0.5345	0.0028	20.00	236.42	190.700	0.5634	0.0030	20.00
240.45	197.400	0.5769	0.0029	20.00	240.4	197.400	0.5954	0.0030	20.00	240.4	197.400	0.5898	0.0030	20.00	240.45	197.400	0.5547	0.0028	20.00	240.42	197.400	0.5740	0.0029	20.00
241.45	199.900	18.0850	0.0904	20.00	241.4	199.900	7.1420	0.0357	20.00	241.4	199.950	5.7405	0.0287	20.00	241.45	199.950	9.2940	0.0465	20.00	241.44	199.920	14.3604	0.0718	20.00
245.55	195.700	0.4193	0.0021	20.00	245.5	195.700	0.4446	0.0023	20.00	245.5	195.700	0.4396	0.0022	20.00	245.55	195.700	0.4033	0.0021	20.00	245.52	195.700	0.4174	0.0021	20.00
249.55	189.000	0.3919	0.0021	20.00	249.5	189.050	0.4067	0.0022	20.00	249.5	189.050	0.3945	0.0021	20.00	249.55	189.050	0.3775	0.0020	20.00	249.52	189.040	0.3924	0.0021	20.00
253.55	182.400	0.3668	0.0020	20.00	253.5	182.400	0.3601	0.0020	20.00	253.5	182.400	0.3565	0.0020	20.00	253.55	182.400	0.3523	0.0019	20.00	253.52	182.400	0.3631	0.0020	20.00
257.55	175.700	0.3371	0.0019	20.00	257.5	175.700	0.3255	0.0019	20.00	257.5	175.700	0.3163	0.0018	20.00	257.55	175.700	0.3256	0.0019	20.00	257.52	175.700	0.3354	0.0019	20.00
261.55	169.050	0.2981	0.0018	20.00	261.5	169.150	0.2967	0.0018	20.00	261.5	169.100	0.2984	0.0018	20.00	261.55	169.100	0.2990	0.0018	20.00	261.52	169.100	0.2987	0.0018	20.00
265.55	162.700	0.2649	0.0016	20.00	265.5	162.700	0.2707	0.0017	20.00	265.5	162.700	0.2768	0.0017	20.00	265.55	162.700	0.2664	0.0016	20.00	265.52	162.700	0.2654	0.0016	20.00
269.55	156.100	0.2317	0.0015	20.00	269.5	156.100	0.2540	0.0016	20.00	269.5	156.100	0.2626	0.0017	20.00	269.55	156.100	0.2322	0.0015	20.00	269.52	156.100	0.2345	0.0015	20.00
273.55	149.400	0.2150	0.0014	20.00	273.5	149.400	0.2406	0.0016	20.00	273.5	149.400	0.2420	0.0016	20.00	273.55	149.400	0.2046	0.0014	20.00	273.52	149.400	0.2172	0.0015	20.00
277.55	142.700	0.1980	0.0014	20.00	277.5	142.700	0.2236	0.0016	20.00	277.5	142.700	0.2288	0.0016	20.00	277.55	142.700	0.1828	0.0013	20.00	277.52	142.700	0.2015	0.0014	20.00
281.55	136.100	0.1868	0.0014	20.00	281.5	136.100	0.2050	0.0015	20.00	281.5	136.100	0.2111	0.0016	20.00	281.55	136.100	0.1729	0.0013	20.00	281.52	136.100	0.1856	0.0014	20.00
285.55	129.400	0.1705	0.0013	20.00	285.5	129.400	0.1907	0.0015	20.00	285.5	129.400	0.1969	0.0015	20.00	285.55	129.400	0.1569	0.0012	20.00	285.52	129.400	0.1715	0.0013	20.00
289.55	122.700	0.1578	0.0013	20.00	289.5	122.750	0.1726	0.0014	20.00	289.5	122.750	0.1824	0.0015	20.00	289.55	122.750	0.1456	0.0012	20.00	289.52	122.700	0.1563	0.0013	20.00
293.55	116.100	0.1402	0.0012	20.00	293.5	116.100	0.1603	0.0014	20.00	293.5	116.100	0.1690	0.0015	20.00	293.55	116.100	0.1286	0.0011	20.00	293.52	116.100	0.1429	0.0012	20.00
297.55	109.400	0.1292	0.0012	20.00	297.5	109.400	0.1430	0.0013	20.00	297.5	109.400	0.1532	0.0014	20.00	297.55	109.400	0.1180	0.0011	20.00	297.52	109.400	0.1284	0.0012	20.00
301.55	102.750	0.1146	0.0011	20.00	301.5	102.750	0.1324	0.0013	20.00	301.5	102.750	0.1358	0.0013	20.00	301.55	102.750	0.1037	0.0010	20.00	301.52	102.760	0.1163	0.0011	20.00

305.55	96.085	0.1024	0.0011	20.00	305.5	96.095	0.1161	0.0012	20.00	305.5	96.085	0.1221	0.0013	20.00	305.55	96.095	0.0926	0.0010	20.00	305.52	96.088	0.1023	0.0011	20.00
309.55	89.415	0.0893	0.0010	20.00	309.5	89.425	0.1042	0.0012	20.00	309.5	89.425	0.1056	0.0012	20.00	309.55	89.425	0.0803	0.0009	20.00	309.52	89.424	0.0900	0.0010	20.00
313.55	82.755	0.0782	0.0009	20.00	313.5	82.765	0.0870	0.0011	20.00	313.5	82.765	0.0925	0.0011	20.00	313.55	82.760	0.0699	0.0008	20.00	313.52	82.760	0.0758	0.0009	20.00
317.55	76.095	0.0638	0.0008	20.00	317.5	76.095	0.0761	0.0010	20.00	317.5	76.095	0.0780	0.0010	20.00	317.55	76.100	0.0563	0.0007	20.00	317.52	76.094	0.0640	0.0008	20.00
321.55	69.430	0.0503	0.0007	20.00	321.5	69.435	0.0613	0.0009	20.00	321.5	69.435	0.0670	0.0010	20.00	321.55	69.430	0.0434	0.0006	20.00	321.52	69.430	0.0507	0.0007	20.00
325.55	62.760	0.0381	0.0006	20.00	325.5	62.770	0.0492	0.0008	20.00	325.5	62.775	0.0524	0.0008	20.00	325.55	63.180	0.0405	0.0006	20.00	325.52	62.768	0.0390	0.0006	20.00
329.55	56.605	0.0259	0.0005	20.00	329.5	56.105	0.0356	0.0006	20.00	329.5	56.105	0.0408	0.0007	20.00	329.55	56.605	0.0204	0.0004	20.00	329.52	56.610	0.0288	0.0005	20.00
333.55	49.935	0.0138	0.0003	20.00	333.5	49.945	0.0253	0.0005	20.00	333.5	49.785	0.0371	0.0007	20.00	333.55	49.980	0.0120	0.0002	20.00	333.52	49.932	0.0163	0.0003	20.00
337.55	43.545	0.0088	0.0002	20.00	337.5	43.275	0.0119	0.0003	20.00	337.5	43.275	0.0159	0.0004	20.00	337.55	43.715	0.0011	0.0000	20.00	337.52	43.512	0.0108	0.0002	20.00
341.55	37.035	0.0072	0.0002	20.00	341.5	37.050	0.0021	0.0001	20.00	341.5	36.915	0.0097	0.0003	20.00	341.55	37.045	0.0104	0.0003	20.00	341.52	37.042	0.0062	0.0002	20.00
345.55	30.375	0.0193	0.0006	20.00	345.5	30.375	0.0119	0.0004	20.00	345.5	30.380	0.0094	0.0003	20.00	345.55	30.375	0.0243	0.0008	20.00	345.52	30.374	0.0177	0.0006	20.00
349.55	23.225	0.0353	0.0015	20.00	349.5	23.715	0.0263	0.0011	20.00	349.5	23.710	0.0227	0.0010	20.00	349.55	23.210	0.0326	0.0014	20.00	349.52	23.330	0.0369	0.0016	20.00
353.55	16.550	0.0425	0.0026	20.00	353.5	16.545	0.0380	0.0023	20.00	353.5	16.545	0.0367	0.0022	20.00	353.55	16.550	0.0434	0.0026	20.00	353.52	16.550	0.0409	0.0025	20.00
357.55	9.885	0.0529	0.0054	20.00	357.5	9.890	0.0497	0.0050	20.00	357.5	9.890	0.0488	0.0049	20.00	357.55	9.889	0.0531	0.0054	20.00	357.52	9.887	0.0519	0.0052	20.00
361.55	3.221	0.0636	0.0197	20.00	361.5	3.226	0.0621	0.0192	20.00	361.5	3.226	0.0618	0.0192	20.00	361.55	3.224	0.0635	0.0197	20.00	361.52	3.223	0.0630	0.0196	20.00

Note: t – time (s); γ – shear rate (1/s); τ – shear stress (Pa); η – dynamic shear viscosity (Pa.s); T – temperature (°C).
